# Laminin alpha 5 regulates mammary gland remodeling through luminal cell differentiation and Wnt4-mediated epithelial crosstalk

**DOI:** 10.1242/dev.199281

**Published:** 2021-06-15

**Authors:** Johanna I. Englund, Alexandra Ritchie, Leander Blaas, Hanne Cojoc, Nalle Pentinmikko, Julia Döhla, Sharif Iqbal, Manuel Patarroyo, Pekka Katajisto

**Affiliations:** 1 Institute of Biotechnology, Helsinki Institute of Life Sciences (HiLIFE), 00014 University of Helsinki, Helsinki, Finland; 2 Department of Biosciences and Nutrition, Karolinska Institutet, 141 83 Huddinge, Sweden; 3 Department of Microbiology, Tumor and Cell Biology, Karolinska Institutet, 171 11 Solna, Sweden; 4 Faculty of Biological and Environmental Sciences, 00014 University of Helsinki, Helsinki, Finland; 5 Department of Cell and Molecular Biology, Karolinska Institutet, 171 77 Solna, Sweden

**Keywords:** Laminin alpha 5, Extracellular matrix, Basement membrane, Mammary gland, Luminal epithelial cell, Wnt4

## Abstract

Epithelial attachment to the basement membrane (BM) is essential for mammary gland development, yet the exact roles of specific BM components remain unclear. Here, we show that Laminin α5 (*Lama5*) expression specifically in the luminal epithelial cells is necessary for normal mammary gland growth during puberty, and for alveologenesis during pregnancy. *Lama5* loss in the keratin 8-expressing cells results in reduced frequency and differentiation of hormone receptor expressing (HR^+^) luminal cells. Consequently, Wnt4-mediated crosstalk between HR^+^ luminal cells and basal epithelial cells is compromised during gland remodeling, and results in defective epithelial growth. The effects of *Lama5* deletion on gland growth and branching can be rescued by Wnt4 supplementation in the *in vitro* model of branching morphogenesis. Our results reveal a surprising role for BM-protein expression in the luminal mammary epithelial cells, and highlight the function of Lama5 in mammary gland remodeling and luminal differentiation.

## Abstract (precis)

**Summary:** Luminal mammary epithelial cells produce basement membrane laminin α5 necessary for mammary epithelial growth and differentiation. Laminin α5 loss compromises hormone receptor-positive luminal cell function and Wnt4-mediated crosstalk between epithelial cells.

## INTRODUCTION

Postnatal development of the mammary gland occurs by branching and elongation during puberty, and by extensive remodeling during pregnancy (Macias and Hinck, 2012; Paine and Lewis, 2017). The fully formed adult mammary epithelium consists of a bilayered duct, in which apicobasally polarized keratin 8/18^+^ luminal cells, which can be subdivided into hormone receptor-expressing and non-expressing luminal cells (HR^+^ and HR^−^, respectively), face the ductal lumen and are surrounded by keratin 5/14^+^ basal myoepithelial cells (Macias and Hinck, 2012; Adriance et al., 2005).

The mammary microenvironment contributes to various properties of mammary epithelial cells (MECs), including proliferation, survival and differentiation (Inman et al., 2015). In particular, attachment to the specialized layer of the extracellular matrix (ECM) called the basement membrane (BM) is crucial for cultured mammary epithelial cells and for mammary gland morphogenesis *in vivo* (Inman et al., 2015; Weaver et al., 1997; LaBarge et al., 2009). The BM acts as a physical barrier separating the epithelium from the stroma and as a scaffold supporting epithelial adhesion and tissue architecture (Yurchenco, 2011). Moreover, the BM regulates tissue homeostasis by supplying cells with growth factors and other signaling molecules, and by regulating their availability to the cells (Yurchenco, 2011).

Laminins are the main components of the BM that together with collagen IV form self-assembling networks, which provide epithelial cells an anchoring platform and various survival and differentiation signals (Hohenester and Yurchenco, 2013). Laminins are heterotrimers consisting of α, β and γ subunits, which are expressed in a tissue-specific and temporally controlled manner (Ahmed and Ffrench-Constant, 2016). Several laminin isoforms have been detected in the mammary gland, and earlier studies suggest that laminin-111 (containing α1, β1 and γ1 subunits), laminin-332 and laminin-511/521 are the most common forms in the adult glands (Goddard et al., 2016; Gudjonsson et al., 2002; Prince et al., 2002), yet laminin-211 and laminin-411/421 are also found. Microarray studies and later single-cell sequencing efforts have indicated high expression of Lama1 and Lama3 in the basal cells, with Lama5 being highest in luminal cells (Lim et al., 2010; Bach et al., 2017; Tabula Muris Consortium, 2018). However, what the spatial and temporal expression patterns of these laminins within intact tissue are and what their exact roles in mammary development and function are is unclear.

We set out to study the expression pattern and function of different laminin isoforms in the mouse mammary gland. We demonstrate that Lama5 produced by luminal cells is necessary for normal mammary gland growth and development during both puberty and pregnancy. Mechanistically, we show that Lama5 loss alters differentiation of HR^+^ luminal MECs, and consequently their Wnt4-mediated interactions with basal cells during gland remodeling. Our results reveal that Lama5 acts as a key microenvironmental effector of mammary gland function by orchestrating HR^+^ luminal cell specification during puberty and pregnancy.

## RESULTS

### Differential expression of laminin isoforms in distinct cell types of the mammary epithelium

**Fig. 1.**
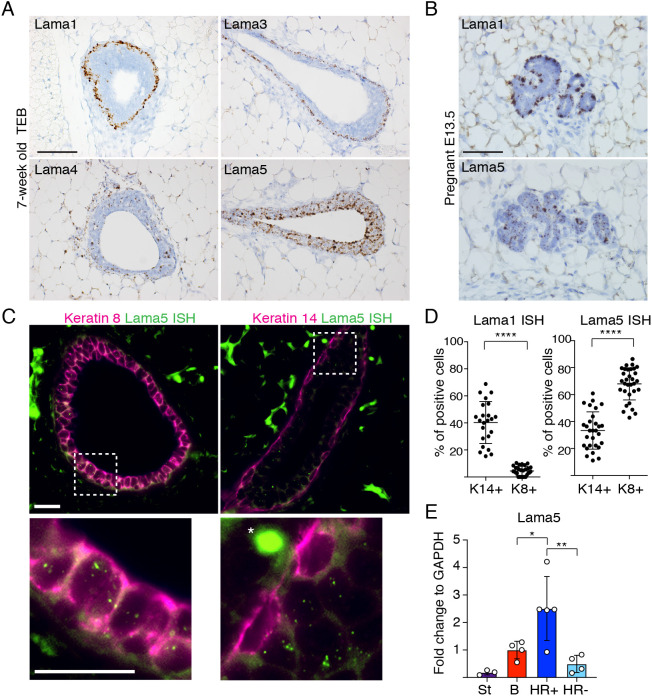
**Expression of laminin α chain isoforms in the mammary gland is cell type-specific.** (A) Representative images of RNA ISH performed with probes for Lama1, 3, 4 and 5 on TEBs of 7-week-old pubertal mammary glands. (B) Representative images of RNA ISH of mammary glands from embryonic day 13.5 pregnant mice performed with probes for Lama1 and 5 on TEBs. (C) Representative images of Lama5 RNA ISH coupled with either K8 or K14 antibody staining. Asterisk denotes background staining. The dashed boxes indicate the magnified regions in the lower panels. (D) Quantification of the percentage of K14^+^ or K8^+^ cells showing either Lama1 or Lama5 expression. Lama1 quantification in K14^+^ and K8^+^ cells (K14, *n*=23 ducts; K8, *n*=28 ducts; from three animals). Lama5 quantification in K14^+^ (*n*=29) and K8^+^ cells (*n*=31) ducts from three animals. Data points correspond to individual ducts analyzed. (E) qRT-PCR analysis of Lama5 expression compared with GAPDH in populations of stromal (St), basal (B), luminal HR^+^ and luminal HR^−^ cells FACS sorted from mammary glands (St, *n*=3; B, *n*=4; HR^+^, *n*=5; HR^−^, *n*=4 mice analyzed). Data are mean±s.d. An unpaired two-tailed Student's *t*-test was used to compare indicated groups (*P<0.05, **P<0.01, ****P<0.0001). Scale bars: 50 µm (A); 50 µm (B); 20 µm.

To explore the role of specific laminins in the mammary gland, we first studied their expression during the pubertal gland expansion, which is marked by terminal end bud (TEB) structures (Macias and Hinck, 2012). Using *in situ* hybridization (ISH) to detect the expression of *Lama1*, *3*, *4* and *5* (encoding the laminin α1, α3, α4 and α5 subunits), we observed that both *Lama1* and *3* were expressed by the basal cells of growing TEBs (Fig. 1A) and established ducts (Fig. S1A). *Lama3* expression was also detected in some luminal cells of the mature ducts (Fig. S1A, red arrowheads). In striking contrast, *Lama5* was strongly expressed by luminal epithelial cells, particularly in TEBs, and *Lama4* showed widespread expression also in stroma. As *Lama1* and *Lama5* showed the most cell type-specific expression patterns, we next analyzed their expression during the pregnancy-induced gland remodeling. Although separation of the two cellular layers is less evident in the differentiated alveoli, *Lama1* expression appeared to be restricted to basal cells and *Lama5* was mostly restricted to luminal cells also during pregnancy (Fig. 1B). To quantitatively address expression of *Lama1* and *Lama5* in the luminal and basal MECs, we combined laminin ISH with keratin 8 (K8, also known as Krt8) and keratin 14 (K14, also known as Krt14) antibody staining in the pubertal glands (Fig. 1C; Fig. S1B,C). Whereas *Lama1* was strictly expressed by basal (K8^−^ and K14^+^) cells, *Lama5* was significantly enriched in luminal (K8^+^ and K14^−^) epithelial cells with some expression in a subset of basal cells (Fig. 1D; Fig. S1C). Moreover, by further subdividing CD29^low^/CD24^+^ luminal cells into HR^−^ and HR^+^ with fluorescence-activated cell sorting (FACS; Fig. S1D) by cell surface markers Sca1 (also known as Ly6a) and CD49b (Shehata et al., 2012), we found that HR-expressing luminal cells are the major source for Lama5 in the mammary epithelium (Fig. 1E).

The expression of the BM component Lama5 in luminal cells was surprising as luminal cells are reported to have only very limited contacts with the BM (Smith and Medina, 1988; Lafkas et al., 2013). However, the Pan-laminin staining that we observed mostly in the periductal region suggested that the luminally expressed laminin proteins are also deposited on the BM surrounding the gland (Fig. S1E). Providing a potential route for luminal cells to secrete laminins into the BM, we detected contacts between luminal cells and the BM (Fig. S1F) in tamoxifen-induced *Lgr6-CreERT2:Rosa26-tdTomato* mice in which clones of exclusively luminal or basal cells can be visualized (Blaas et al., 2016). Taken together, these results indicate that laminin isoforms have distinct expression patterns within the mammary gland (summarized in Fig. S1G), and also infer that the luminal epithelial cells, in addition to the BM-adjacent basal cells, may contribute to the laminin pool of the BM.

### Laminin α5 is required for normal pubertal mammary gland morphogenesis

**Fig. 2.**
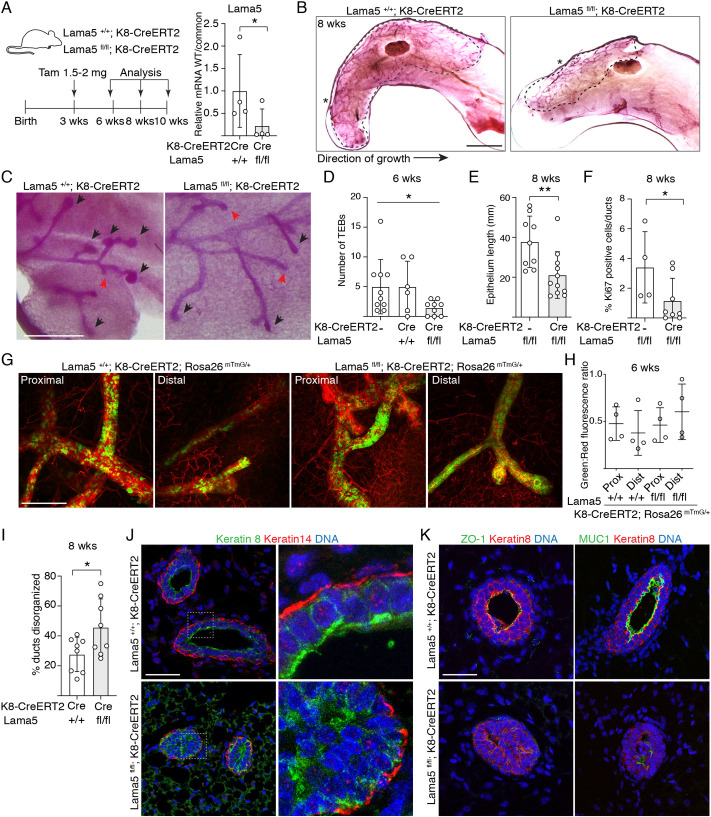
**Luminal laminin α5 is required for pubertal growth of the mammary epithelium.** (A) Schematic showing the outline of tamoxifen induction of K8-CreERT2 during puberty, and qPCR analysis of Lama5 mRNA expression in luminal MECs (+/+ and fl/fl, *n*=4 samples analyzed) by using wild-type (WT) allele-specific primers compared with primers common for both + and flox alleles (common). (B) Representative images of carmine alum-stained #4 mammary glands of 8-week-old *Lama5^+/+^;K8-CreERT2* and *Lama^fl/fl^;K8-CreERT2* treated with tamoxifen. The dashed line shows the outline of ductal growth and the asterisks mark the beginning of epithelium. (C) Representative images of TEBs and ductal ends (marked by black arrowheads and red arrows, respectively) in 6-week-old transgenic mice. (D) Quantification of the number of TEBs in 6-week-old transgenic mice analyzed from #4 mammary glands (*K8-CreERT2*, *n*=10; *Lama5^+/+^;K8-CreERT2*, *n*=6, *Lama5^fl/fl^;K8-CreERT2*, *n*=9 glands analyzed). (E) Quantification of epithelium length in 8-week-old transgenic mice analyzed from #4 mammary glands (*Lama5^fl/fl^;* *n*=9, *Lama5^fl/fl^;K8-CreERT2*, *n*=11 glands analyzed). (F) Quantification of Ki67 positivity in mammary glands of 8-week-old transgenic mice (*Lama5^fl/fl^;* *n*=4; *Lama5^fl/fl^;K8-CreERT2*, *n*=8 individuals analyzed). (G) Representative images of #4 mammary glands of 6-week-old *Lama5^+/+^;K8-CreERT2;R26^mTmG/+^* and *Lama5^fl/fl^;K8-CreERT2;R26^mTmG/+^* mice. Images show proximal (closer to the beginning of epithelium) and distal parts of the duct. (H) Quantification of green to red fluorescence ratio in proximal and distal parts of the duct in *Lama5^+/+^;K8-CreERT2;R26^mTmG/+^* and *Lama5^fl/fl^;K8-CreERT2;R26^mTmG/+^* mice (+/+ and fl/fl, both proximal and distal part, *n*=4 glands quantified). (I) Quantification of epithelial disorganization in ducts of 8-week-old *Lama5^+/+^;K8-CreERT2* (*n*=8) and *Lama5^fl/fl^;K8-CreERT2* (*n*=8) animals. (J) Representative immunofluorescence images of 8-week-old *Lama5^+/+^;K8-CreERT2* and *Lama5^fl/fl^;K8-CreERT2* glands immunostained with K8 and K14 antibodies. Boxes indicate the magnified regions in the right panels. (K) Representative immunofluorescence images of 8-week-old *Lama5^+/+^;K8-CreERT2* and *Lama5^fl/fl^;K8-CreERT2* glands immunostained with ZO-1 or MUC1, and K8 antibodies. Data points indicate analysis of individual mice. Data are mean±s.d. *P<0.05, **P<0.01 [unpaired two-tailed Student's *t*-test (A,E,I) or Welch's ANOVA test (D)]. Scale bars: 5 mm (B); 0.5 mm (C); 200 µm (G); 50 µm (J,K).

To address the functional role of the luminally expressed *Lama5*, we generated *Lama5^fl/fl^;K8-CreERT2* mice (Nguyen et al., 2005; Van Keymeulen et al., 2011), which allow tamoxifen-inducible deletion of *Lama5* in luminal epithelial cells. To determine the effects of *Lama5* loss during puberty, we injected 3-week-old female mice with 1.5-2 mg of tamoxifen, and analyzed glands at 6, 8 or 10 weeks of age (Fig. 2A). Consistent with previous reports (Shehata et al., 2014; Scheele et al., 2017), we observed a significant decrease in epithelial growth and TEB number even with such a low tamoxifen dose, forbidding use of higher doses (Fig. S2A). To validate effective recombination, we first analyzed allelic frequencies in FACS-sorted luminal and basal epithelial cells 3 days after tamoxifen treatment (Fig. S2B), and in bulk epithelial cells 3 weeks after tamoxifen (Fig. S2C). Recombined *Lama5* allele was observed only from the luminal epithelial cells, and a high level of recombination was evident 3 weeks after tamoxifen (Fig. S2B,C). Moreover, allele-specific analysis of mRNA levels (Fig. S2D) revealed an 88% reduction in the relative abundance of wild-type mRNA in luminal epithelial cells sorted from tamoxifen-treated 8-week-old *Lama5^fl/fl^;K8-CreERT2* mice compared with *Lama^+/+^;K8-CreERT2* controls (Fig. 2A; Fig. S2E). Thus, stable and cell type-specific deletion of Lama5 was achieved in the majority of the luminal cells, even with the lower tamoxifen dose used.

*Lama5* deletion in luminal MECs led to delayed development of the mammary epithelium (Fig. 2B), which was accompanied by a reduction in TEB size and number (Fig. 2C,D). Consequently, the extent of the epithelial network was significantly reduced and contained fewer Ki-67^+^ cells at 8 weeks (Fig. 2E,F). However, when *Lama5* was deleted in the post-pubertal mature glands of 8-week-old mice and analyzed at 12 weeks of age, no difference in the ductal end number was observed (Fig. S2F,G). These data suggest that although *Lama5* is required for mammary epithelium growth and morphogenesis during pubertal remodeling, loss of *Lama5* in adult mammary glands has little effect on adult homeostasis.

To investigate whether the defective gland growth upon *Lama5* targeting was due to impaired function of the Lama5-deleted luminal cells, or due to their loss, we crossed *Lama5^fl/fl^;K8-CreERT2* mice with the *R26-mTmG* reporter line (Muzumdar et al., 2007). Immunofluorescence analysis of the glands 3 weeks post tamoxifen showed comparable frequencies of recombined cells in *Lama5^fl/fl^;K8-CreERT2;R26^mT/mG^* and control mice (Fig. 2G,H; Fig. S2H). Although recombination frequency of the reporter and *Lama5* loci may differ (Vooijs et al., 2001), these results are in line with our analysis of recombination efficiency (Fig. S2E), and indicate that *Lama5*-deficient cells are not lost and contribute to blunted epithelial growth.

Interestingly, although parts of the ducts appeared ultrastructurally normal, histological analysis of *Lama5^fl/fl^;K8-CreERT2* glands at 8 weeks revealed a frequent loss of normal tissue architecture at certain gland regions. The regional variability may reflect the development of ducts before and after tamoxifen-induced *Lama5* deletion, but even in blinded analysis of the full mammary gland, aberrant ducts were significantly more frequent in Lama5-deficient mice than in control mice (Fig. 2I). In the aberrant ducts, K8^+^ luminal cells were atypically organized into multiple layers (Fig. 2J). These observations suggested defects in cellular polarity in the *Lama5*-deficient epithelium, and we therefore inspected the localization and expression of apical polarity markers ZO-1 (also known as Tjp1) and MUC1 (Fig. 2K). We noticed that even in the aberrant *Lama5^fl/fl^;K8-CreERT2* ducts localization of ZO-1 and MUC1 was mostly marking the apical surfaces facing the ductal lumens, similarly to controls (Fig. 2K). However, *Lama5^fl/fl^;K8-CreERT2* ducts showed great variability in intensity and localization of the polarity markers, suggesting that loss of Lama5 from the luminal epithelium affects luminal cell polarity but the polarity is not completely lost. Interestingly, both ZO-1 and MUC1 also exhibited a dramatic reduction in general staining intensity in the *Lama5*-deficient ducts. MUC1 is a differentiation marker of luminal mammary epithelial cells (Shehata et al., 2012), raising the possibility of defective luminal differentiation in *Lama5*-deleted glands. In conclusion, Lama5 in the luminal epithelial cells is necessary for normal pubertal mammary gland growth and properly polarized duct architecture.

### Loss of laminin α5 compromises HR^+^ cell differentiation

**Fig. 3.**
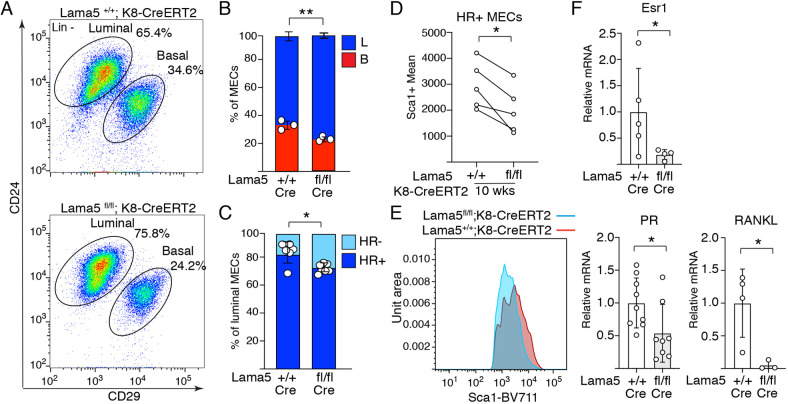
**Laminin α5 is needed for HR^+^ luminal epithelial cell identity.** (A) Representative CD24/CD29 FACS plot showing relative amounts of lineage marker negative luminal and basal MECs from 10-week-old *Lama5^fl/fl^;K8-CreERT2* and control animals. Gating strategy for the same sample is shown in Fig. S3A. (B,C) Quantification of the percentages of basal and luminal MECs (+/+, *n*=3; fl/fl, *n*=3 individuals analyzed) and (C) luminal HR^+^ and HR^−^ subpopulations from 10-week-old *Lama5^fl/fl^;K8-CreERT2* and control mice (+/+, *n*=6; fl/fl, *n*=6 individuals analyzed). Data points indicate samples analyzed from individual mice. (D) Mean intensity of Sca1^+^ luminal population from *Lama5^fl/fl^;K8-CreERT2* and control mice. Data show mean from *n*=5 separate experiments, in which 3-4 individual animals were analyzed per group. Data points indicate mean values from five separate experiments. (E) Representative Sca1-BV711 histograms of individual *Lama5^+/+^;K8-CreERT2* and *Lama5^fl/fl^;K8-CreERT2* mice. (F) qRT-PCR analysis of Esr1, PR and RANKL expression compared with GAPDH in luminal MECs from 10-week-old *Lama5^fl/fl^;K8-CreERT2* and control mice (Esr1 +/+, *n*=5; fl/fl, *n*=4; PR +/+, *n*=9; fl/fl, *n*=8; RANKL +/+, *n*=4; fl/fl, *n*=3). Data points indicate samples analyzed from individual mice. Data are mean±s.d. *P<0.05, **P<0.01 [unpaired two-tailed Student's *t*-test (B,C,F); paired *t*-test (D)].

The aberrant organization and reduction in luminal cell differentiation marker MUC1 prompted us to investigate cellular ratios of the *Lama5*-deficient mammary epithelium. We noticed a significant increase in luminal (CD29^low^/CD24^+^) to basal (CD29^hi^/CD24^+^) cell ratio in *Lama5^fl/fl^;K8-CreERT2* glands in comparison with controls (Fig. 3A,B; Fig. S3A,B). Interestingly, we also noted a 1.7-fold relative increase in the Sca1^−^/CD49b^+^ luminal cell subpopulation comprising HR^−^ cells (Fig. 3C) (Shehata et al., 2012; Sleeman et al., 2007) in Lama5-lacking animals. Moreover, the remaining HR^+^ cells expressed Sca1 on a lower level (Fig. 3D,E). This indicated that prepubertal loss of *Lama5* compromises specifically the pool of HR^+^ cells during pubertal gland growth. To further investigate whether the expression of HR^+^ luminal epithelial differentiation markers are altered in *Lama5*-deficient glands, we performed qPCR on estrogen receptor alpha (*Esr1*), progesterone receptor (PR, also known as Pgr) and Receptor Activator of Nuclear factor Kappa B ligand (RANKL, also known as Tnfsf11). Expression of all three markers of HR^+^ differentiation in the *Lama5*-targeted luminal cells was reduced significantly and beyond the level expected based on the observed reduction in HR^+^ cell frequency (Fig. 3F, compare with Fig. 3C). These data indicate that *Lama5* is indeed necessary for HR^+^ luminal cell differentiation and for maintaining a normal ratio of epithelial cells.

### Laminin α5 is necessary for normal alveologenesis

**Fig. 4.**
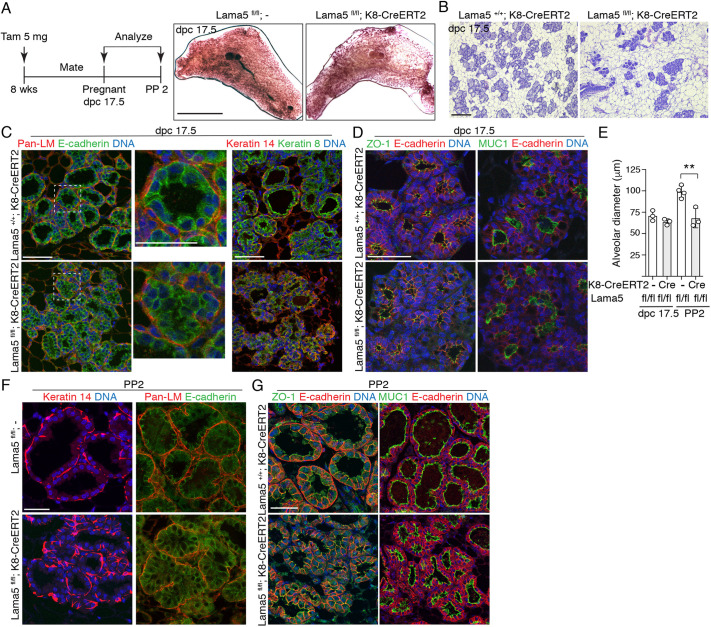
**Laminin α5 is essential for pregnancy-induced growth and differentiation of the mammary gland.** (A) Schematic showing the experimental outline of tamoxifen induction of K8-CreERT2 before pregnancy. Representative images of carmine alum-stained #4 mammary glands of 17.5 dpc pregnant *Lama^fl/fl^;-* and *Lama^fl/fl^;K8-CreERT2* animals. (B) Representative H&E images of 17.5 dpc pregnant *Lama5^+/+^;K8-CreERT2* and *Lama5^fl/fl^;K8-CreERT2* mice. (C) Representative immunofluorescence images of 17.5 dpc pregnant *Lama5^+/+^;K8-CreERT2* and *Lama5^fl/fl^;K8-CreERT2* glands immunostained with Pan-laminin and E-cadherin, or K8 and K14 antibodies. Square shows area of inset. (D) Representative immunofluorescence images of 17.5 dpc pregnant *Lama5^+/+^;K8-CreERT2* and *Lama5^fl/fl^;K8-CreERT2* glands immunostained with ZO-1 or MUC1, and E-cadherin antibodies. (E) Quantification of the alveolar diameter of 17.5 dpc (+/+, *n*=3; fl/fl, *n*=3 individuals analyzed) and PP2 mice (+/+, *n*=4; fl/fl, *n*=3 individuals analyzed). *N*=3-4 individual animals. Data points represent mean alveolar diameter quantified from individual animals. (F) Representative immunofluorescence images of PP2 mammary glands immunostained with K14 or Pan-laminin and E-cadherin antibodies. (G) Representative immunofluorescence images of PP2 mammary glands immunostained with ZO-1 or MUC1, and E-cadherin antibodies. Data are mean±s.d. **P<0.01 (unpaired two-tailed Student's *t*-test). Scale bars: 10 mm (A); 200 µm (B); 50 µm (C,D,F,G).

We next assessed the role of *Lama5* in the pregnancy-induced growth and functional differentiation of the mammary gland. *Lama5* was deleted from the luminal cells of 8-week-old female mice 1-2 weeks before pregnancy, and glands were analyzed 17.5 days post coitum (dpc; Fig. 4A; Fig. S4A). Alveolar development was dramatically reduced in *Lama5*-deficient glands, as evidenced by fewer and less densely packed alveoli compared with control glands (Fig. 4A,B; Fig. S4B). Moreover, *Lama5*-deficient alveoli often failed to form round structures, and were instead devoid of distinct lumen and exhibited aberrant organization of both luminal and basal cells (Fig. 4C). However, the apicobasal polarity of *Lama5*-deficient glands appeared only modestly affected at this stage of pregnancy (Fig. 4D) yet, intriguingly, also at this stage, MUC1 staining intensity was reduced. Analysis at postpartum day 2 (PP2) revealed that alveoli in *Lama5*-deleted glands were significantly smaller and exhibited disorganized cellular organization, even though Pan-laminin staining indicated that the BM remained intact (Fig. 4E,F). Again, cell polarity was largely unaffected, as demonstrated by staining of ZO-1 and MUC1 (Fig. 4G). However, the Lama5-deficient females often had less pups, which at this stage can also affect gland morphology. Taken together, our characterization of the mouse model with luminal-specific *Lama5* deletion demonstrates an important *in vivo* role for *Lama5* in luminal MECs during the key stages of mammary gland development and function.

### *Lama5*-deficient HR^+^ luminal MECs are unable to support basal cells

Ductal elongation during puberty and alveologenesis during pregnancy both require accurate interplay between the luminal and basal MECs (Macias and Hinck, 2012; Inman et al., 2015). HR^+^ luminal MECs are considered to orchestrate mammary growth and development by responding to systemic hormonal cues and transforming them into paracrine signals (Tanos et al., 2012). Accordingly, prepubertal loss of *Esr1* from luminal cells impedes the development of the mammary epithelium during puberty (Mallepell et al., 2006; Feng et al., 2007), and *Esr1* loss during pregnancy results in defective alveologenesis and milk production (Feng et al., 2007). As *Lama5* expression was highest in the luminal HR^+^ cells (Fig. 1E), and luminal *Lama5* loss reduced HR^+^ cell frequency and differentiation, as well as *Esr1*, *PR*, and *RANKL* expression (Fig. 3C-E), we hypothesized that defects in the growth and differentiation of Lama5 lacking mammary epithelium are due to compromised function of HR^+^ cells.

**Fig. 5.**
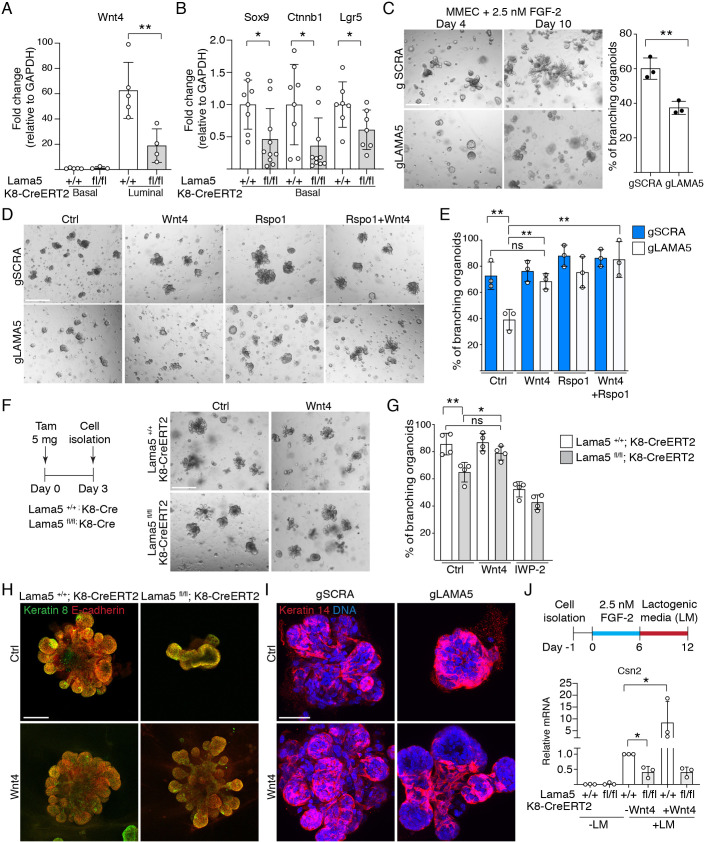
**Lama5-deficient luminal MECs are unable to support basal MECs due to defective Wnt signaling.** (A) qRT-PCR analysis of Wnt4 expression compared with GAPDH in basal (CD29^hi^/CD24^+^) and luminal (CD29^low^/CD24^+^) MECs FACS sorted from 8-10-week-old *Lama5^+/+^;K8-CreERT2* and *Lama5^fl/fl^;K8-CreERT2* glands treated with tamoxifen starting from 3 weeks of age (+/+ luminal and basal, fl/fl basal, *n*=3; fl/fl luminal, *n*=4 samples analyzed). (B) qRT-PCR analysis of Sox9, Ctnnb1 and Lgr5 expression compared with GAPDH in basal MECs from 10-week-old *Lama5^fl/fl^;K8-CreERT2* and control mice (Sox9 and Ctnnb1 +/+, *n*=8; fl/fl, *n*=10; Lgr5 +/+, *n*=7; fl/fl, *n*=7 samples analyzed). In A and B, data points indicate RNA samples analyzed from individual mice. (C) Primary MMECs carrying either LAMA5 or SCRA CRISPR guides (gLAMA5 and gSCRA) grown in Matrigel in the presence of 2.5 nM FGF-2 for 4 or 10 days, and quantification of the percentage of branching organoids at day 10 (gSCRA, *n*=3; gLAMA, *n*=3). (D) gLAMA5 or gSCRA expressing primary MMECs grown in Matrigel in the presence of 2.5 nM FGF-2 and either Wnt4, Rspo1 or both ligands for 7 days. (E) Quantification of the percentage of branching organoids at day 7 grown as in D (all treatments *n*=3). (F) Schematic showing the outline of tamoxifen induction and representative images. MECs were isolated from 10-14-week-old *Lama5^+/+^;K8-CreERT2* and *Lama5^fl/fl^;K8-CreERT2* mice treated with tamoxifen 3 days earlier, and grown in Matrigel in the presence of 2.5 nM FGF-2 and either Wnt4 or IWP-2 for 7 days. (G) Quantification of the percentage of branching organoids at day 7 grown as in F (all treatments *n*=4). In C, E and G, data points indicate RNA samples analysed from individual mice. (H) Representative keratin 8 and E-cadherin immunofluorescence images of primary MMECs organoids treated as in F and grown for 7 days. (I) Representative keratin 14 immunofluorescence images of primary MMECs organoids expressing either gLAMA5 or gSCRA treated as in D and grown for 7 days. (J) Schematic showing the outline of organoid treatment with lactogenic medium. Quantitative PCR analysis of Csn2 gene expression in MECs isolated from 10-14-week-old *Lama5^+/+^;K8-CreERT2* and *Lama5^fl/fl^;K8-CreERT2* mice treated with tamoxifen 3 days earlier, and grown as described in the schematic with or without Wnt4 (all treatments *n*=3). Data are normalized to lactogenic medium-treated *Lama5^+/+^;K8-CreERT2* for each experiment. Data points indicate RNA samples analysed from independent organoid experiments. Data are mean±s.d. *P<0.05; **P<0.01; ns, not significant [unpaired one-tailed (J) or two-tailed (A,B,C,E,G) Student's *t*-test]. Scale bars: 100 µm (C,D,H); 50 µm (I).

Wnt4 is an important paracrine mediator that is expressed in the HR^+^ MECs in response to progesterone (Tanos et al., 2012). Together with R-spondin 1 (Rspo1) from HR^−^ luminal MECs, Wnt4 activates Wnt signaling in the basal cells to regulate growth of the mammary epithelium (Rajaram et al., 2015; Cai et al., 2014; Brisken et al., 2000). Interestingly, expression of *Wnt4* was significantly reduced in *Lama5-*deficient luminal cells, whereas Rspo1 showed only a trend of modest reduction (Fig. 5A; Fig. S5A). Moreover, the expression of Wnt-responsive genes *Sox9* and *Lgr5* regulated directly via transcriptional control (Blache et al., 2004; Schuijers and Clevers, 2012), or *Ctnnb1* regulated via mRNA stability (Bikkavilli and Malbon, 2010), was significantly reduced in the basal cells (Fig. 5B). Jointly these data indicate that defects in the HR^+^ cells after luminal *Lama5* loss does indeed compromise the luminal-basal interactions.

To directly test whether the altered paracrine Wnt signaling contributes to the defective growth of the mammary gland after luminal *Lama5* loss, we modeled gland growth *in vitro*. Ductal growth by the TEB structures can be modeled *in vitro* with FGF-2 supplemented three-dimensional cultures, in which MEC organoids form branching structures (Ewald et al., 2008). Under these conditions, lentiviral CRISPR/Cas9-mediated *Lama5* editing resulted in *Lama5* mRNA downregulation and a significant reduction in organoid branch formation and elongation (Fig. 5C; Fig. S5B,C), thereby resembling the effects of luminal *Lama5* deficiency *in vivo*. Strikingly, exogenous addition of Wnt4 and Rspo1 completely rescued the effects that *Lama5* deletion had on branching frequency (Fig. 5D,E). These effects are likely to reflect the general activation of the canonical Wnt-pathway as Wnt3a supplementation recapitulated the results seen with Wnt4 (Fig. S5D). Highlighting the role of Wnt signaling, inhibition of Wnt production by porcupine inhibitor IWP-2 reduced the branching of control organoids but had no further effects on *Lama5*-edited organoids (Fig. S5D). In order to model the phenotypes we observed in the mouse model more closely, and to assess the cell type-specific effect of Lama5 in organoid growth, we next isolated MECs from *Lama5^fl/fl^;K8-CreERT2* and control mice 3 days after tamoxifen treatment (Fig. 5F). Such preparations contain both luminal and basal cells, but *Lama5* loss is exclusive to luminal cells (Fig. S2B). Recapitulating results with lentiviral targeting, branching frequency was decreased in organoids with luminal-specific *Lama5* deletion, and exogenous Wnt4 rescued the branching defect (Fig. 5F,G). Moreover, branching was inhibited in control and *Lama5*-deficient organoids with the addition of IWP-2. These data further demonstrate that Lama5, specifically in luminal cells, is necessary for branching morphogenesis via paracrine signaling.

Targeting *Lama5* or the rescue of branching with exogenous Wnt ligands did not significantly change the frequency of Ki67^+^ cells (Fig. S5E), suggesting mechanisms other than proliferation. When stained for K8 and E-cadherin to visualize the luminal organoid structure, both lentivirally *Lama5*-edited and *Lama5^fl/fl^;K8-CreERT2* organoids showed similarly altered morphology with blunted branching (Fig. 5H; Fig. S5F). However, upon Wnt4 treatment both formed elongated branches that contained K14-expressing basal cells, and were structurally similar to branches in control organoids (Fig. 5H,I; Fig. S5F,G). Further supporting the role of Wnt signaling in promoting basal cell function during branching, exogenous Wnt3a and Rspo1 also resolved the structure of *Lama5*-deficient organoids (Fig. S5G).

Finally, to probe the possible interdependence of the *in vivo* observed phenotypes on gland growth and differentiation during pregnancy, we tested the lactogenic differentiation of organoids lacking luminal *Lama5*. MECs were isolated from *Lama5^fl/fl^;K8-CreERT2* and control mice 3 days after tamoxifen, and organoids were first grown in conditions inducing branching for 6 days (Fig. 5J). Next, differentiation was induced with lactogenic medium (LM) for an additional 6 days (Sumbal et al., 2020), leading to a robust increase in the milk protein genes in control organoids (Fig. S6A). Consistent with our *in vivo* results, loss of *Lama5* led to a decreased induction of Csn2 expression following the use of lactogenic medium (Fig. 5J; Fig. S6B). However, although Wnt4 further increased Csn2 expression in controls, and it rescued the branching defect of Lama5-deficient organoids as before, it did not rescue the defective lactogenic induction in Lama5-deficient organoids. Thus, although Wnt4 can resolve the defective organoid morphology in Lama5-deficient organoids, it is not sufficient to support the full functional differentiation of luminal epithelium lacking Lama5.

**Fig. 6.**
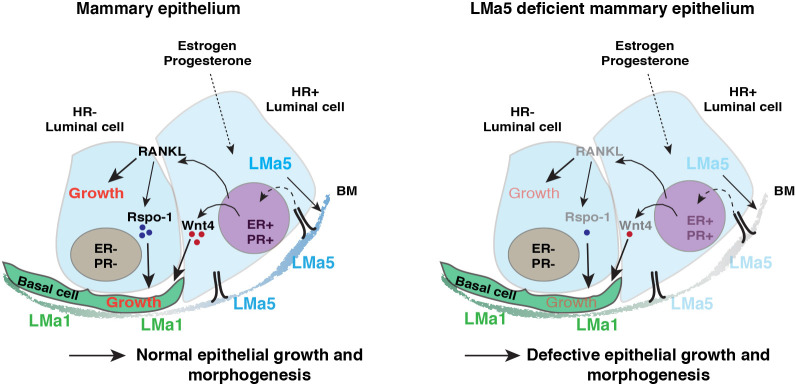
**Working model of how luminal Lama5 regulates mammary epithelial growth and crosstalk.** Schematic showing how the production of LMα5 by luminal MECs supports specifically the HR^+^ luminal cells, in which it enhances gene expression of ER, PR, Wnt4 and RANKL. The products of these genes then act on neighboring basal and HR^−^ epithelial cells to orchestrate epithelial growth and morphogenesis during puberty and pregnancy. Deficiency of LMα5 in the BM environment in turn leads to reduced gene expression in these genes, and defective intercellular communication and epithelial growth.

In conclusion, our data suggest that downstream of attenuated HR^+^ cell differentiation, loss of *Lama5* in the luminal epithelium results in defective gland growth due to altered interplay between basal and luminal epithelial cells mediated by Wnt, and in defective functionalization upon pregnancy (Fig. 6).

## DISCUSSION

The mammary ECM is essential for normal physiology and pathology of the mammary gland (LaBarge et al., 2009; Maller et al., 2010; Goddard et al., 2016). However, the exact functions of many specific BM constituents in mammary gland physiology have remained unclear. Here, we show that BM laminin subunits α1, α3, α4 and α5 exhibit distinct gene expression patterns in the luminal, basal and stromal compartments of the mammary gland. We discovered that *Lama5* is predominantly expressed in the HR^+^ luminal epithelial cells, and when *Lama5* was deleted in the luminal cells at various stages, we found that it has a critical role during pubertal growth and pregnancy-induced remodeling and growth. Our results are in concert with previous studies showing that the downregulation of laminin-binding adhesion receptors, including β1- and β4-integrins, results in the inhibition of mammary epithelial growth and alveologenesis (Li et al., 2005; Naylor et al., 2005; Walker et al., 2020; Romagnoli et al., 2020). Together, these data underline the importance of laminin and BM microenvironment in the regulation of mammary epithelial growth and differentiation.

The growth of the mammary epithelium occurs in a coordinated manner, requiring interplay between both luminal and basal cell types (Macias and Hinck, 2012; Inman et al., 2015). HR^+^ cells have been indicated as essential mediators of this interplay by sensing systemic hormones and producing paracrine signals for the neighboring HR^−^ luminal cells and the basal cells (Tanos et al., 2012). We observed a moderate reduction in the number of HR^+^ luminal epithelial cells in the Lama5-deficient mammary epithelium, but a more marked reduction in the HR^+^-specific expression of *Esr1*, *PR* and *RANKL*. Additionally, Sca1 marking both HR^+^ cells and their progenitors (Sleeman et al., 2007; Shehata et al., 2012), showed a decrease after *Lama5* loss. These data suggest qualitative changes in the *Lama5*-deficient HR^+^ luminal cells, which we postulate impairs their capability to sense and respond to systemic and paracrine signals. Previous studies have established Wnt signaling as an important mediator of the interactions between HR^+^ and basal epithelial cells to induce mammary epithelial growth (Zeng and Nusse, 2010; Rajaram et al., 2015; Cai et al., 2014). We show that *Lama5*-deficient luminal cells produce less Wnt4, which is expressed specifically by HR^+^ cells, and functions as a downstream effector of *Esr1* and *PR* (Cai et al., 2014; Rajaram et al., 2015). We also demonstrate decreased expression of Wnt target genes *Lgr5*, *Ctnnb1* and *Sox9* in the basal epithelial cells of *Lama5*-deficient epithelia. Moreover, in our *in vitro* studies, exogenous administration of Wnt4 rescued organoid branching defects caused by *Lama5* loss*.* Thus, we conclude that the altered luminal-basal interactions, which are mediated by Wnt signaling, can in part explain the defective growth and remodeling of *Lama5*-deficient glands.

Loss of Lama5 from the adult mammary epithelium prior to pregnancy led to reduced growth and defective differentiation of alveoli during pregnancy-induced functionalization. Correspondingly, downregulation of Lama5 *in vitro* led to defective lactogenic differentiation in organoid cultures. Intriguingly, in these settings, the addition of Wnt4 was unable to rescue the differentiation defect, suggesting a lack of other essential factors. Expansion of luminal epithelial cells during pregnancy is largely attributable to HR^−^ epithelial cells (Van Keymeulen et al., 2017), presumably mostly in response to progesterone-mediated signals from HR^+^ cells (Brisken et al., 1998). Therefore, it is interesting to hypothesize that the paracrine signaling between HR^−^ and HR^+^ cells is also impaired in Lama5 lacking epithelium. Indeed, we observed that RANKL, which is expressed by the HR^+^ cells downstream of progesterone (Fata et al., 2000; Asselin-Labat et al., 2010), was significantly downregulated in the Lama5-deficient epithelial cells. Furthermore, loss of RANKL has been shown to inhibit alveologenesis and lactogenic differentiation (Fata et al., 2000), which could underlie the *Lama5*-dependent deficiency in lactogenic differentiation observed *in vitro*. However, the small number of HR^+^ cells shown to be present in the pregnant mammary gland (Van Keymeulen et al., 2017) could in part explain the moderate phenotype observed in pregnant glands.

*Lama5* deficiency also affected the ductal epithelial organization in puberty and pregnancy. Our results imply that the Lama5-deficient luminal cells proliferate, albeit less than cells in control glands, but fail to maintain normal contacts with the BM. This may result in the observed accumulation of cells filling the gland lumen. Luminal-ECM interactions are necessary for alveolar differentiation and, thus, functionalization and milk secretion (Inman et al., 2015; Streuli et al., 1995; Roskelley et al., 1994). Indeed, mammary epithelia lacking the adhesion receptor β1-integrin exhibit defective apicobasal polarization, surplus luminal cells within the ducts and impaired alveolar cell function (Naylor et al., 2005; Akhtar and Streuli, 2013). We noted that apicobasal polarity is affected also in the *Lama5*-deficient luminal epithelial cells, suggesting it may contribute to the surplus of luminal cells during puberty. Cell polarity was better conserved in the *Lama5*-deficient glands during pregnancy, suggesting the most aberrant glands may not be able to contribute to pregnancy-induced gland remodeling. However, addressing the exact order of events downstream of *Lama5* deletion and loss of tissue architecture during puberty requires further experiments.

Finally, how adhesion to *Lama5*-encoded Lama5 regulates Wnt4 signaling, and why particularly HR^+^ cells are influenced by *Lama5* loss remain as open questions. As HR^+^ cells express more Lama5 than HR^−^ luminal cells (Tabula Muris Consortium, 2018), and HR^+^ are responsive to systemic cues, it is attractive to postulate that they require Lama5-mediated contacts with the BM in order to sense the systemic hormonal signals. HR^+^ luminal cells also express, for example, Integrin b4 (*Itgb4*) capable of adhering to Lama5 (Yurchenco, 2011), whereas *Itgb4* expression in HR^−^ cells is lower (Tabula Muris Consortium, 2018). The slender projections of luminal cells that we noted reaching past basal cells may therefore represent an HR^+^ cell-specific feature of the luminal layer.

Taken together, our data suggest that *Lama5* loss in the mammary epithelium results in adhesion and organization defects of luminal MECs, leads to a deficient HR^+^ luminal MEC pool with insufficient capability to sustain luminal-basal interactions, and gland remodeling in puberty and pregnancy. Further experiments are required to address whether additional functions of Lama5 partake mammary gland biology *in vivo* and whether the mechanisms described here contribute to pathologies with distorted tissue organization, such as cancer.

## MATERIALS AND METHODS

### Animals

Animal studies were approved by the National Animal Ethics Committee of Finland and conducted in the Laboratory Animal Center of the University of Helsinki under institutional guidelines or approved by the ethics committee of the Board of Agriculture, Experimental Animal Authority, Stockholm, Sweden. The *Lama5 fl* mouse was a kind gift from Dr Jeffrey Miner (Nguyen et al., 2005). *Lgr6*-*EGFP*-*IRES-Cre^ERT2^*, *Rosa26-tdTomato*, *K8-CreERT2* and *R26-mTmG* mouse lines have been described previously (Muzumdar et al., 2007; Van Keymeulen et al., 2011; Nguyen et al., 2005; Blaas et al., 2016). Genotyping of the animals was performed using primers described previously. Excised *Lama5* allele was detected using the following primers: Fwd, 5′-ACCTGGCTTTGACGGTCCT-3′; Rev, 5′-GTTGAAGCCAAAGCGTACAGCG-3′. All animals used were females between 6 and 20 weeks of age at the time of sacrifice. For Lgr6-Cre induction, a single dose of 1 mg tamoxifen (Sigma-Aldrich) in 50 µl sunflower oil (Sigma-Aldrich) was injected intraperitoneally in 2-week-old mice. For K8-CreERT2 induction, a single dose of 1.5-2 mg tamoxifen in corn oil (Sigma-Aldrich) was injected intraperitoneally in 3-week-old mice, and 5 mg of tamoxifen in 8-week-old mice.

### RNA *in situ* hybridization

Gene expression analysis by RNA ISH was performed using an RNAscope 2.5 HD Reagent Kit-Brown or an RNAscope Multiplex Kit (Advanced Cell Diagnostics Srl) according to manufacturer's instructions. Paraffin-embedded sections (5 µm) were deparaffinized, rehydrated and pretreated with RNAscope Target Retrieval Reagents. A barrier was created around each tissue section on the slides, dried overnight and used the following day. Pretreated samples were hybridized with either laminin α1, α3, α4 or α5 probes (custom made by Advanced Cell Diagnostics) for 2 h at 40°C. Thereafter, signal amplification hybridization was performed, followed by detection with DAB (3,3′-diaminobenzidine) and counterstained with Hematoxylin. Alternatively, when a Multiplex Fluorescent assay was used, after signal amplification hybridization, the slides were immunostained with primary antibodies (K8, 1:1000, TROMA-1, Developmental Studies Hybridoma Bank; K14, 1:2000, BioLegend, PRB 155P) for 1 h. Thereafter, samples were washed three times with PBS, 5 min each, and incubated with secondary antibodies in 10% normal goat serum in immunofluorescence buffer for 1-2 h. Slides were next washed three times, followed by counterstaining of nuclei with DAPI and mounting. Samples were imaged using a Zeiss Axio Imager.M2 and AxioCam HRc camera using Plan Neofluar 20× (NA 0.5) or Plan Apochrom 63× (NA 1.4, oil) objectives and Zen software, or using a Nikon Inverted widefield system with a 60× Plan apochromat objective (NA 1.2, water) and Andor Xyla 4.2+ camera, and NIS elements software (Nikon).

### Whole-mount stainings

Mammary gland tissue samples for whole-mount staining were fixed in 4% paraformaldehyde (PFA) overnight. For whole-mount staining, #4 inguinal mammary glands were stained for several hours in carmine-alumn staining solution (2% w/v Carmine, Sigma-Aldrich; 5% w/v aluminum potassium sulfate, Sigma-Aldrich). After the desired color had developed, glands were mounted to glass coverslips and imaged.

For whole-mount imaging and immunostaining of *Lgr6-CreERT2;Rosa26-tdTomato* or *Lama5 fl;K8-CreERT2;Rosa26-mTmG* mammary glands, the dissected mammary glands were pre-fixed in 4% PFA for 30–60 min at room temperature to preserve native fluorescence. For whole-mount immunostaining, the tissue was permeabilized with two washes of PBS0.5T (PBS plus 0.5% v/v Triton X-100) for 1 h, and the samples were incubated overnight at 4°C with primary antibody against keratin 14 (1:500, BioLegend PRB 155P) diluted in PBD0.2T [PBS, 1% bovine serum albumin (BSA), 1% v/v DMSO and 0.2% v/v Triton X-100]. The next day, they were washed four times with PBD0.2T for 1 h. Tissues were incubated with secondary antibodies plus DAPI (Roche) at 4°C overnight. After four washes with PBD0.2T for 1 h, tissues were optically cleared in 80% glycerol in PBS at room temperature. The stained mammary glands were mounted between two cover slips and sealed with silicone. Imaging of the whole mounts was performed using a Zeiss LSM710 confocal microscope. A dry lens (5×, EC Plan Neofluar, NA 0.16) or a water immersion lens (20× W Plan Apochromat NA 1.0 or 40× C-Apochromat, NA 1.2) was used to record optical sections at 512×512, 1024×1024 or 2048×2048 pixels, and data were processed using ZEN software (Zeiss). Images were converted to RGB images with ImageJ software.

### Morphometric analysis of the mammary gland

For quantification of the number of terminal end buds (TEB) and ductal ends in the mammary glands and the length of the epithelium, #4 mammary glands stained with carmine-alumn whole mount were imaged using a Leica S9i stereomicroscope equipped with an integrated 10 MP camera. The amount of TEBs and ductal ends was quantified from the images based on morphology. The length of the epithelium was measured from the same images using ImageJ with its line tool. The length of the whole epithelial network was recorded.

For quantification of ductal density, images of Hematoxylin and Eosin (H&E)-stained pregnant mammary glands were acquired using a Leica Microscope DM2000 with a Leica MC 190 HD camera with an N Plan 10× objective. The percentage of ductal density was quantified from 10× images by applying a series of Gaussian filtering and thresholding for brightness to separate stained tissue area from the lumen and surrounding adipose tissue using a custom Java script that can be obtained from the authors. The ratio of the red channel to the other channels, balanced with the brightness, was used to further separate Hematoxylin-stained nucleus-rich ductal tissue from Eosin-stained connective tissue, and produce a binary image. A size-based filter was applied to remove single nuclei and debris. The remaining area representing the ducts was calculated for each tissue slide.

### Tissue immunostainings

Mammary gland tissue samples for immunostaining were fixed in 4% PFA overnight and embedded in paraffin for sections. For H&E stainings, 5 µm sections were used. Stainings were performed in the Finnish Center for Laboratory Animal Pathology (FCLAP) at the University of Helsinki. Imaging for representative images was performed using a Pannoramic 250 Flash II high-throughput brightfield scanner (3DHISTECH) with a 20× (NA 0.8) objective and Pannoramic Viewer software.

For immunofluorescence stainings, 6-8 µm sections were deparaffinized and rehydrated, and antigen retrieval was performed with 1 mM EDTA for 5-8 min in a microwave oven, followed by 20 min incubation at room temperature. Thereafter, non-specific binding sites were blocked with 10% normal goat serum (NGS, Gibco) in PBS for 30 min at room temperature. Next, samples were incubated with primary antibodies (pan-laminin 1:500, Abcam, ab11575; K14, 1:500, BioLegend, PRB 155P; K8, 1:1000, TROMA-1, Developmental Studies Hybridoma Bank; Ki-67, 1:500, Abcam, ab15580; E-cadherin 36/E, 1:500, BD 610181; ZO-1, 1:500, Thermo Fisher Scientific, 61-7300; and MUC, 1:500, Abcam, ab15481) overnight at 4°C. The next day, samples were washed three times with PBS, 5 min each, and incubated with secondary antibodies in 10% NGS in PBS for 1-2 h. Thereafter, samples were washed three times with PBS, 5 min each, followed by counterstaining of nuclei with Hoechst 33342 (Sigma-Aldrich). All slides were mounted using Immu-Mount (Thermo Scientific) mounting reagent. Images were acquired using a Leica TCS SP8 STED 3X CW 3D confocal with an HC PL APO 63× water (NA 1.20) motCORR CS2 objective and LAS AF software, or a Leica SP8 upright confocal microscope with an HC PL APO 63× glycerol (NA 1.3) objective and LAS AF software. The same settings for laser power and detector gain were maintained within one experiment.

### Quantification of fluorescence in mammary gland whole mounts

Mammary gland whole mounts of *Lama5^+/+^;K8-CreERT2;Rosa26-mTmG* and *Lama5^fl/fl^;K8-CreERT2;Rosa26-mTmG* were imaged for 3-5 proximal and distal areas of the mammary epithelium. *Z*-stacks were converted to *Z* projections using the sum slice method. Regions of interest (ROI) were drawn to encompass the epithelium and three spots were drawn to quantify the background fluorescence. The integrated density of green and red channels of ROIs was measured, and mean background fluorescence was subtracted from the epithelial ROIs. From these values, the green to red fluorescent ratio was calculated for both proximal and distal areas. Data are shown as mean per mammary gland.

### FACS analysis and sorting of primary MMECs

Single-cell suspension of isolated primary mouse mammary epithelial cells (MMECs) was resuspended in 0.2% BSA in Dulbecco's PBS, and the cells were incubated with the following primary antibodies for analysis: CD29-FITC (Miltenyi Biotech, 130-102-975); CD24-APC (M1/69, BioLegend, 101814); CD31-BV421 (MEC13.3, BD Biosciences, 562939); CD45-BV421 (30 F11, BD Biosciences, 563890); Ter119-BV421 (BD Biosciences, 563998); Sca1-BV711 (D7, BioLegend, 108131); and CD49b-PE (HMα2, BioLegend, 103506). All antibodies were diluted 1:500 at 4°C for 30 min. Cells were washed with PBS and resuspended in 0.2% BSA in Dulbecco's PBS with 1:500 Sytox Blue (Life Technologies, Thermo Fisher Scientific) to exclude dead cells. Sorting was performed using a FACSAria Fusion cell sorter (Becton Dickinson). FlowJo V10 was used for post-analysis of sorted cells.

### RNA isolation and qPCR

RNA isolation was performed using an RNeasy isolation kit (Qiagen) combined with On-Column DNase digestion (Qiagen), or using TRIzol (Life Technologies, Thermo Fisher Scientific; from FACS-sorted cells) according to manufacturer's instructions. TRIzol-isolated RNA was treated with DNase I (Thermo Fisher Scientific) according to manufacturer's protocol. cDNA synthesis was performed using a Revert Aid cDNA synthesis kit (Thermo Fisher Scientific) starting with 250 or 500 ng of RNA. Quantitative PCR reaction was performed using Power SYBR green master mix (Applied Biosystems) and a Bio-Rad CFX384 Touch Real-Time PCR detection system. Data were analyzed using a Bio-Rad CFX Manager program. Relative mRNA amounts were assayed by comparing PCR cycles to GAPDH using the ddCT method and normalizing to control samples. The following primers were used: mGAPDH fwd, 5′-AAGGTCGGAGTCAACGGATT-3′; mGAPDH rev, 5′-TTGATGACAAGCTTCCCGTT-3′; mLama5 WT fwd, 5′-GGGTGGAGTTACTGAGTGCC-3′; mLama5 WT rev, 5′-AGCTACGGCAGCCAAAGTAG-3′; mLama5 Common fwd, 5′-GCCAGCAAGGCGATCCAAG-3′; mLama5 Common rev, 5′-CCTGGTCTACGCTGAACACA-3′; mESR1 fwd, 5′-CCTCCCGCCTTCTACAGGT-3′; mESR1 rev, 5′-CACACGGCACAGTAGCGAG-3′ (Harvard Primer Bank ID, 6679695a1); mPR fwd, 5′-CTCGGACGTGTCGTCTGTAG-3′; mPR rev, 5′-CCTGTCTTTCCGTCTGGGAG-3′ (Harvard Primer Bank ID, 112363097c1); mRANKL fwd, 5′-CGCTCTGTTCCTGTACTTTCG-3′; mRANKL rev, 5′-GAGTCCTGCAAATCTGCGTT-3′ (Harvard Primer Bank ID, 114842414c2); mWnt4 fwd, 5′-GTACCTGGCCAAGCTGTCAT-3′; mWnt4 rev, 5′-CTTGTCACTGCAAAGGCCAC-3′; mRspo1 fwd, 5-TTCTGCTGGAGAGGAACGAC-3′; mRspo1 rev, 5′-GCCTCACAGTGCTCGATCTT-3′; mLgr5 fwd, 5′-ACCCGCCAGTCTCCTACATC-3′; mLgr5 rev, 5′-GCATCTAGGCGCAGGGATTG-3′; mSox9 fwd, 5′-GAGCCGGATCTGAAGAGGGA-3′; mSox9 rev, 5′-GCTTGACGTGTGGCTTGTTC-3′; mCtnnb1 fwd, 5′-TGACTAGGGCTCAGAGGGTC-3′; and mCtnnb1 rev, 5′-TCAGCTCAGGAATTGCACGT-3′.

### Primary cell isolation and culture

Primary MMECs were isolated from 10-16-week-old virgin female mice, unless otherwise stated. Mammary glands #3-#5 were dissected, and the lymph node in #4 glands was removed and the glands were finely chopped. Tissue was incubated with 0.01 mg of Collagenase A (Sigma-Aldrich) per 1 g of tissue in Dulbecco's modified Eagle medium (DMEM)/F12 growth medium (Life Technologies) containing 2.5% fetal calf serum (FCS), 5 µg/ml insulin, 50 µg/ml gentamicin and 2 mM glutamine with gentle shaking (120 rpm in an environmental shaker) at 37°C for 2 h. The resulting cell suspension was then first centrifuged at 400 ***g*** for 10 min and consecutively pulse-centrifuged 3-5 times 400 ***g*** to yield preparation free of cells other than MMEC organoids. Next, organoids were trypsinized with 0.05% Trypsin-EDTA (Difco, J.T. Baker) for 5-10 min to obtain smaller organoid units and drained through a 70 µm cell strainer (Becton Dickinson), and resuspended in MMEC growth medium [DMEM/F12 medium containing 5 µg/ml insulin, 1 µg/ml hydrocortisone, 10 ng/ml mouse EGF, 2 mM glutamine, 50 µg/ml gentamycin and penicillin and streptomycin (all from Sigma-Aldrich) supplemented with 10% FCS (Gibco)].

### Lentiviral virus production and infection

Lentiviruses were produced in 293fT cells (Thermo Fisher Scientific; regularly tested for mycoplasma contamination) grown in DMEM (Sigma-Aldrich) supplemented with 10% FCS (Gibco), Penicillin/Streptomycin (Orion/Sigma-Aldrich) and 2 mM glutamine (Sigma-Aldrich). Transfections of transfer vector (pLentiCRISPRv2 gSCRA, pLentiCRISPRv2 gLAMA5) and packaging plasmids (CMV-VSVg and Delta8.9) were performed using Lipofectamine 2000 (Invitrogen) according to the manufacturer's instructions. To concentrate lentiviral particles, medium from transfected cells was harvested 72 h post-transfection and concentrated by ultracentrifugation at 87,000 ***g*** for 120 min (Sorvall Discovery 90SE Ultracentrifuge). The viral pellet was then resuspended in PBS at a ∼300-fold concentration and left to dissolve at 4°C for 18 h. Viral titer was determined using a p24 ELISA test measuring viral capsid protein p24 (Perkin Elmer, performed in the Biomedicum Functional Genomics Unit). Primary MMECs were infected on 24-well low adhesion plates overnight using multiplicity of infection 5 in an 800 µl volume and washed with growth medium the next day.

### Plasmid construction for CRISPR guides

CRISPR guides were designed to target LAMA5 using the CRISPR Design tool (http://crispr.mit.edu). The following target sequences were used: gLAMA5, 5′-CCATCGATGGCACGGAGCGC-3′, and gSCRA, 5′-CTAAAACTGCGGATACAATC-3′. Oligos with target sequences were designed according to instructions published previously (Shalem et al., 2014), annealed and cloned into the pLentiCRISPRv2 vector.

### Three-dimensional organotypic cell culture assays

Three-dimensional organotypic culture was performed in growth factor-reduced BM from Engelbreth–Holm–Swarm mouse sarcoma (Matrigel, Becton Dickinson), which was prepared according to the manufacturer's instructions. Isolated primary MMECs grown on low adhesion plates were trypsinized with 0.05% Trypsin-EDTA for 5-10 min, centrifuged and suspended with liquid Matrigel, and plated onto 8-well chamber slides at ∼1500 cells/well. Organoids were grown in DMEM/F12 supplemented with ITS medium supplement (Sigma-Aldrich), penicillin and streptomycin and 2.5 nM FGF-2 (Sigma-Aldrich). Wnt3a and Wnt4 (both R&D Biosystems) were used in 100 ng/ml, Rspo1 (R&D Biosystems) in 500 ng/ml, and porcupine inhibitor IWP-2 (Sigma-Aldrich) in 2 µM, and were added on the starting day of the cultures. Medium was refreshed every 3-4 days. For lactogenic differentiation, organoids were first grown for 6 days in the DMEM/F12 medium supplemented with 2.5 nM FGF-2, ITS and penicillin and streptomycin, and thereafter, 6 days in the same culture medium additionally supplemented with 1 μg/ml mouse prolactin (Sigma-Aldrich) and 1 μg/ml hydrocortisone (Sigma-Aldrich).

### Three-dimensional organoid immunofluorescence staining and imaging

Three-dimensional organoids were fixed with 2% PFA for 20 min at room temperature and thereafter washed with PBS. Organoids were permeabilized with 0.25% Triton X-100 in PBS for 10 min at 4°C, and thereafter washed with PBS. The non-specific binding sites were blocked with 10% normal goat serum (Gibco) for 1-2 h. The primary antibodies (keratin 14, Covance, 1:300; Keratin 8, TROMA-1, 1:300; Ki-67, Abcam, 1:500) were incubated in the blocking solution overnight at 4°C. Following the incubation, structures were washed three times with PBS, 15 min each wash, and then incubated with appropriate Alexa Fluor secondary antibody diluted in blocking solution. After 40-50 min incubation at room temperature, the structures were washed with IF buffer as before and the nuclei were counterstained with Hoechst 33342. All slides were mounted using Immu-Mount (Thermo Scientific) mounting reagent. Images were acquired using a Leica TCS SP8 STED 3X CW 3D confocal microscope with an HC PL APO 63× water (NA 1.20) motCORR CS2 objective and LAS AF software, or a Leica SP8 Upright confocal microscope with an HC PL APO 63× glycerol (NA 1.3) objective.

### Quantification and statistical analysis

Data are presented as mean±s.d. from at least three independent experiments or three individual animals quantified, unless otherwise stated in the figure legend. An unpaired two-tailed Student's *t*-test was used to compare two groups, unless otherwise stated in the figure legend. Statistical tests were performed using GraphPad Prism 8.

## Supplementary Material

Supplementary information

Reviewer comments
