# Supplementary material for: Laminin alpha 5 regulates mammary gland remodeling through luminal cell differentiation and Wnt4-mediated epithelial crosstalk
Source: Development. 2021 Jun 15;148(12):dev199281. doi: 10.1242/dev.199281 (PMC8254867; doi:10.1242/dev.199281)
Supplement: Supplementary information [file develop-148-199281-s1.pdf]

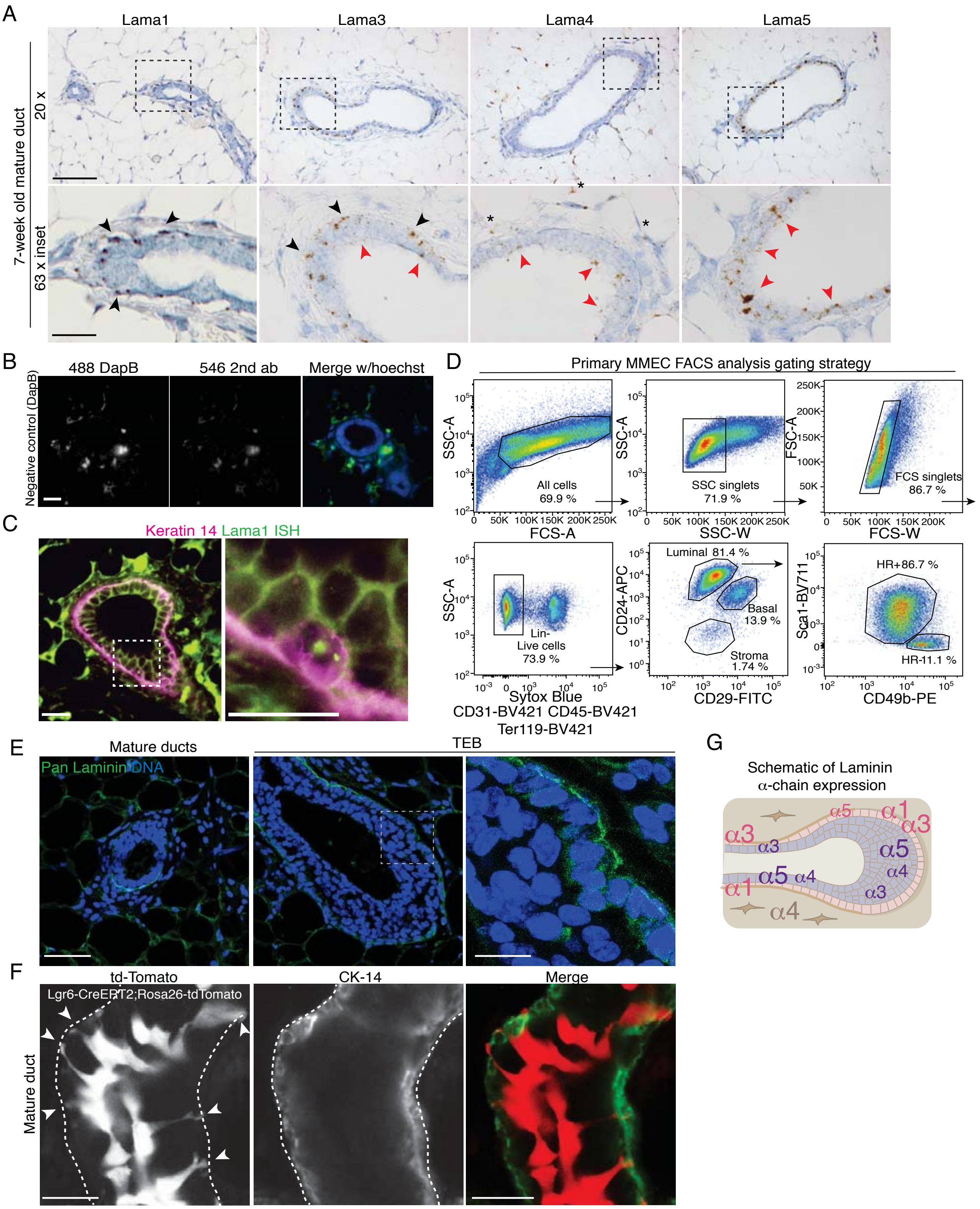

Figure S1. Englund JI et al. (related to Figure 1) Laminin α chain expression in the mammary gland.

(A) RNA in situ hybridization performed with probes for Lama1, 3, 4 and 5 on mature ducts of 7-week old pubertal mammary glands. Expression by basal or luminal cells is indicated by black and red arrowheads, respectively. Black asterisks mark expression in stromal cells. Scale bars 50 μm, 20 μm (insets). (B) Representative image of in situ hybridization performed with negative control probe DapB conjugated with 488 and alexa-546 conjugated secondary antibody showing fluoresncent background staining. Scale bar 20 μm. (C) Representative images of Lama1 RNA ISH and K14 antibody staining. Scale bars 20 μm (D) Representative FACS plots and gating strategy for separation of stromal cells, basal and luminal primary MMECs and HR- and HR+ luminal subpopulations in the mouse mammary gland. (E) Immunofluorescence images of 7-week old mature mammary ducts and TEBs with Pan-Laminin antibody. Scale bar 50 μm. (F) A representative image of luminal cells in tamoxifen treated 4-week old Lgr6-CreERT2;Rosa26-tdTomato mammary epithelium immunostained to visualize Keratin 14. Dotted line marks the interface between BM and the epithelium. Note the extensions by bright tdTomato positive luminal cells to the BM (white arrowheads). Scale bars 20 μm. (G) Schematic showing Laminin α-chain expression in the TEB and mature ducts of mouse mammary gland. Laminin α-chains expressed in the basal cell layer (pink) are depicted in light red and α-chains expressed in the luminal layer (light blue) are depicted in violet. Stromally expressed laminins are depicted in brown.

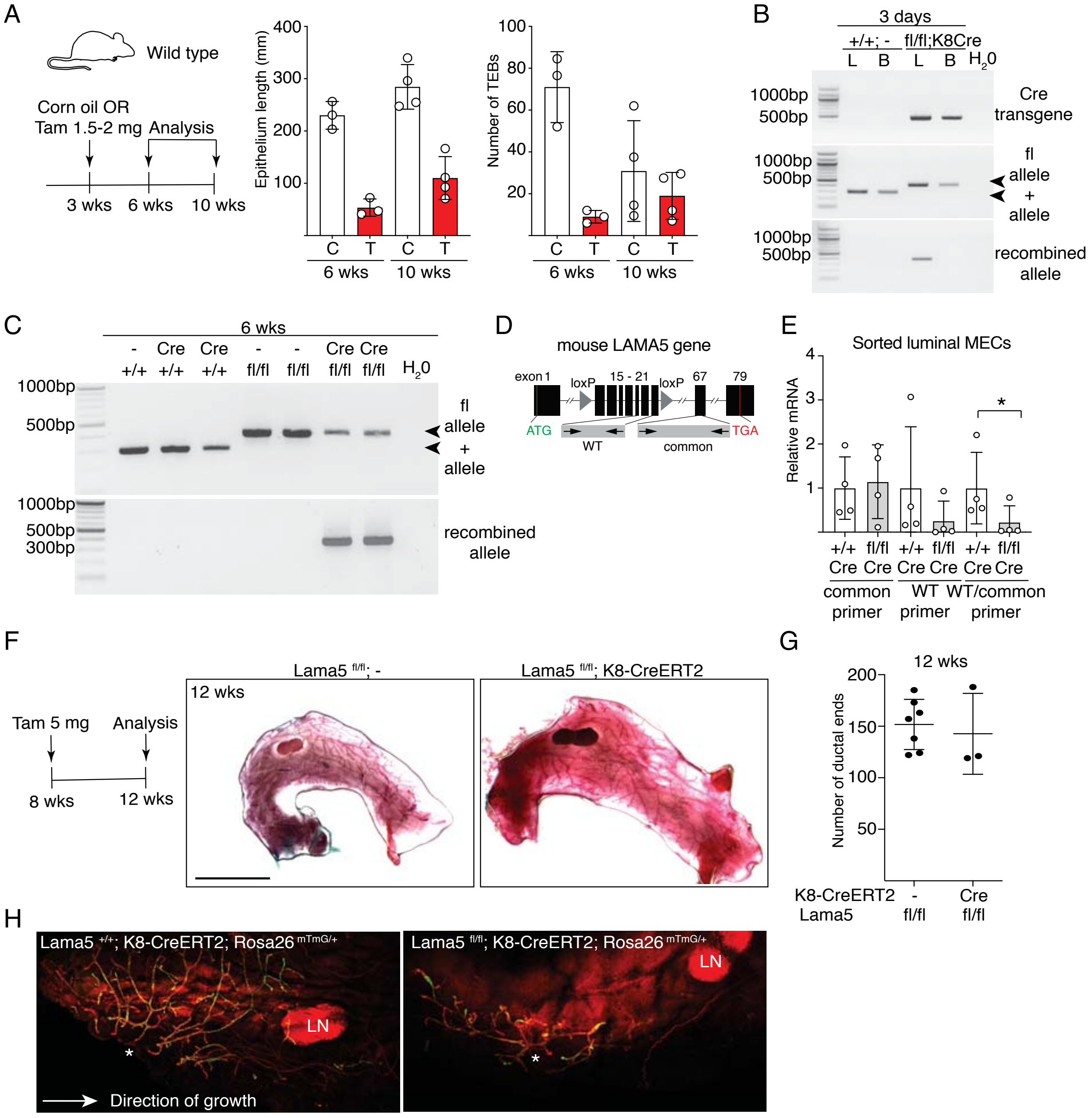

Figure S2. Englund JI et al. (related to Figure 2) Laminin α5 is required for mammary epithelial growth

(A) Schematic showing the outline of corn oil or tamoxifen treatment during puberty. #4 mammary glands of wild type female mice were analyzed at 6 weeks and 10 weeks for total epithelial length and TEB number in mice treated with corn oil (C) or tamoxifen (T). (B) Genomic PCR of DNA isolated from luminal (L) and basal (B) MECs of 8-week old Lama5$^{+/+}$;- and Lama5$^{fl/fl}$;K8-CreERT2 mice treated with tamoxifen for 3 days. Uppermost image shows PCR product of K8-Cre transgene (450 bp), middle image Lama5$^{+/+}$ (296 bp) and Lama5$^{fl/fl}$ allele (460 bp) and lower image of recombined Lama5^{fl} allele (250 bp, predicted). (C) Genomic PCR of DNA isolated from mammary glands of 6-week old mice treated with tamoxifen at 3-weeks of age. Upper image shows PCR products of Lama5$^{+/+}$ (296 bp) or Lama5$^{fl/fl}$ allele (460 bp) and lower gel of recombined Lama5^{fl} allele (250 bp, predicted). (D) Quantitative PCR strategy to analyse Lama5 mRNA expression in Lama5$^{fl/fl}$ and Lama5$^{+/+}$ animals. Common primer pair binds exon 67, and WT primer pair exons 19 and 20/21 (Ex19-21), which reside in the area between LoxP sites targeted for recombination. (E) Quantitative PCR analysis of Lama5 expression in pubertal sorted luminal MECs. Common primer is expressed at equal levels in sorted luminal MECs from Lama5$^{fl/fl}$ and control animals, while WT primer expression is decreased in Lama5$^{fl/fl}$ luminal MECs compared to GAPDH. Rightmost bars show normalization of WT primer to common primer, shown also in Fig 2a. Two-tailed student's t-test was used to compare indicated groups. (F) Schematic showing the outline of tamoxifen treatment, and representative images of carmine-alumn stained wholemounts of #4 mammary glands of 12-week old Lama5$^{fl/fl}$;- and Lama5$^{fl/fl}$;K8-CreERT2 mice. Scale bar 10 mm. (G) Quantification of the number of ductal ends in 12-week old transgenic mice treated with tamoxifen at 8-weeks of age. (H) Representative images of whole #4 mammary glands of 6-week old Lama5$^{+/+}$;K8-CreERT2;R26$^{mTmG/+}$ and Lama5$^{fl/fl}$;K8-CreERT2 R26$^{mTmG/+}$ mice. Asterisk marks the beginning of the ductal network, LN marks lymph node.

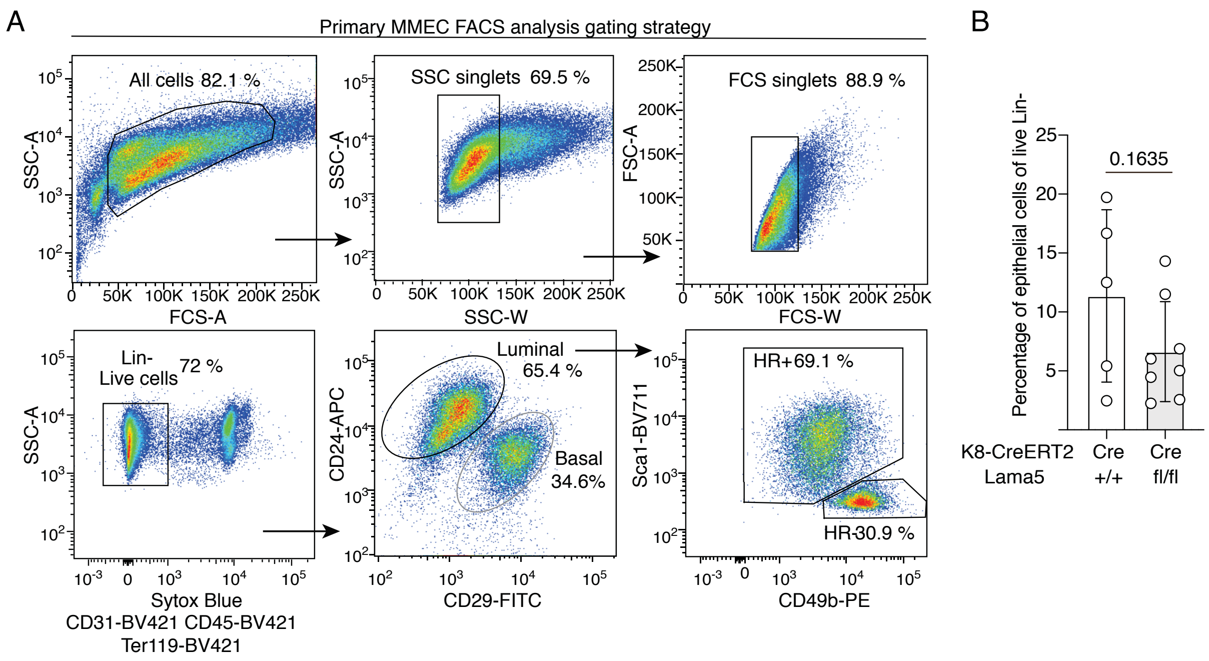

Figure S3. Englund JI et al. (related to Figure 3) FACS gating strategy and analysis of primary MMECs

(A) Representative FACS plots and gating strategy for separation of basal and luminal primary MMECs and HR- and HR+ luminal subpopulations from Lama5$^{+/+}$;K8-CreERT2 and Lama5$^{fl/fl}$;K8-CreERT2 mice. Same CD24/CD29 plot from Lama5$^{+/+}$;K8-CreERT2 control animal with luminal and basal sorting gates is shown in Figure 3a. (B) Graph showing the prercentage of all epithelial cells in lineage negative (Lin-) live cell population in 8-10-week old Lama5$^{+/+}$;K8-CreERT2 and Lama5$^{fl/fl}$;K8-CreERT2 mice. Data show mean ± SD. Two-tailed student's t-test was used to compare indicated groups.

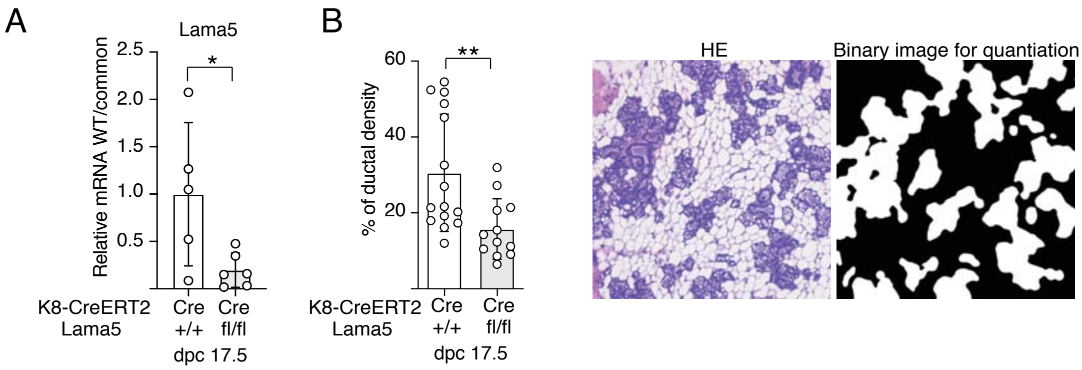

Figure S4. Englund JI et al. (related to Figure 4) Laminin α5 is essential for pregnancy-induced growth

(A) Quantitative PCR analysis of Lama5 expression luminal MECs FACS sorted from dpc 17.5 pregnant mice**.** Same qPCR strategy as in Figure S2c is used. Lama5 mRNA expression in luminal MECs is analysed by using wild type (WT) allele specific primers compared to primers common for both + and flox alleles (common) Two-tailed student's t-test was used to compare indicated groups. (B) Quantitation of percentage of ductal area in mammary glands of dpc 17.5 pregnanc mice. Graph shows percentage of ductal density from 6 +/+;Cre and 5 fl/fl;Cre individual animals with each dot representing one image analysed. Two-tailed student's t-test was used to compare the groups as whole. Rigth panel show representative original HE image and the corresponding binary image used for quantitation of ductal density (white areas).

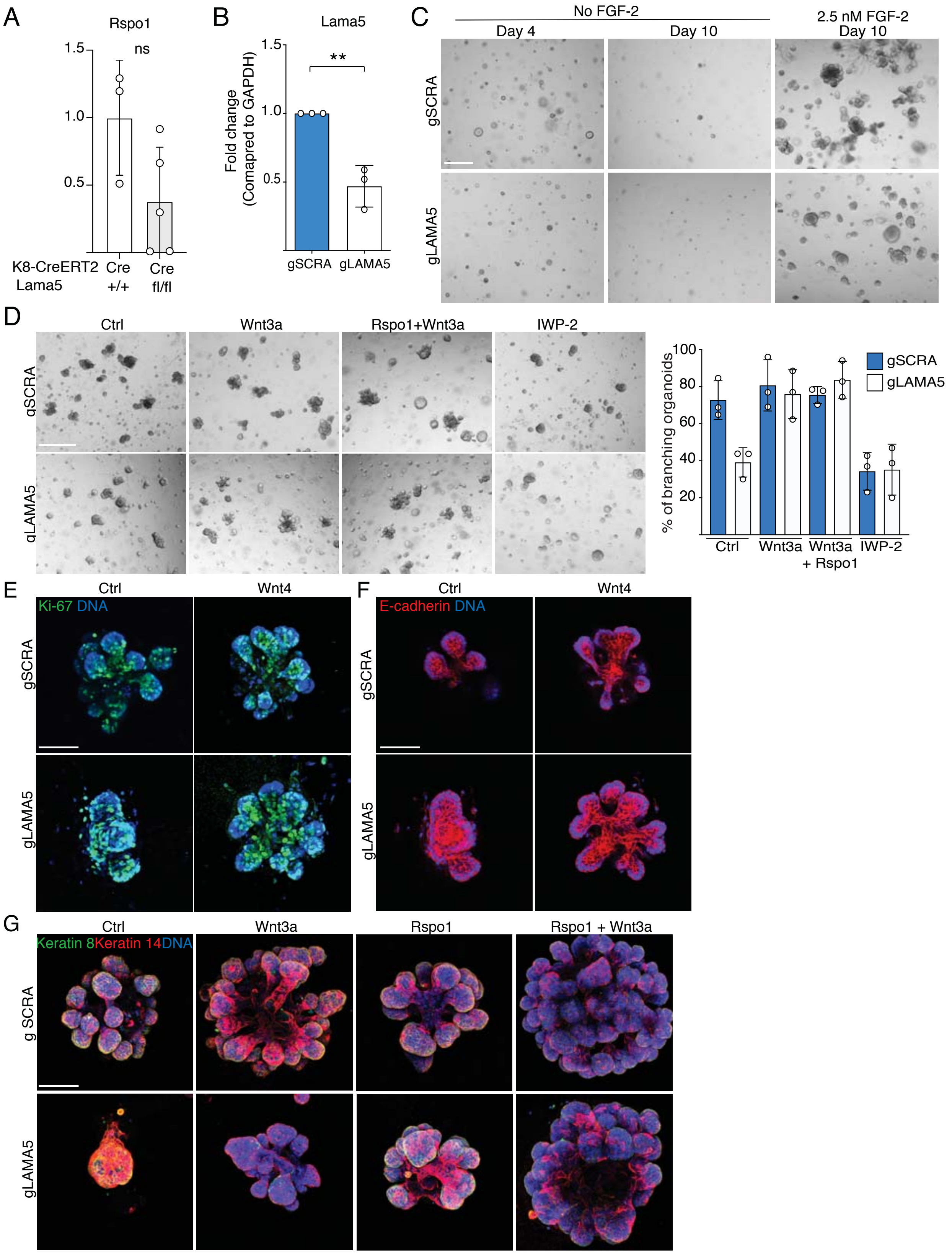

Figure S5. Englund JI et al. (related to Figure 5) Lama5 deleted luminal MECs exhibit defective Wnt signaling

(A) qRT-PCR analysis of Rspo1 expression compared to GAPDH in luminal ($CD29^{low}$/$CD24^{+}$) MECs FACS sorted from 8-10-week old $Lama5^{+/+}$;K8-CreERT2 and $Lama5^{fl/fl}$;K8-CreERT2 glands treated with tamoxifen starting from 3 weeks of age. Data show mean ±SD. N=3-5 independent samples per group. Two-tailed student's t-test was used to compare indicated groups. (B) qRT-PCR analysis of Lama5 mRNA expression in primary MMECs infected with lentiviruses carrying gLAMA5 or gSCRA CRISPR guides compared to GAPDH. N=3 individual experiments. Each biological replicate is compared to the gSCRA sample. Two-tailed student's t-test was used to compare indicated groups. (C) Primary MMECs carrying either gLAMA5 or gSCRA CRISPR guides grown in Matrigel in the presence or absence of 2.5 nM FGF-2 for 10 days. Scale bar 200 μm (D) Representative images of primary MMECs carrying either gLAMA5 or gSCRA CRISPR guides grown in Matrigel in the presence of 2.5 nM FGF-2 and either with Wnt3a, Rspo1 and Wnt3a or IWP-2 for 7 days (scale bar 200 μm) and quantification of percentage of branching organoids. N=3 individual experiments. Two-tailed student's t-test was used to compare indicated groups. (E) Representative immunofluorescence images of gLAMA5 or gSCRA MMECs grown as in B and stained for Ki-67. Scale bar 50 μm. (F) Immunofluorescence images of same gLAMA5 or gSCRA MMECs organoids as shown in d and stained for E-cadfherin. Middle part of z-stack is shown to illustrate organoid morphology. Scale bar 50 μm. (G) Representative immunofluorescence images of gLAMA5 or gSCRA CRISPR MMECs and as in b and stained for K8 and K14. Scale bar 100 μm.

Development • Supplementary information

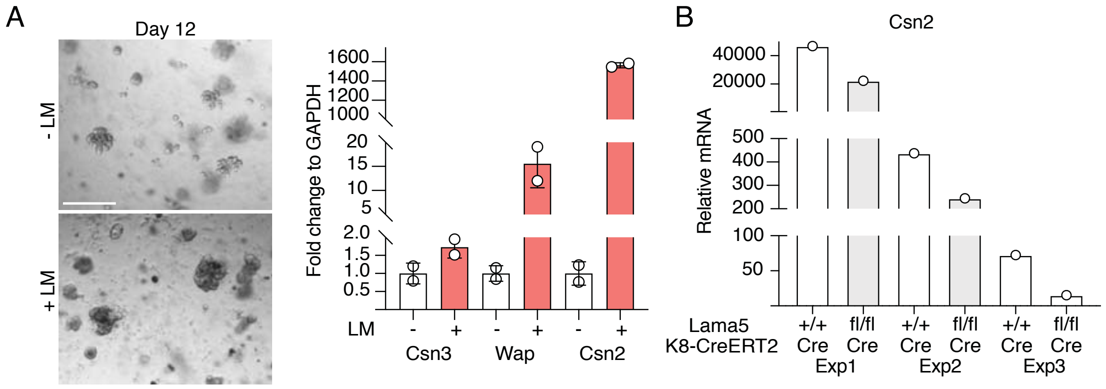

Figure S6. Englund JI et al. (related to Figure 5) Lama5 deficiency impairs lactogenic differentiation in vitro

(A) Representative phase contrast images of wild type MEC organoids treated with or without lactogenic media (LM) as described in the schematic in 5J, and quantitative PCR analysis of milk protein Csn3, Wap and Csn2 gene expression of from organoids collected at day 12 in two independent experiments. (B) Quantitative PCR analysis of Csn2 gene expression in MECs isolated from 10-14 week old Lama5$^{+/+}$;K8-CreERT2 and Lama5$^{fl/fl}$;K8-CreERT2 mice treated with tamoxifen 3 days earlier, and grown as described in 6A in LM. Each bar represents a single data point from indicated separate experiments (Exp1,2 or 3). Data is normalized to uninduced genotype control from each experiment.
