# Supplementary material for: Laminin alpha 5 regulates mammary gland remodeling through luminal cell differentiation and Wnt4-mediated epithelial crosstalk
Source: Development. 2021 Jun 15;148(12):dev199281. doi: 10.1242/dev.199281 (PMC8254867; doi:10.1242/dev.199281)
Supplement: Reviewer comments [file develop-148-199281_review_history.pdf]

Laminin alpha 5 regulates mammary gland remodeling through luminal cell differentiation and Wnt4 mediated epithelial crosstalk

Johanna I Englund, Alexandra Ritchie, Leander Blaas, Hanne Cojoc, Nalle Pentinmikko, Julia Döhla, Sharif Iqbal, Manuel Patarroyo and Pekka Katajisto
DOI: 10.1242/dev.199281

Editor: Liz Robertson

Review timeline

Original submission:	2 July 2020
Reject:	2 September 2020
Resubmission:	10 December 2020
Editorial decision:	8 February 2021
First revision received:	19 February 2021
Editorial decision:	22 March 2021
Second revision received:	7 April 2021
Editorial decision:	26 April 2021
Third revision received:	7 May 2021
Accepted:	10 May 2021

Original submission

First decision letter

MS ID#: DEVELOP/2020/194712

MS TITLE: Laminin alpha 5 Regulates Mammary Gland Remodeling Through Luminal Cell Differentiation and Wnt4 Mediated Epithelial Crosstalk

AUTHORS: Johanna Englund, Alexandra Ritchie, Leander Blaas, Hanne Cojoc, Nalle Pentinmikko, Julia Döhla, Sharif Iqbal, Manuel Patarroyo, and Pekka Katajisto

I have now received all the referees' reports on the above manuscript, and have reached a decision. I am sorry to say that the outcome is not a positive one. The referees' comments are appended below, or you can access them online: please go to Development's submission site and click on the 'Manuscripts with Decisions' queue in the Author Area.

As you will see, the referees raise some significant concerns about your paper, and are not strongly in favour of publication. Having looked at the manuscript myself, I agree with their views, and I must therefore, reject your paper.

I do realise this is disappointing news, but Development receives many more papers than we can publish, and we can only accept manuscripts that receive strong support from referees.

I do hope you find the comments of the referees helpful, and that this decision will not dissuade you from considering Development for publication of your future work. Many thanks for sending your manuscript to Development.

Reviewer 1

© 2021. Published by The Company of Biologists under the terms of the Creative Commons Attribution License (https://creativecommons.org/licenses/by/4.0/).

Advance summary and potential significance to field

In this work, Englund et al. studied the role of the alpha-5 laminin chain in mammary gland development. First, using ISH technique the authors analyzed the expression of different laminin chains in mammary epithelium and find that alpha-5 chain is mainly expressed in luminal cells of mammary gland at different developmental stages. Then, they generated a transgenic mouse model, the Krt8creERT2/Lama5f/f mice, in which the alpha-5 laminin chain is conditionally deleted from luminal cells of the mammary epithelium under tamoxifen injection. The mammary phenotype of virgin, pregnant and lactating mutant mice is analyzed. Defects of ductal growth and branching are detected when deletion is induced before puberty. Alveolar formation and lactogenic differentiation appear perturbed during pregnancy and lactation, resulting in a growth defect of the pups fed by mutant females. Finally, data obtained from cell culture analyses provide a potential mechanistic explanation to the observed in vivo phenotype involving signaling downstream Wnt4.

Although the role of laminins on lactogenic differentiation was reported years ago by the pioneer work of Mina Bissell's lab, the reports on the role of laminin chains in mammary gland development in vivo are limited. The present study deserves to be share with the scientific community although a number of issues, mainly concerning further phenotypic analyses of Lama5 mutant mice, must be addressed before publication.

Comments for the author

Main concerns:

The phenotype observed in the mice with alpha-5 laminin chain deletion shows a perturbed organization of the tissue in virgin and pregnant glands. Is this due to polarity defects? Laminins have been reported to be involved in the establishment of cell polarity, for example, during in vivo embryogenesis. Thus, a potential implication of Lama5 in mammary cell polarity deserves to be analyzed.

Krt8creERT2/Lama5f/f animals were analyzed at different developmental stages but the decrease on the expression of Lama5 was not shown in any of them. Lama5 expression should be confirmed at least at the RT-qPCR level on sorted cells.

Specific comments:

Fig.2i, Line 154: the authors mention a loss of general tissue architecture in mutant virgin mice but the figure just shows a couple of structures without lumen and several luminal layers. Are all the ducts perturbed? Is this phenotype found in all mice? If the architecture is really perturbed, this should be better illustrated with further stainings (polarity markers, integrins) and, if possible, quantified.

Fig. 2l, 2m: HR+ population is slightly diminished while Esr1 expression is decreased more than 5-fold. Do the authors have an explanation for this apparent discrepancy? Does PR expression show a similar decrease? This is an important point in view of the results presented later (Fig. 4).

Fig. 3c: The pictures do no illustrate in a reliable way the statements describing the phenotype ("aggregates of luminal cells", "aberrant localization of luminal and basal cells"). Images are very dark, and the stainings hard to see. Please, performed new stainings, or, at least, show pictures of better quality, with magnifications and individual channels for each marker.

Fig. 3e: In contrast to the underdevelopment found at late pregnancy (Fig. 2b), the mutant 2-day-lactating gland appears normally developed. Is this due to an increased proliferation between both time points (P17.5 and PP2) allowing the mutant gland to "catch" the control one? Alveolar morphologically seems also perturbed, with "higher" alveolar cells in mutant. Is this found through the whole gland? Once again I wonder if polarity is affected in this cells.

© 2021. Published by The Company of Biologists under the terms of the Creative Commons Attribution License (https://creativecommons.org/licenses/by/4.0/).

Line 177-178: Quantification of alveolar size should be performed to conclude that “they remain significantly smaller”. What the authors mean with “disorganized” alveoli? The pictures seem to show alveoli without lumen. This can be easily illustrated by a Muc1 staining. In addition, staining with polarity markers (ZO1/Par3) should be performed.

Line 179: the authors claim that mutant glands have fewer lipid droplets in their alveoli (Fig. 3e) but this cannot be appreciated in the picture. A higher magnification should be shown. Staining with a lipid droplet marker (such as adipophilin) would help to better illustrate the difference.

Fig. 4: The expression of Wnt4, previously shown to be induced downstream progesterone signaling, is decreased in mutant luminal cells. Is the expression of PR affected?
In the organoid assays, Rspo1 rescues the branching defects induced by Lama5 deletion in a similar way than Wnt4. Is Rspo1 expression affected in HR- cells in vivo?

Is lactogenic differentiation affected in the organoids model? This could be easily assessed by analyzing casein expression after prolactin treatment of cultured organoids and would provide an explanation to the differentiation phenotype shown in vivo.

Minor points

Line 104: “To quantitatively address expression of Lama1 and Lama5… (Fig. 1c)”.
Only an image for Lama5 ISH is shown in Fig. 1c. For Lama1, only quantification is presented (Fig. 1c).

Fig S1a: The picture showing Lama1 staining do not have at all a duct morphology, it rather seems a vessel. Please, verify or change the picture.

Line 123: “infer that ALSO the luminal epithelial cells may contribute to the laminin pool of the BM”.
I suppose the other cells contributing are the myoepithelial? Please specify.

Fig. 2j: the luminal/basal ration is increased in mutant glands. Is the total amount of epithelial cells perturbed in mutant animals?

Fig. S2e: In the graph showing Sca1 histogram it would be more informative to represent in Y-axis cell % and not cell count.

Reviewer 2

Advance summary and potential significance to field

The manuscript by Englund et al., explores roles for Lama5 in pubertal and gestational mammary gland development. The authors use both in-vivo and in-vitro studies to assess possible roles for laminin a5 in this tissue. They suggest that Lama5 regulates mammary gland remodelling at different developmental stages specifically through regulation of the hormone receptor positive subset of luminal epithelial cells and thus luminal-basal cell cross-talk. The manuscript represents a substantial body of work, however, experimental methods fall short and conclusions are overstated. Development has asked me to assess first whether the advance made in the paper is significant for the field and second whether the data reported in the paper justify the conclusions drawn. These 2 questions are linked and, unfortunately, the answer to both is no.

The authors state in the abstract: “Here we show that Lama5 expression specifically in luminal epithelial cells is necessary for mammary gland growth during puberty, and for alveologenesis and lactation during pregnancy”. Knockdown is poorly described and when it is (organoid model) is low and apparently not cell type specific. Effects are modest in highly variable systems and lactation experiments reveal actually that Lama5 expression is certainly not “necessary”.

The link between wnt-mediated luminal basal cell cross-talk is not adequately demonstrated.

Specific concerns are outlined below.

© 2021. Published by The Company of Biologists under the terms of the Creative Commons Attribution License (https://creativecommons.org/licenses/by/4.0/).

Comments for the author

1. Fig S1a suggests that Lama1 is also expressed in ductal luminal cells.

2. Fig 1C shows strong positivity in the stromal compartment (>> luminal cells, overexposed) but this is not reflected in Fig. 1E (FACS). The authors claim this is "background staining". How has this been determined? What control experiments have been performed with ISH studies to justify this conclusion?

3. Line 116: "However, pan-laminin staining suggested that even the luminally expressed laminin proteins are deposited to the BM surrounding the gland". How does pan-laminin staining support this conclusion?

4. Line 132: "Lama5 deletion in luminal MECs led to…". What was the level of KD in this model? Why was a range (1.5-2mg tamoxifen used)? This is quite a low dose, which is unlikely to cause deletion at both alleles in the majority of cells. Gene deletion must be established at the mRNA or protein level across independent replicates. Related to this, I would suggest editing Fig. 2A to show that control mice received exactly the same injection schedule as Lama5fl/fl;K8CreERT2 mice.

5. Fig 2D. I wonder whether a one-way ANOVA would have been a more suitable test to compare the means of two or more samples, as is the case here. There is a great deal of variability in these data and it is concerning that the majority of control mice have <5 TEBs per gland at 6-weeks of age. In many knockout models elongation may be slightly delayed but later catch up. A longer follow up post-puberty would have been useful. Growth also appears stunted in control mice (2B, 8-wks).

6. Line 136: It may be useful to show these wholemounts. Is ductal end number the most relevant end point to assess when induced after pubertal branching morphogenesis? Would you expect BM turnover in this period of time (tamoxifen to harvest)?

7. Fig. 2I. This may be an artefact of sectioning (i.e. section cutting a curved part of the duct) and it is impossible to tell with this magnified view. This would be visible on standard H&E and could be analyzed blinded by a pathologist.

8. The effects on cell lineage are modest and variable. Several groups have shown an association between Krt8/18 levels and hormone receptor status (e.g., Sleeman et al., 2007, JCB). Koren et al. (2015 Nature) have also shown that the K8-CreERT2 model preferentially labels CD24HiSca1+ luminal cells. It is possible with this model that the hormone sensing luminal subtype may have simply had greater knockdown, rather than Lama5 having a role in luminal cell lineage determination.

9. Fig 3C. I cannot see disorganization of luminal and basal cells. Clear lumen are not always present in fl/fl;Cre+ images, however, this is fairly standard for gestation (prior to secretory activation) and alveoli appear normal at lactation day 2, particularly on the wider field of view in H&E (3E). More importantly, however, pup growth is only very modestly affected. Pups are growing and thriving. Lama5 is clearly not "necessary" as the authors state. The authors should provide additional detail on lactation studies, which are not provided in methods. Litters must be standardized to the same number of pups between control and knockdown groups for ANY real conclusions to be drawn from these data.

10. Line 179. "Importantly, Lama5-deficient glands exhibited fewer lipid droplets than control alveoli (Fig. 3e), suggesting failed functionalization". No quantification has been performed. No lipid stain has been performed.

11. Line 224: "Lama5 deletion resulted in significant reduction in organoid branch formation and elongation (Fig 4c, Fig S3a-b), thereby resembling effects of luminal Lama5 deficiency in-vivo". Was branching assessed in-vivo? How was branching measured in in-vitro model? What are the

© 2021. Published by The Company of Biologists under the terms of the Creative Commons Attribution License (https://creativecommons.org/licenses/by/4.0/).

consequences of switching to a Matrigel model (where basement membrane proteins are provided exogenously)?

12. Fig. S3A suggests very low knockdown (~50%) and yet the authors refer to this as “Lama5 deletion”.

13. Line 194: “Lama5-deficient HR+ luminal MECs are unable to support basal cells” and Line 219 “To directly test whether the altered paracrine Wnt signaling contributes to the defective growth of the mammary gland after luminal Lama5 deletion, we modeled gland growth in vitro”. As per above, the cells are not Lama5 deficient. Did the authors target gene deletion to luminal cells (let alone HR+) or was this performed in mixed MMECs? As per Fig. 1D, ~35% of K14+ basal cells express Lama5.

14. Although not reflected in the graph in S3C, S3E indicates that wnt3a and RSPO1 increase branching and morphology of control cultures as well as Lama5 knockdown. Moreover, K14 staining appears non-uniform in both control and knockdown cultures (e.g., S3E, RSPO1) and may not be the best end-point to assess in these models.

Reviewer 3

Advance summary and potential significance to field

The paper by Eglund et al is a beautiful dissection of Laminin 5 contributions to mammary gland biology in the mouse. The authors describe the localized production of laminin 5 and then proceed to demonstrate the effect of laminin 5 deficiency in several physiological states – pubertal growth, pregnancy and lactation.

Comments for the author

The experiments are compelling. The controls are appropriate and the lay out of the paper is easy to follow and comprehend.

As a reviewer I would have liked to see some data indicating that the results observed were specific to laminin 5 and not other laminins. For example, it would be good for the authors to take ANY of their endpoints and demonstrate that deletion of laminin 1 or 3 or 4 did not demonstrate the same results.

First Submission

Author response to reviewers' comments

We thank all of the reviewers for their constructive comments, and designate our point-to-point responses below with *italics*.

Reviewer 1, Advance Summary and Potential Significance to Field

In this work, Englund et al. studied the role of the alpha-5 laminin chain in mammary gland development. First, using ISH technique the authors analyzed the expression of different laminin chains in mammary epithelium and find that alpha-5 chain is mainly expressed in luminal cells of mammary gland at different developmental stages. Then, they generated a transgenic mouse model, the Krt8creERT2/Lama5f/f mice, in which the alpha-5 laminin chain is conditionally deleted from luminal cells of the mammary epithelium under tamoxifen injection. The mammary phenotype of virgin, pregnant and lactating mutant mice is analyzed.

© 2021. Published by The Company of Biologists under the terms of the Creative Commons Attribution License (https://creativecommons.org/licenses/by/4.0/).

Defects of ductal growth and branching are detected when deletion is induced before puberty. Alveolar formation and lactogenic differentiation appear perturbed during pregnancy and lactation, resulting in a growth defect of the pups fed by mutant females. Finally, data obtained from cell culture analyses provide a potential mechanistic explanation to the observed in vivo phenotype involving signaling downstream Wnt4.

Although the role of laminins on lactogenic differentiation was reported years ago by the pioneer work of Mina Bissell's lab, the reports on the role of laminin chains in mammary gland development in vivo are limited. The present study deserves to be share with the scientific community although a number of issues, mainly concerning further phenotypic analyses of Lama5 mutant mice, must be addressed before publication.

We wish to thank the reviewer for recognizing the value of our work. While the seminal works by Bissell and others (which we highlight in the manuscript) indeed brought in to light the importance of Laminins as a group of molecules, the fact that they have multiple subunits with multiple isoforms each of which has cell type specific expression patterns, warrants the interest to probe which cells actually are responsible for laminin mediated effects on the mammary glands.

Reviewer 1 Comments for the Author: Main concerns:

<u>*Main concerns (Reviewer 1)*</u>:

1) The phenotype observed in the mice with alpha-5 laminin chain deletion shows a perturbed organization of the tissue in virgin and pregnant glands. Is this due to polarity defects? Laminins have been reported to be involved in the establishment of cell polarity, for example, during in vivo embryogenesis. Thus, a potential implication of Lama5 in mammary cell polarity deserves to be analyzed.

*We thank the reviewer for this valuable comment and have looked into the possible effects on Lama5 deletion on cell polarity. We have performed tissue immunostaining and confocal imaging and analyzed pubertal, pregnant and lactating Lama5 deficient and control glands using polarity markers ZO-1 and Mucin. We have added these data into the **Figures 2k, 4d**, and **4g**. While we observe frequent irregularities in the structure of the Lama5 deficient epithelium, we did not observe clear cell polarity defects. In the glands where any luminal surface is visible, both markers localize to the apical surface of single luminal cells albeit at somewhat reduced levels. Moreover, no basal or basolateral localization is seen despite the sometimes very disorganized structure of the Lama5 deficient ducts (**Fig 2k and 4d**). Therefore, we conclude in the revised manuscript that while other laminins have been implicated in mammary cell polarity, Lama5 loss alone does not seem to perturb cell polarity as the primary mechanism underlying observed phenotypes. We hypothesize this is due to the presence of Lama1 and Lama3, which have both been directly implicated in establishment of cell polarity in the mammary gland and both produced by the basal mammary epithelial cells.*

2) Krt8creERT2/Lama5f/f animals were analyzed at different developmental stages but the decrease on the expression of Lama5 was not shown in any of them. Lama5 expression should be confirmed at least at the RT-qPCR level on sorted cells.

*We designed a qPCR strategy to analyze the relative abundance of Lama5 wild-type (WT and conditional allele) and excised mRNA in both pubertal and pregnant glands after tamoxifen treatment (schematically depicted in **Figure S2d**). We use two primer pairs to analyze Lama5 expression, with one binding to exon 67 ("common primers", amplify a product from all alleles) and other primer pair binding to exons 19 and 20/21 ("WT primers", amplify a product only from WT and conditional allele before excision) residing between the LoxP recombination sites and thus binding to the WT or non-floxed allele. Using this strategy, we show in the revised manuscript that after tamoxifen treatment only 20% of the Lama5 mRNA is derived from unexcised conditional allele in the FACS sorted luminal MECs from pubertal*

© 2021. Published by The Company of Biologists under the terms of the Creative Commons Attribution License (https://creativecommons.org/licenses/by/4.0/).

*or pregnant Lama5 fl/fl;K8-Cre animals. These data, indicating approximately 80% recombination efficiency in luminal cells, is shown in the new figure panels **Fig 2a**, **S2d,e** and **S4a** of the revised manuscript.*

1) Fig.2i, Line 154: the authors mention a loss of general tissue architecture in mutant virgin mice but the figure just shows a couple of structures without lumen and several luminal layers. Are all the ducts perturbed? Is this phenotype found in all mice? If the architecture is really perturbed, this should be better illustrated with further stainings (polarity markers, integrins) and, if possible, quantified.

*We thank the referee for this valuable comment. As indicated in our response above to the Main concern relating to polarity, we performed ZO-1 and Mucin stainings, and while these stainings further highlight disorganization in many of the Lama5 deficient ducts, epithelial cell polarity does not appear critically altered. Relating to the comment here about frequency of perturbed glands, we have now performed blinded quantitation of the percentage of ducts with normal (non-filled, round morphology) or disorganized phenotype (partially or complete ductal filling, uncommon or aberrant morphology). As shown in the new **Figure 2i**, majority of ducts in both WT and Lama5 mutant glands are normal. However, the frequency of disorganized ducts is significantly higher in the Lama5 fl/fl;K8-Cre glands. Importantly, the absolute frequency of disorganized ducts in the blind scoring is derived from the whole gland, including the part of the gland that had formed before the Tamoxifen induced Lama5 loss at three weeks of age. We conducted the blinded quantitation for the whole gland in order to avoid any possible bias from selecting regions of the glands.*

2) Fig. 2l, 2m: HR+ population is slightly diminished while Esr1 expression is decreased more than 5-fold. Do the authors have an explanation for this apparent discrepancy?

*This difference is in line with our other data indicating that while HR+ cells are reduced only modestly in frequency, they are also qualitatively altered due to Lama5 loss. In the revised manuscript, we have increased the sample number in the analysis of HR+/HR- cell frequencies and on the mean Sca1 expression of HR+ cells (both showing significant reduction in HR+ cells). Moreover, we now show that in addition to Esr1, also expression of Progesterone receptor (PR) and RANK ligand (RANKL) is reduced in the HR+ cells of Lama deficient glands. These data jointly demonstrating the defective differentiation of the HR+ population are presented in the revised **Figure 3a-f***

3) Does PR expression show a similar decrease? This is an important point in view of the results presented later (Fig. 4).

*As noted above, we have analyzed expression of PR by qPCR from the sorted luminal epithelial cells in pubertal Lama5 deficient and control animals, and also observe a reduction in the PR expression. This data has been added to the new **Figure 3f** and discussed in the text.*

4) Fig. 3c: The pictures do no illustrate in a reliable way the statements describing the phenotype (“aggregates of luminal cells”, “aberrant localization of luminal and basal cells”). Images are very dark, and the stainings hard to see. Please, performed new stainings, or, at least, show pictures of better quality, with magnifications and individual channels for each marker.

*We thank the reviewer for this comment, and agree that some of the original images were dark. We provide new images and higher magnification insets throughout the **new Figure 4**. Together with the new images on polarity markers and markers of gland functionalization, the aberrant organization of the glands is more clearly illustrated.*

5) Fig. 3e: In contrast to the underdevelopment found at late pregnancy (Fig. 2b), the mutant 2-day-lactating gland appears normally developed. Is this due to an increased proliferation between both time points (P17.5 and PP2) allowing the mutant gland to “catch” the control one? Alveolar morphologically seems also perturbed, with “higher” alveolar cells in mutant. Is this found through the whole gland? Once again I wonder if polarity is affected in this cells.

© 2021. Published by The Company of Biologists under the terms of the Creative Commons Attribution License (https://creativecommons.org/licenses/by/4.0/).

*It is possible that the mutant epithelium develops slower to the wild type epithelium during pregnancy but will catch up at lactation. However, in our new quantitation of the alveolar size during pregnancy at dpc 17.5 and at lactation day 2, the alveolar size is similar at dpc 17.5, but it is significantly smaller at lactation day 2 (new **Figure 4e**). This suggests that at least until day 2, the alveolar growth does not catch up. Also, we have performed immunostainings of ZO-1 and Mucin1 of pregnant dpc 17.5 and lactating day 2 glands, and similar to the pubertal glands, observe disorganization of the epithelial architecture. Epithelial cell polarity in these later timepoints is again intact. These new data are in **Figure 4d** and **4g**.*

6) Line 177-178: Quantification of alveolar size should be performed to conclude that “they remain significantly smaller”. What the authors mean with “disorganized” alveoli? The pictures seem to show alveoli without lumen. This can be easily illustrated by a Muc1 staining. In addition, staining with polarity markers (ZO1/Par3) should be performed.

*As stated above, we have performed quantitation of the alveolar size in both dpc 17.5 and lactation day 2 (**Fig 4e**). Also, as suggested and to better illustrate the disorganization of the epithelium, we have performed stainings with ZO-1 and Mucin1 (**Fig 4d** and **4g**). The disorganization we observe in the pregnant and lactating glands is often noted as partially or completely filled ducts with aberrant morphology, as opposed to open and round morphology of the normal ducts. To better articulate these characteristics, we have also added a description in the text lines **176-178** stating:” In the aberrant ducts, K8-positive luminal cells were atypically organized into multiple layers”.*

7) Line 179: the authors claim that mutant glands have fewer lipid droplets in their alveoli (Fig. 3e) but this cannot be appreciated in the picture. A higher magnification should be shown. Staining with a lipid droplet marker (such as adipophilin) would help to better illustrate the difference.

*As suggested, we provide bigger magnifications to accompany the HE stainings in **Figure S4b**. Additionally, we have performed Perilipin immunostaining in the lactating glands to better visualize and quantitate the lipid droplets. The new **Figures 4h,i** demonstrate that the perilipin droplets are significantly smaller in size in the Lama5 lacking glands, but the smaller droplets are super numerous covering larger portion of the total luminal cell area. Jointly these new findings suggest that the lipid droplets do not fuse normally to form milk in the alveoli.*

8) Fig. 4: The expression of Wnt4, previously shown to be induced downstream progesterone signaling, is decreased in mutant luminal cells. Is the expression of PR affected?
In the organoid assays, Rspo1 rescues the branching defects induced by Lama5 deletion in a similar way than Wnt4. Is Rspo1 expression affected in HR- cells in vivo?

*As stated above, we have indeed observed that also PR expression is downregulated in the Lama5 deficient pubertal luminal epithelial cells (**Figure 3f**). Also, we have extensively worked to provide qPCR analysis of Rspo1 expression from the sorted luminal cells. However, Rspo1 appears to be expressed at very low levels rendering analysis difficult, and while we observe a small decrease in the Rspo1 expression in the Lama5 lacking cells, the reduction is not statistically significant (**Figure S5a**). This suggests that HR- epithelial cells are less severely affected by the Lama5 loss than HR+ luminal epithelial cells. Importantly, we provide also new data demonstrating that expression of RANKL - the factor secreted by HR+ cells to promote expression of Rspo1 in the HR- cells - is significantly reduced in vivo (**Fig 3f**).*

9) Is lactogenic differentiation affected in the organoids model? This could be easily assessed by analyzing casein expression after prolactin treatment of cultured organoids and would provide an explanation to the differentiation phenotype shown in vivo.

We thank the reviewer for this insightful suggestion. As suggested, we have set up the organoid model of in vitro lactogenic differentiation following the recently published protocol (Sumbal et al 2020), and assessed the effect of Lama5 deficiency in this process. Lactogenic differentiation was measured by induction of milk protein gene expression, and

© 2021. Published by The Company of Biologists under the terms of the Creative Commons Attribution License (https://creativecommons.org/licenses/by/4.0/).

*occurred robustly in the organoids (**Figure S6a**).*
*Excitingly, loss of Lama5 specifically from the luminal mammary epithelial cells leads to decreased b- casein induction (**Figure 5j**). However, we observed that this phenotype is not rescued by addition of exogenous Wnt4, demonstrating that while Wnt4 can rescue growth phenotype of the Lama5 deficient organoids, it is not sufficient to salvage the effect on functional differentiation. This suggest additional factors, such as those functioning via HR- cells (eg. including RANKL) are ineffective in Lama5 deficient organoids, or that loss of Lama5 affects HR- cells and their capability to functionally differentiate directly. We have also added text to the discussion on **line 366 onwards.***

Minor points (Reviewer 1):

1) Line 104: "To quantitatively address expression of Lama1 and Lama5… (Fig. 1c)".
Only an image for Lama5 ISH is shown in Fig. 1c. For Lama1, only quantification is presented (Fig. 1c).

Image of Lama1 ISH together with Keratin 14 staining has been added into the supplemental data **Figure S1c**.

2) Fig S1a: The picture showing Lama1 staining do not have at all a duct morphology, it rather seems a vessel. Please, verify or change the picture.

We have changed the image in question and replaced it with another one showing Lama1 ISH and presenting more obvious adult duct morphology.

*3) **Line 123: "infer that ALSO the luminal epithelial cells may contribute to the laminin pool of the BM". I suppose the other cells contributing are the myoepithelial? Please specify**.*

We have specified in the text that the other cells implied here to contribute to the BM are indeed the basal epithelial cells. Line 122-126 " Taken together, these results indicate that Laminin isoforms have distinct expression patterns within the mammary gland (summarized in Fig S1g), and infer that also the luminal epithelial cells, in addition to the BM adjacent basal cells, may contribute to the laminin pool of the BM."

4) Fig. 2j: the luminal/basal ration is increased in mutant glands. Is the total amount of epithelial cells perturbed in mutant animals?

*The total amount of epithelial tissue in the Lama5 deficient glands is dramatically diminished (**Figure 2b, e**). In the FACS analysis all epithelium of the gland is collected, digested and processed into single cells before analysis. However, the amounts of stromal cells that are included in the mostly epithelial preparations has marked technical variability, which renders comparison of epithelial/stromal cell frequencies difficult. Nevertheless, we did analyze the frequency of luminal and basal epithelial cells out of the live cells that were negative for hematopoietic linage markers, and observed a decrease in the amounts of epithelial cells in the Lama5 deficient glands (**Figure S3b**). However, reflecting the day-to-day variability in gland preparation, this difference is not statistically significant. Our other analysis (such as the wholemounts Fig 2b) clearly show the reduced size of the whole glands in Lama5 deficient mice, indicating also reduction in epithelial cell quantity.*

5) Fig. S2e: In the graph showing Sca1 histogram it would be more informative to represent in Y-axis cell % and not cell count.

*We have modified the graph so that it now presents the data as unit area (**Figure 3d**). This illustrates the distribution of Sca1 in equally sized populations between genotypes*

© 2021. Published by The Company of Biologists under the terms of the Creative Commons Attribution License (https://creativecommons.org/licenses/by/4.0/).

Reviewer 2, Advance Summary and Potential Significance to Field

The manuscript by Englund et al., explores roles for Lama5 in pubertal and gestational mammary gland development. The authors use both in-vivo and in-vitro studies to assess possible roles for laminin a5 in this tissue. They suggest that Lama5 regulates mammary gland remodelling at different developmental stages specifically through regulation of the hormone receptor positive subset of luminal epithelial cells and thus luminal-basal cell cross-talk. The manuscript represents a substantial body of work, however, experimental methods fall short and conclusions are overstated.
Development has asked me to assess first whether the advance made in the paper is significant for the field and second whether the data reported in the paper justify the conclusions drawn. These 2 questions are linked and, unfortunately, the answer to both is no.

We find the first part of this comment on significance unfounded, but agree that the second part has grounds, and as is evident from our point-to-point responses below, we have now extensively addressed the very constructive comments of the reviewer. Regarding the significance of our work, we have been surprised by the polarized comments on our data's significance to the field. The notion that the role of laminins in the mammary gland is considered to be solved is based on an elegant, but limited set of experiments. These experiments, which we also cite, show that specific laminins (mainly laminin-111 or Lama1) has a great impact.
Other mammary gland researchers have conveyed great excitement towards our surprising findings: 1) that luminal cells produce any laminins in the first place as they are typically illustrated in authoritative reviews not to even have contacts with the BM, 2) a specific laminin in a specific cellular layer will influence the whole gland at least in part by inducing a differentiation defect in one cell type that then affects others - instead of changing direct adhesion for all cells for example. This raises exciting questions regarding various laminin isoforms eliciting different and possibly cell type specific responses, and how laminins could provide almost like a "floorplan" for tissue organization and cell type specific functions. Also of note, Reviewer 1 and 3 commented on the significance of our work very positively.

The authors state in the abstract: "Here we show that Lama5 expression specifically in luminal epithelial cells is necessary for mammary gland growth during puberty, and for alveologenesis and lactation during pregnancy". Knockdown is poorly described and when it is (organoid model) is low and apparently not cell type specific. Effects are modest in highly variable systems and lactation experiments reveal actually that Lama5 expression is certainly not "necessary".

*We thank the reviewer 2 for pointing out these critical shortcomings of our earlier manuscript. In the revised manuscript, we address concerns related to Lama5 targeting by comprehensively describing the allele specific reduction in Lama5 WT mRNA expression in FACS sorted luminal epithelial cells during both puberty and pregnancy. As described above in more detail (response to Reviewer 1 main concern 2), we show in the revised manuscript that after tamoxifen treatment only 20% of the Lama5 mRNA is derived from the unexcised conditional allele in the FACS sorted luminal MECs from pubertal or pregnant Lama5 fl/fl;K8-Cre animals. These data, indicating approximately 80% recombination efficiency in luminal cells, is shown in the new figure panels **Fig 2a**, **S2d,e** and **S4a** of the revised manuscript*

In addition, we fully agree with the referee on the variability of some of our assays on in vivo and primary material. Primarily, we have increased the sample number in many of the experiments of the original manuscript to further boost the confidence on our findings (Figures 2,3,4 and 5). However, we also wish to note that other referees did not raise concerns on variability, and that all our conclusions are based on data that is "statistically significant" in the classical sense. We have chosen to present all of our single data points in accordance with the recent and welcomed trends in driving scientific rigor, openness, and reproducibility. We hope sharing each single datapoint openly has not worked against us in this case.

The link between wnt-mediated luminal basal cell cross-talk is not adequately demonstrated.

The role of Wnt4 in luminal-basal crosstalk has been previously demonstrated in the

© 2021. Published by The Company of Biologists under the terms of the Creative Commons Attribution License (https://creativecommons.org/licenses/by/4.0/).

*literature (Tanos 2012, Brisken 2000, Caj 2014, Joshi 2012). We show that Wnt4 expression is deregulated in Lama5 deficient luminal epithelial cells (**Figure 5a**). Further, we show that the Wnt target gene expression is decreased in basal cells in vivo following luminal specific deletion of Lama5 (**Figure 5b**). In the revised manuscript we show that organoids lacking Lama5 specifically in the luminal cells (derived from Lama5 $^{fl/fl}$; K8-CreERT2 animals after in vivo Tamoxifen treatment) have a defect in the organoid branching – process known to be dependent on luminal-basal cross-talk (Ewald et al., 2008). We also show that this defect is rescued to a level comparable with WT organoids by supplementation of Wnt4. Moreover, we show that organoid branching is dependent on epithelial secretion of Wnt ligands as inhibition of Porcupine by a small molecule inhibitor reduces branching of organoids. These data are shown in the new **Figure 5f,g**, which not only highlight the defective Wnt-mediated cross talk between luminal and basal cells, but also address the previous concern of this reviewer on the phenotypes resulting from targeting in the basal cells. This concern was warranted on the earlier version of the manuscript, where we used lentiviral targeting to delete Lama5 in organoids. The effects we note with the two strategies (luminal specific, and lentiviral deletion) are strikingly similar, further demonstrating that the low level of Lama5 expressed by basal cells is not likely to contribute to the phenotypes we observe.*
*Finally, the new manuscript also shows that besides Wnt4, also expression of the Rank ligand (RANKL) is reduced in HR+ luminal cells after Lama5 deletion (**Figure 3f)**. RANKL promotes expression of another Wnt-signaling factor, Rspo1 in the HR- luminal cells (Joshi et al., 2014) and Rspo1 from luminal cells amplifies Wnt-signaling in the basal cells (Caj et al., 2014). We show that Rspo1 levels are reduced in the luminal cells lacking Lama5 (**Figure S5b)**, but as described in our response to Reviewer 1, Rspo1 detection was technically extremely challenging, and the noted reduction is highly variable and not statistically significant.*

Taken together, our data clearly demonstrate the Wnt-dependent luminal specific defects of Lama5 lacking glands, and suggest that besides reduced Wnt4, changes in the RANKL-Rspo1 axis may further alter the luminal-basal interactions.

Specific concerns are outlined below.

Reviewer 2 Comments for the Author:

1. Fig S1a suggests that Lama1 is also expressed in ductal luminal cells.

*This is correct, in the quantitation of the ISH analysis, 5-10 % of luminal cells express also Lama1, compared to the 80 % of basal cells that express Lama1. As pointed by Reviewer 1, the morphology of the duct in the **Figure S1a** panel was atypical, and therefore, we replaced this image in question with another one showing Lama1 ISH and presenting more typical adult duct morphology. Here the ISH staining more clearly points to Lama1 expression in the basal epithelial cells.*

2. Fig 1C shows strong positivity in the stromal compartment (>> luminal cells, overexposed) but this is not reflected in Fig. 1E (FACS). The authors claim this is “background staining”. How has this been determined? What control experiments have been performed with ISH studies to justify this conclusion?

*To control for the background staining visible in the ISH staining that is performed in conjunction with immunofluorescence staining for cell type markers, we had performed negative control ISH using a probe for a bacterial transcript DapB. This control shows similar non-specific, stromal signal. A representative image of this staining has been added to the **Figure S1b**.*

3. Line 116: “However, pan-laminin staining suggested that even the luminally expressed laminin proteins are deposited to the BM surrounding the gland”. How does pan-laminin staining support this conclusion?

We do not observe the pan-laminin staining outside the ductal BM, hence suggesting that

© 2021. Published by The Company of Biologists under the terms of the Creative Commons Attribution License (https://creativecommons.org/licenses/by/4.0/).

laminins produced by luminal cells are not deposited elsewhere. We have clarified this statement in the sentence on ***line 116*** *"However, the pan-laminin staining that we observed mostly in the periductal region suggested that also the luminally expressed laminin proteins are deposited to the BM surrounding the gland.*

4. Line 132: "Lama5 deletion in luminal MECs led to…". What was the level of KD in this model? Why was a range (1.5-2mg tamoxifen used)? This is quite a low dose, which is unlikely to cause deletion at both alleles in the majority of cells.

*As described in our response to Reviewer 1 main concern 2, the KD level is approximately 80%. We also agree that this is achieved indeed with a relatively low dose of tamoxifen that we had to use due to the small size of some 3-week old mice injected. Majority of the animals received 2 mg of tamoxifen, while some clearly smaller mice received only 1,5 mg. Previous reports (Scheele 2017, Shehata 2014) show that large (>3mg) doses of tamoxifen delay or halt pubertal growth of mammary epithelium and we thus aimed at using a dosage allowing deletion while not hindering analysis of the gland growth. Moreover, we have now performed an additional experiment comparing the epithelial length and TEB amounts in 3 week old WT mice treated with corn oil or 1,5-2mg tamoxifen and show in accordance to previous data that even this low dose tamoxifen treatment leads to decreased epithelial growth and TEB amount during puberty (****Figure S2a****). Therefore, higher doses cannot be used to study Lama5 dependent phenotypes in mammary gland biology. A clarification of this has been added to the materials and methods and to the text* ***line 134*** *onwards.*

Gene deletion must be established at the mRNA or protein level across independent replicates.

We have performed qPCR analysis of the Lama5 expression after tamoxifen treatment during puberty and pregnancy. All experiments have been performed with sorted luminal cells with at least 4 replicates. Data on these experiments has been added to the ***Figures 2a****,* ***S2d-e*** *and* ***S4a****.*

Related to this, I would suggest editing Fig. 2A to show that control mice received exactly the same injection schedule as Lama5fl/fl;K8CreERT2 mice.

The schematic of the ***Figure 2a*** *has been edited to contain Lama5+/+;K8-CreERT2 as well.*

5. Fig 2D. I wonder whether a one-way ANOVA would have been a more suitable test to compare the means of two or more samples, as is the case here.

As suggested, we have tested the data in Fig 2d using Welch's ANOVA (assuming unequal variance).

There is a great deal of variability in these data and it is concerning that the majority of control mice have <5 TEBs per gland at 6-weeks of age. In many knockout models elongation may be slightly delayed but later catch up. A longer follow up post-puberty would have been useful. Growth also appears stunted in control mice (2B, 8-wks).

*This relates to the Reviewer's specific point 4 about the Tamoxifen dose. Tamoxifen reduces the number of TEBs also in control mice. As stated above, we have observed that 1,5-2mg dose of tamoxifen delays the growth of wild type epithelium and decreases TEB numbers (****Figure S2a****), to the level that is comparable to what we seen in the Lama5+/+;Cre control mice (****Figure 2b-d****). In the revised manuscript we do not address the size of the epithelial network in >8 week-old mice, however all data in the Figure 3 is collected from mice at 10 weeks (7 weeks post tamoxifen) suggesting our main phenotype is visible still at this time point.*

6. Line 136: It may be useful to show these wholemounts.

Images of the adult wholemounts have been added to the ***Figure S2g****.*

© 2021. Published by The Company of Biologists under the terms of the Creative Commons Attribution License (https://creativecommons.org/licenses/by/4.0/).

Is ductal end number the most relevant end point to assess when induced after pubertal branching morphogenesis? Would you expect BM turnover in this period of time (tamoxifen to harvest)?

The amount of ductal ends indeed is not necessarily expected to vary greatly at this stage, as the gland does undergo only minor proliferation during the estrous cycle. Also, laminin turnover rate in vivo is currently unknown, and according to previous reports it ranges from 10 hours to few months. Therefore, we do not expect major alterations during the adult homeostatic stage, where minimal growth occurs.

7. Fig. 2I. This may be an artefact of sectioning (i.e. section cutting a curved part of the duct) and it is impossible to tell with this magnified view. This would be visible on standard H&E and could be analyzed blinded by a pathologist.

Similar concern was raised by the Reviewer 1 (specific comment 1), and to address these concerns, we have performed blinded quantitation of the percentage of ducts with normal (non-filled, round morphology) or disorganized phenotype (partially or complete ductal filling, uncommon or aberrant morphology) and have included this quantitation in the ***Figure 2i****. Also, as stated in more detail in response to Reviewer 1, we observe some of the ducts appear normal in morphology also in the Lama5 lacking glands, and we believe this is due to the fact that Lama5 deficiency contributes to the epithelial growth occurring after the tamoxifen treatment, while the ductal tree formed prior to this (up to 3 weeks of age) remains normal and unaffected.*

8. The effects on cell lineage are modest and variable. Several groups have shown an association between Krt8/18 levels and hormone receptor status (e.g., Sleeman et al., 2007, JCB). Koren et al. (2015 Nature) have also shown that the K8-CreERT2 model preferentially labels CD24HiSca1+ luminal cells. It is possible with this model that the hormone sensing luminal subtype may have simply had greater knockdown, rather than Lama5 having a role in luminal cell lineage determination.

We do not argue Lama5 would have a role in lineage determination, but instead we provide data for altered differentiation status in HR+ luminal cells (eg. qPCR in ***Figure 3f****). The change in the relative frequencies of HR+ and HR- luminal cells is indeed modest (Figure 3c). However, the reduction in expression of functional HR+ markers (Esr1, PR) is far greater in luminal cell preps than the reduction in HR+ cell frequency, indicating qualitative changes in the (CD24hi;CD29lo;Sca1hi) HR+ cells.*

9. Fig 3C. I cannot see disorganization of luminal and basal cells. Clear lumen are not always present in fl/fl;Cre+ images, however, this is fairly standard for gestation (prior to secretory activation) and alveoli appear normal at lactation day 2, particularly on the wider field of view in H&E (3E).

*The images such as the Keratin14 staining in Figure 4f clearly show that beyond the filled lumen, Lama5 lacking glands have also atypical localization and organization basal cells. In addition, to better illustrate the altered histology and organization of luminal and basal cells, we have performed ZO-1 and Muc1 immunostainings of the dpc 17.5 and lactating day 2 mammary glands (****Figure 4e and 4g****). These immunostainings further demonstrate the atypical morphology of the ducts observed previously.*

More importantly, however, pup growth is only very modestly affected. Pups are growing and thriving. Lama5 is clearly not “necessary” as the authors state. The authors should provide additional detail on lactation studies, which are not provided in methods. Litters must be standardized to the same number of pups between control and knockdown groups for ANY real conclusions to be drawn from these data.

We agree with the comment that the pup growth is only very modestly affected. However, the litter size of Lama5 fl/fl;Cre mice is significantly smaller (average 3 pups) compared to controls (average 7 pups). Therefore, the ability of the Lama5 deleted mothers to nurse each pup is more dramatically compromised than is evident from the weight gain of pups on

© 2021. Published by The Company of Biologists under the terms of the Creative Commons Attribution License (https://creativecommons.org/licenses/by/4.0/).

*average. The small litter size may reflect Lama5 deficiency in the endometrium that also expresses K8, yet exploring this defect is not in the scope of this study. We have added a remark on the litter size in the text, and have modified the wording in the manuscript and toned down the statement of Lama5 being necessary (text, **line 241**). Also, we have provided information of these studies into the materials and methods sections.*

10. Line 179. “Importantly, Lama5-deficient glands exhibited fewer lipid droplets than control alveoli (Fig. 3e), suggesting failed functionalization”. No quantification has been performed. No lipid stain has been performed.

*We included higher magnification images of the HE stainings showing lipid droplets in lactating glands. In addition, we have performed adipophilin 2 (perilipin) immunostaining in the lactating glands to better illustrate the lipid droplets in the alveoli (**Figure 4h**). Also, we have performed quantification of the size and amount of the lipid droplets in Lama5 deficient and the control glands, showing that in Lama5 deficient glands the droplets are smaller, yet there are more in numbers suggesting either decreased or decelerated fusion of the droplets (as reviewed in Monks et al 2020). This data is added in the **Figure 4h**.*

11. Line 224: “Lama5 deletion resulted in significant reduction in organoid branch formation and elongation (Fig 4c, Fig S3a-b), thereby resembling effects of luminal Lama5 deficiency in-vivo”. Was branching assessed in-vivo? How was branching measured in in-vitro model? What are the consequences of switching to a Matrigel model (where basement membrane proteins are provided exogenously)?

We have used TEB and ductal end number as a marker for branching in vivo. In 3D in vitro model, branching was assessed by number of organoids forming >2 branches. Matrigel does indeed consist of BM proteins, however mainly of collagens and LM-111, and only small amount of Lama5 containing laminins. Therefore, Matrigel mainly provides laminins that are produced by the basal cells, allowing us to probe the function of laminin alpha5 produced by luminal cells. Additionally, it has been demonstrated that cells grown in Matrigel produce their own BM and therefore the role of Lama5 produced by the organoids themselves can be assessed.

12. Fig. S3A suggests very low knockdown (~50%) and yet the authors refer to this as “Lama5 deletion”.

We thanks the Reviewer for this note, as we agree that this is not a complete deletion. We have modified the text, and refer to the cells with Lama5 CRISPR knockdown as “Lama5 edited”.

13. Line 194: “Lama5-deficient HR+ luminal MECs are unable to support basal cells” and Line 219 “To directly test whether the altered paracrine Wnt signaling contributes to the defective growth of the mammary gland after luminal Lama5 deletion, we modeled gland growth in vitro”. As per above, the cells are not Lama5 deficient. Did the authors target gene deletion to luminal cells (let alone HR+) or was this performed in mixed MMECs? As per Fig. 1D, ~35% of K14+ basal cells express Lama5.

*We agree that in these organoid experiments the used mixed population of MECs is not optimal, and does not reflect well the luminal Lama5 deletion. Therefore, we have performed the 3D organoid experiment using cells from Lama5 fl/fl;K8-Cre mice treated with tamoxifen 3 days earlier to delete Lama5 in specifically in luminal epithelial cells, while leaving basal cells wild type (as demonstrated by control experiment on deletion in **Figure S2b**). In this experiment we demonstrate that luminal deletion of Lama5 leads to branch formation deficiency comparable to the CRISPR edited MECs, which is also rescued by addition of exogenous Wnt4. The data on this experiment is added to the **Figure 5f, g and i**.*

14. Although not reflected in the graph in S3C, S3E indicates that wnt3a and RSPO1 increase branching and morphology of control cultures as well as Lama5 knockdown. Moreover, K14 staining appears non-uniform in both control and knockdown cultures (e.g., S3E, RSPO1) and may not be the best end-point to assess in these models.

© 2021. Published by The Company of Biologists under the terms of the Creative Commons Attribution License (https://creativecommons.org/licenses/by/4.0/).

*We agree the number of branches per organoid appears increased also in the wnt3a and RSPO1 treated organoids, however the number of branching organoids does not increase. Also, we have changed the image in **Figure S5f** to better represent Rspo1 treated organoids. We have also added immunostainings of the organoids using E-cadherin (CRISPR organoids, **Figure S5e**) and or E-cadhering together with K8 (endogenous Lama5 fl/fl;K8-Cre and control organoids, **Figure 5i**) to better visualize the organoid morphology.*

Reviewer 3 Advance Summary and Potential Significance to Field

The paper by Eglund et al is a beautiful dissection of Laminin 5 contributions to mammary gland biology in the mouse. The authors describe the localized production of laminin 5 and then proceed to demonstrate the effect of laminin 5 deficiency in several physiological states – pubertal growth, pregnancy and lactation.

Reviewer 3 Comments for the Author:
The experiments are compelling. The controls are appropriate and the lay out of the paper is easy to follow and comprehend.

As a reviewer I would have liked to see some data indicating that the results observed were specific to laminin 5 and not other laminins. For example, it would be good for the authors to take ANY of their endpoints and demonstrate that deletion of laminin 1 or 3 or 4 did not demonstrate the same results.

We thank the Reviewer 3 for the positive comments and for the suggestion to probe the role of other laminins, such as the basal cell expressed Lama3. It would indeed be interesting to address to what extent possible phenotypes are dependent on the specific function of laminin isoforms, and to what extend characterized by the cell type expressing them. However, we feel this is outside the scope of our current study where we purposefully aimed at providing a thorough analysis of the isoform with the most strikingly luminal expression.

Resubmission

First decision letter

MS ID#: DEVELOP/2020/199281

MS TITLE: Laminin alpha 5 Regulates Mammary Gland Remodeling Through Luminal Cell Differentiation and Wnt4 Mediated Epithelial Crosstalk

AUTHORS: Johanna I Englund, Alexandra Ritchie, Leander Blaas, Hanne Cojoc, Nalle Pentinmikko, Julia Dohla, Sharif Iqbal, Manuel Patarroyo, and Pekka Katajisto

I have now received all the referees reports on the above manuscript, and have reached a decision. The referees' comments are appended below, or you can access them online: please go to BenchPress and click on the 'Manuscripts with Decisions' queue in the Author Area.

The overall evaluation is positive and we would like to publish a revised manuscript in Development, provided that the referees' comments can be satisfactorily addressed. Please attend to all of the reviewers' comments in your revised manuscript and detail them in your point-by-point response. If you do not agree with any of their criticisms or suggestions explain clearly why this is so.

We are aware that you may currently be unable to access the lab to undertake experimental revisions. If it would be helpful, we encourage you to contact us to discuss your revision in greater

© 2021. Published by The Company of Biologists under the terms of the Creative Commons Attribution License (https://creativecommons.org/licenses/by/4.0/).

detail. Please send us a point-by-point response indicating where you are able to address concerns raised (either experimentally or by changes to the text) and where you will not be able to do so within the normal timeframe of a revision. We will then provide further guidance. Please also note that we are happy to extend revision timeframes as necessary.

Reviewer 1

Advance summary and potential significance to field

The manuscript by England et al. investigates the role of laminin alpha-5 in mammary gland development. After analysing the expression of different laminin alpha chains by in situ hybridization, the authors focus on Lama5, mostly (but not exclusively) expressed by luminal cells. The main body of the paper describes the mammary phenotype of the Krt8cre-ert2/Lama5flox/flox mice, in which the alpha-5 laminin chain is conditionally deleted from luminal cells of the mammary epithelium after tamoxifen injection. Ductal development is perturbed when deletion is induced in pre-pubertal females while alveologenesis and lactogenic differentiation appear impaired during pregnancy and lactation. Organoid culture analyses provide a potential mechanistic explanation to this mammary phenotype implicating paracrine luminal-basal interactions involving Wnt4 signalling.

Numerous works have reported the involvement of integrin receptors and their downstream signalling effectors in postnatal mammary gland development and differentiation. However, little is know about the in vivo role of laminins, expected to be major in vivo ligands of integrins in the mammary epithelium, given their abundance in the mammary basement membrane. The present study reveals interesting data concerning this item, although some important questions should be addressed prior to publication.

Comments for the author

Specific concerns
It has been widely reported that several integrin receptors - integrins containing the b1-chain (DOI: 10.1038/sj.emboj.7600674; doi: 10.1083/jcb.200503144 and others) and, more specifically, laminin-binding integrins (doi: 10.1016/j.ydbio.2019.10.001; doi: 10.7554/eLife.06104) - are necessary for correct lactogenesis. The work of Mina Bissell's and Charles Streuli's groups using culture models showed that integrin-mediated adhesion to laminin co-operates with prolactin to potentiate the activation of STAT5, a key transcription factor orchestrating lactogenic differentiation. Is the expression of integrin receptors to LM521-LM511 affected in mutant (Lama5 deficient) mice? A hypothetical role of Itgb4 (that, combined with Itga6 forms a6b4 dimer, a component of hemidesmosmes) is discussed. However, other dimers with high affinity for laminin are present in the mammary epithelium. Furthermore, as cited by the authors, a previous study reports that Itgb4 expression is higher in HR+ than HR- cells (at the transcriptional level). What about basal cells? They are known to express much higher levels of laminin-binding integrins than the luminal populations. More importantly, the authors neglect the fact that basal cells also express Lama5 (Fig. 1d-e). These items deserve to be, at least, commented.

The analysis of the mammary phenotype in mutant virgin mice comprises some stainings of polarity markers (Zo-1, MUC1, Fig. 2k). The authors mention that localization of these markers was "largely unaffected" and conclude that Lama5 is necessary for "proper duct architecture independent of cellular polarity effects". I do not agree with these interpretations and conclusions. First, Zo-1 expression is clearly diminished in mutants (this is already sign of defects) making very difficult to conclude about its localization. For MUC1, the mutant picture shows a lumen clearly lacking MUC1 and some "inner cells" (not in contact with lumen), displaying MUC1. These expressions and localizations are aberrant, and, in contrast to the claimed by the authors, indicate polarity defects in the mutant epithelium.

Fig. S3b (this Fig. is not referenced in the text, please correct) shows no difference in the epithelial content of control and mutant mammary glands. This is surprising, given the big difference shown in the whole-mount staining (Fig 2b). Have the authors an explanation for this inconsistency? I noticed that Fig. S3b display results for 5 controls and 8 mutant mice, suggesting

© 2021. Published by The Company of Biologists under the terms of the Creative Commons Attribution License (https://creativecommons.org/licenses/by/4.0/).

that these are not paired results. To avoid variability between different sorting experiments, this kind of comparison should be done with paired data obtained with littermate mice.

l. 215: “alveolar development was dramatically reduced in Lama5 deficient glands as evidenced by fewer alveoli compared to control glands (Fig4a-b)”. I guess the alveolar density in mutant mice is variable in different areas of the gland, since the pictures of the stainings (Fig. 4c-d) show an alveolar number similar to the found in control animals. A quantification of alveolar density is required.

l. 220: “the apico-basal polarity of Lama5-deficient glands appeared unaffected also at this stage of pregnancy”. Again, I do not agree with this interpretation. E-cadherin is lacking (or presents a weak staining) in some areas of mutant epithelium, while MUC1, that in control appears as a thin apical line, in some luminal mutant cells presents cytoplasmic localization (Fig 4d). In the same line, in 2-day-lactating glands, E-cadherin localizes baso-laterally in control, while basal localization is missing in mutant epithelium (Fig 4g). These results probably reflect a delay in alveolar maturation, but at least deserve to be interpreted and discussed in a proper way.

l.265: “Rspo1 showed only a trend of modest reduction” (Fig S5a). This is surprising, since Rspo1 is induced by RANKL that, in Fig. 3f is practically absent from mutant luminal cells. Since Rspo1 transcript levels have been analysed in total luminal population, the small reduction detected may reflect the fact that the mutants have increased HR- population (the luminal population expressing Rspo1), attenuating the differences. Ideally, Rspo1 expression should be assessed in sorted HR- cells. Alternatively, a bigger number of luminal cell samples can help to reach significance (for basal gene expression, 7-10 samples have been analysed in Fig. 5b, why not here?). The lack of significant decrease of Rspo1 expression is also unexpected in view of the results displayed in Fig. 5e, where Rspo1 addition contributes to the rescue of branching defects in mutant organoids.

At least, the result presented in Fig. S5a deserves some explanation/comment.

l. 315 : « Wnt4 further increased the Csn2 expression in controls ». This is unexpected for me, has the influence of Wnt4 in lactogenesis been previously reported? In vivo, Wnt4 induces active branching morphogenesis of the gland that allows growth and subsequent alveologenesis. One can hypothesize that, in this way, Wnat4 may favour Csn2 expression. However, in the organoids model, as shown in Fig. 5c, Wnt4 treatment does not affect branching in the control situation. Have the authors an explanation for this result?

l. 369 « Wnt4 was unable to rescue lactogenic differentiation in organoids and the authors point to lack of other essential factors ». RANKL seems also important for alveologenesis, and Fig. 3f shows that its expression is almost abolished in virgin glands. Is its expression affected also during pregnancy? Could RANKL rescue the lactogenic differentiation in the organoids model?

Minor comments
References to Figures in the text must be checked and corrected, I detected many mistakes:

l. 106: The reference to Fig. S1b is not correct, it should be Fig. S1c.

l. 155: The reference to Fig. S2f is not correct, it should be Fig. S2g-h.

l. 164: Fig. S2g is referenced, but it does not correspond with the results presented in this Fig.

l. 166: Fig. S2e is referenced, but it does not correspond with the results presented in this Fig.

l. 292: The reference to Fig. 5g-h is not correct, it should be Fig. 5f-g.

l. 301: The reference to Fig. 5i is not correct, it should be Fig. 5h.

The Fig. S6b is not referenced in the text. Which is the difference with Fig. 5j ?

© 2021. Published by The Company of Biologists under the terms of the Creative Commons Attribution License (https://creativecommons.org/licenses/by/4.0/).

Fig. S1d: the scheme presenting the FACS strategy is misleading: in the last two windows, one could think that is the stroma population (and not the luminal population) that is taken for the Sca1/CD49b display.

Fig. S1g: the scheme should reflect the fact that, although at lower levels than luminal, basal cells also express Lama5. Please, correct.

l. 301: "upon Wnt4 treatment": in Fig S5g is not Wnt4, but Wnt3a that was used to treat the organoids (at least, is what is written in the Figure). Please, correct.

Legend to Fig. S5a: Only Rspo1 expression in luminal cells is shown (and not in basal and luminal as written). Please, correct.

l. 362 : "we conclude that the altered luminal-basal interactions, which are mediated by Wnt signaling explain the defective growth and remodeling of Lama5-deficient glands". This sentence should be tune down since other factors might be involved in vivo and the presented data do not show that defective Wnt signalling is at the origin of all the observed phenotypes.

<u>Reviewer 2</u>

Advance summary and potential significance to field
N/A

Comments for the author

Substantial new data have been provided, which help to support some of the original conclusions. Two major issues remain.

1. The authors are unable to make any claims about a lactation phenotype in their mouse model, having not standardized for litter size and having huge variability in their cohorts (avg 7 for control vs 3 for fl/fl/cre+ mice). With three (and presumably sometimes less!) pups per litter for fl/fl;cre+ mothers, it is highly likely that many of the glands analyzed in this study were not actively suckled.

The authors attempt to justify this flaw in experimental design by stating: "pups gained weight significantly slower, in spite of their smaller litter size".

Competition for milk in mice (10 mammary glands) is not likely to be a major contributing factor in this case, and rather glands are likely to be under-utilized and poorly developed and likely to be undergoing precocious involution.

I would strongly suggest either 1) removing the in vivo lactation data completely and limiting conclusions to those supported by gestation and organoid data or 2) repeating all lactation endpoints on mice with standardized litters.

2. Validation of KO. Two reviewers asked for demonstration of knockdown in this model. I am confused why the authors didn't use the same qPCR strategy utilized in Fig 1e (or is this what we see in the middle two n.s. bars in S2e??). The assay used in 1a or 1c with similar quantification would also be acceptable.

Other comments:
New Fig 1c is problematic. There is a great deal of background staining that is not present in the negative control. How confident are the authors in their ability to manually count positive and negative luminal and basal cells from these studies?

The authors should include P values in all legends.

Why has a paired t-test been used in 3e? Why has a one-tailed student's t-test been used in 5j?

Please consult with statistician here and throughout.

© 2021. Published by The Company of Biologists under the terms of the Creative Commons Attribution License (https://creativecommons.org/licenses/by/4.0/).

Related to Fig 4, can the authors include data for number of viable embryos in each group at 17.5dpc if these data were collected during the harvests?

4a flfl/cre+ gland is overexposed. Please provide n=3 examples as an additional supp figure (ideally) or in peer review file to help convey potential variability in these data. Alternatively, quantify histology in b.

Page 9 line 217: “Moreover, Lama5-deficient alveoli often failed to form round structures, and were instead devoid of distinct lumen and exhibited aberrant organization of both luminal and basal cells (Fig. 4c).” This statement is at odds with the 17.5 dpc data in e, which shows no significant difference in alveolar diameter.

Page 12, line 316. “However, although Wnt4 further increased the Csn2 expression in controls, and it rescued the branching defect of Lama5-deficient organoids as before, it did not rescue the defective lactogenic induction in Lama5-deficient organoids.”
This is not supported by statistical analysis in 5j. If multiple comparisons are being made consider using a different test.

Fig 5j appears to have a very large error bar for +/+;Cre+ wnt4 LM condition. Have some datapoints dropped off the graph with the split axes? Please clarify and alter the display.

Reviewer 3

Advance summary and potential significance to field

In this manuscript, Englund et al. describe a role for laminin alpha 5 (Lama5) in mammary gland remodeling. Specifically, they show that loss of Lama5, normally expressed by luminal epithelial cells, results in a moderate reduction in the number of HR+ cells – and in a more substantial defect Wnt4-mediated crosstalk with basal cells.

Laminins are known to be important components of the basement membrane, which offers structural support for epithelial cells. Integrity of the basement membrane is critical for tissue development and becomes disrupted when tumor cells invade the surrounding stroma. It is thus as critical for developmental processes as the cellular parts of the tissue.

Multiple Laminins are expressed in the mammary gland. The study begins with an interesting observation, namely that Lama5 is almost exclusively expressed by luminal (and especially HR+) cells. This is counter intuitive, because typically basal cells are depicted as contacting the basement membrane. Indeed, the authors confirm that the luminal cells contact the basement membrane – something that has been observed before and by others (including myself) but as far as I know has never been stated so explicitly.

Deletion of Lama5 in the pubertal mammary gland reduced outgrowth and branching morphogenesis and normal tissue architecture was lost, with atypical organization into additional layers. Moreover, HR+ cells are specifically reduced in number in the Lama5 deficient gland. Deletion of Lama5 in the adult gland seemingly inhibits or delays alveologenesis and milk formation.

Comments for the author

I think this is a beautiful and carefully executed study, whose sole caveat is that it remains unclear how the loss of Lama5 results in the gene expression differences observed. Specifically a moderate loss of HR+ cells, but a far more dramatic loss in HR+ cell specific gene expression occurs upon the loss of Lama5: A ~10% reduction in HR+ cells results in ~50% reduction in PR levels and almost completely abolishes Rankl expression. I would not know how to answer this question either, and I think the study is sufficiently strong as it is.

© 2021. Published by The Company of Biologists under the terms of the Creative Commons Attribution License (https://creativecommons.org/licenses/by/4.0/).

Figures and figure legends are clear and well annotated. Error bars etc. are well described and individual data points are shown.

Remaining Comments:
1. The authors suspect disruption of cell polarity in Lama5 null glands (page 8), but state that they find no differences in MUC1 and ZO1 expression. This is a bit unclear, since the overall levels are reported to be reduced (line 184/185). For ZO1 this is hard to ascertain from the image provided, so perhaps this can still be textually clarified.

2. Line 266/Figure 5B: what evidence do the authors have for the fact that Ctnnb1 is a Wnt-responsive gene? Typically, the activity of Ctnnb1 is thought to be controlled at the protein stability, not the RNA expression level. A reference for Ctnnb1 and Sox9 would be great if it can be added. Ideally the authors would have looked at the expression of a common Wnt target gene (e.g. Axin2).

3. In figure 5D-G the rescuing effects of Wnt4 are relatively subtle and it is not clear whether Wnt4 has a clear effect by itself and/or an additional effect on Rspo1. In 5H-I the effect is much more clear, although it remains unstated what the real effect is: Lama5 deletion seems to prevent Fgf2 mediated branching morphogenesis. How this is rescued by Wnt4 remains unclear.

4. In lines 303/305 the authors refers to the fact that Wnt signaling promotes basal cell function, but in fact the Wnt4 treated Lama5 deficient organoids in 5I are actually 'naked' (i.e. don't have basal cells at the branching tips).

Second revision

Author response to reviewers' comments

We thank all of the reviewers for their constructive comments. We are currently restricted in our experimental work by the local regulations relating to the ongoing pandemic, but have also managed to generate new data addressing reviewer comments with existing samples. Please find our point-to-point responses to reviewer comments below (*in italics*).

Reviewer 1 Advance Summary and Potential Significance to Field
The manuscript by England et al. investigates the role of laminin alpha-5 in mammary gland development. After analysing the expression of different laminin alpha chains by in situ hybridization, the authors focus on Lama5, mostly (but not exclusively) expressed by luminal cells. The main body of the paper describes the mammary phenotype of the Krt8cre-ert2/Lama5flox/flox mice, in which the alpha-5 laminin chain is conditionally deleted from luminal cells of the mammary epithelium after tamoxifen injection. Ductal development is perturbed when deletion is induced in pre-pubertal females while alveologenesis and lactogenic differentiation appear impaired during pregnancy and lactation. Organoid culture analyses provide a potential mechanistic explanation to this mammary phenotype implicating paracrine luminal-basal interactions involving Wnt4 signalling. Numerous works have reported the involvement of integrin receptors and their downstream signalling effectors in postnatal mammary gland development and differentiation. However, little is know about the in vivo role of laminins, expected to be major in vivo ligands of integrins in the mammary epithelium, given their abundance in the mammary basement membrane. The present study reveals interesting data concerning this item, although some important questions should be addressed prior to publication.

> *We thank the reviewer for recognizing the value of our work. While the previous studies, including those by Bissell and others report the role of laminin binding receptors in the mammary gland, our work highlights the importance of Laminins as a diverse group of molecules with cell type specific expression patterns and effects in mammary gland.*

© 2021. Published by The Company of Biologists under the terms of the Creative Commons Attribution License (https://creativecommons.org/licenses/by/4.0/).

Reviewer 1 Comments for the Author:

Specific concerns It has been widely reported that several integrin receptors - integrins containing the b1- chain (DOI: 10.1038/sj.emboj.7600674; doi: 10.1083/jcb.200503144 and others) and, more specifically, laminin-binding integrins (doi: 10.1016/j.ydbio.2019.10.001; doi: 10.7554/eLife.06104) - are necessary for correct lactogenesis. The work of Mina Bissell's and Charles Streuli's groups using culture models showed that integrin-mediated adhesion to laminin co-operates with prolactin to potentiate the activation of STAT5, a key transcription factor orchestrating lactogenic differentiation. Is the expression of integrin receptors to LM521-LM511 affected in mutant (Lama5 deficient) mice? A hypothetical role of Itgb4 (that, combined with Itga6 forms a6b4 dimer, a component of hemidesmosmes) is discussed. However, other dimers with high affinity for laminin are present in the mammary epithelium. Furthermore, as cited by the authors, a previous study reports that Itgb4 expression is higher in HR+ than HR- cells (at the transcriptional level).

> *We thank the reviewer for this insightful comment. Integrins are indeed shown to be necessary for mammary gland morphogenesis and lactogenic differentiation, especially integrin b1. However, in our analyses we did not observe changes in the b1-integrin expression (measured in FACS using CD29 antibody eg in **Fig 3a**) in Lama5 deleted luminal or basal mammary epithelial cells compared to cells from control animals. This can reflect the dependency on other adhesion molecules for BM adhesion, but also redundancy between the integrin receptors. We have currently another project probing the distinct functions of specific laminin-integrin pairings (particularly in the basal mammary cells), and feel that including this complicated aspect is beyond the scope of our current manuscript, and would distract from the main message.*

What about basal cells? They are known to express much higher levels of laminin-binding integrins than the luminal populations. More importantly, the authors neglect the fact that basal cells also express Lama5 (Fig. 1d-e). These items deserve to be, at least, commented.

> *Additionally, as pointed out by the reviewer, approx. 35 % of basal cells do produce Lama5, yet only half of the amount of what luminal cells do. In addition, they also produce other laminins including Lama1 and Lama3. Therefore, it is conceivable that the most abundant laminins in the in vivo environment after luminal cell specific Lama5 deletion are the basally produced laminins. Additionally, we have shown in our preprint (https://www.biorxiv.org/content/10.1101/2019.12.27.889451v1) containing earlier version of the present manuscript, that whereas adhesion of mammary epithelial cells to LM521 promotes luminal gene expression, adhesion to LM111 promotes basal gene expression. Moreover, adhesion to LM111 also supports other basal progenitor traits. As these findings suggest that intrinsically basal cells should thrive in the LM521/511 low surroundings resulting from luminal Lama5 deletion, but our findings in this current manuscript demonstrate that they do not, we focus on the discovered deficiency in paracrine signaling between the luminal and basal epithelial cells.*

The analysis of the mammary phenotype in mutant virgin mice comprises some stainings of polarity markers (Zo-1, MUC1, Fig. 2k). The authors mention that localization of these markers was "largely unaffected" and conclude that Lama5 is necessary for "proper duct architecture independent of cellular polarity effects". I do not agree with these interpretations and conclusions. First, Zo-1 expression is clearly diminished in mutants (this is already sign of defects) making very difficult to conclude about its localization. For MUC1, the mutant picture shows a lumen clearly lacking MUC1 and some "inner cells" (not in contact with lumen), displaying MUC1. These expressions and localizations are aberrant, and, in contrast to the claimed by the authors, indicate polarity defects in the mutant epithelium.

> *We thank the reviewer for this important comment. In our imaging data, as stated in the text, we observed frequent unorganization and decrease in the staining intensity of ZO-1 and MUC1 polarity markers. However, ZO-1 and MUC-1 were mostly localized apically as in controls. Proper localization of polarity markers is one of the key elements of polarity, which we here paid attention to, and therefore concluded that loss of Lama5 does not lead to complete loss of polarity. However, we agree to the reviewers' notion that inner luminal cells have aberrant localization of MUC1 indeed suggesting polarity defect, especially in the*

© 2021. Published by The Company of Biologists under the terms of the Creative Commons Attribution License (https://creativecommons.org/licenses/by/4.0/).

pubertal mutant epithelium. Therefore, we decided to modify our conclusions and the text accordingly:

*From **line 184** onwards: "However, Lama5$^{fl/fl}$:K8-CreERT2 ducts showed great variability in intensity and localization of the polarity markers, suggesting that loss of Lama5 from the luminal epithelium affects luminal cell polarity but the polarity is not completely lost. Interestingly, both ZO-1 and MUC1 also exhibited a dramatic reduction in general staining intensity in the Lama5-deficient ducts. MUC1 is a differentiation marker of luminal mammary epithelial cells (Shehata et al., 2012), raising the possibility of defective luminal differentiation in Lama5 deleted glands. In conclusion, Lama5 in the luminal epithelial cells is necessary for normal pubertal mammary gland growth and properly polarized duct architecture."*

Fig. S3b (this Fig. is not referenced in the text, please correct) shows no difference in the epithelial content of control and mutant mammary glands. This is surprising, given the big difference shown in the whole-mount staining (Fig 2b). Have the authors an explanation for this inconsistency? I noticed that Fig. S3b display results for 5 controls and 8 mutant mice, suggesting that these are not paired results. To avoid variability between different sorting experiments, this kind of comparison should be done with paired data obtained with littermate mice.

The Fig 3b, which is now referenced in the text, shows the proportions of epithelial cells out of hematopoietic lineage negative cells instead of total cell numbers. We indeed observe a great difference in the epithelial content of the Lama5 deficient and control animals, however, in the MEC isolation protocol used throughout the paper, we purposefully enrich for epithelial cells. This is done by manually cutting the glands and collagenase digestion to separate the epithelium from the fat pad, followed by several centrifugation steps to clear the remaining blood cells, adipocytes and other stromal cells. This process concentrates the epithelial fragments, which will then be trypsinized and prepaired for Facs analysis. Therefore, the analysis shows proportions of collected epithelial and stromal cells. Indeed, this ratio does not change significantly between the genotypes, which strengthens the conclusions we make on changed ratios between various epithelial cell types.
*Clarification has been added to the **S3b figure and legend**: "Graph showing the percentage of all epithelial cells in lineage negative (Lin-) live cell population.."*

l. 215: "alveolar development was dramatically reduced in Lama5 deficient glands as evidenced by fewer alveoli compared to control glands (Fig4a-b)". I guess the alveolar density in mutant mice is variable in different areas of the gland, since the pictures of the stainings (Fig. 4c-d) show an alveolar number similar to the found in control animals. A quantification of alveolar density is required.

*We thank the reviewer for this comment. We have now quantitated the density of the alveoli in dpc 17.5 mice, and indeed observed a difference in the density of alveoli between Lama5 lacking and control epithelium. New data has been added to the **Fig S4b**, and in the text Line 217: "Alveolar development was dramatically reduced in Lama5 deficient glands as evidenced by fewer and less densely packed alveoli compared to control glands (Fig 4a-b, FigS4b)"*

l. 220: "the apico-basal polarity of Lama5-deficient glands appeared unaffected also at this stage of pregnancy". Again, I do not agree with this interpretation. E-cadherin is lacking (or presents a weak staining) in some areas of mutant epithelium, while MUC1, that in control appears as a thin apical line, in some luminal mutant cells presents cytoplasmic localization (Fig 4d). In the same line, in 2-day-lactating glands, E-cadherin localizes baso-laterally in control, while basal localization is missing in mutant epithelium (Fig 4g). These results probably reflect a delay in alveolar maturation, but at least deserve to be interpreted and discussed in a proper way.

As discussed above in point 2 related to the polarity defect during puberty, we agree there is diminished staining in both E-cadherin and MUC-1. However, we again do not observe marked mislocalization of the used polarity markers and therefore, we interpret also here that the

© 2021. Published by The Company of Biologists under the terms of the Creative Commons Attribution License (https://creativecommons.org/licenses/by/4.0/).

polarity is affected but not completely lost both in pregnant and lactating Lama5 lacking mice. To reflect this conclusion and the reviewer's comments we have added a sentence in the text stating: ***Line 223****: "However, the apicobasal polarity of Lama5-deficient glands appeared only modestly affected at this stage of pregnancy (Fig 4d), yet intriguingly, also here MUC1 staining intensity was reduced."*

Furthermore, to reflect this view of apico-basal polarity being more intact in the pregnant and lactating glands, we also modified the discussion accordingly: ***Line 395****: "We noted that apico-basal polarity is affected also in the Lama5-deficient luminal epithelial cells, suggesting it may contribute to the surplus of luminal cells during puberty. Cell polarity was better conserved in the Lama5-deficient glands during pregnancy and lactation, suggesting the most aberrant glands may not be able to contribute to the pregnancy induced gland remodeling. However, addressing the exact order of events downstream of Lama5 deletion and loss of tissue architecture during puberty requires further experiments."*

l.265: "Rspo1 showed only a trend of modest reduction" (Fig S5a). This is surprising, since Rspo1 is induced by RANKL that, in Fig. 3f is practically absent from mutant luminal cells. Since Rspo1 transcript levels have been analysed in total luminal population, the small reduction detected may reflect the fact that the mutants have increased HR- population (the luminal population expressing Rspo1), attenuating the differences. Ideally, Rspo1 expression should be assessed in sorted HR- cells. Alternatively, a bigger number of luminal cell samples can help to reach significance (for basal gene expression, 7-10 samples have been analysed in Fig. 5b, why not here?). The lack of significant decrease of Rspo1 expression is also unexpected in view of the results displayed in Fig. 5e, where Rspo1 addition contributes to the rescue of branching defects in mutant organoids. At least, the result presented in Fig. S5a deserves some explanation/comment.

We agree with this comment that the modest increase in Rspo1 is quite surprising. We believe this may in part reflect technical challenges in detecting Rspo1 in qPCR, which also underlies the variability in the sample number. The original qPCR was performed with a larger number of samples (6 wt vs 7 fl), but we excluded cases with alarmingly low Ct values from the presented analysis. We fully agree with the reviewer that the analysis would be ideally performed from HR- cells, but as yield of HR- epithelial cells will restrict the material dramatically, and we particularly would reduce the number of analysed cells from the Lama5 fl/fl;K8-Cre mice, we feel the limited material would introduce further risk of bias when analysing such a lowly expressed gene.

l. 315 : Wnt4 further increased the Csn2 expression in controls. This is unexpected for me, has the influence of Wnt4 in lactogenesis been previously reported? In vivo, Wnt4 induces active branching morphogenesis of the gland that allows growth and subsequent alveologenesis. One can hypothesize that, in this way, Wnat4 may favour Csn2 expression. However, in the organoids model, as shown in Fig. 5c, Wnt4 treatment does not affect branching in the control situation. Have the authors an explanation for this result?

We agree with this comment that the increase in Csn2 expression by Wnt4 is unexpected. We hypothesize indeed that the increase occurs due to increase in branching morphogenesis. However, for the obvious lack of branching in Lama5 deficient organoids, and as branch sizes can also vary, we considered quantitating the frequency of branching/non-brancing organoids to present a more relevant metric instead of quantitating the number of individual branches per organoid. Importantly, the lactogenic differentiation assay has two phases: first organoids are induced to branch, and in the second phase milk production is induced by lactogenic hormones (Sumbal et al 2020). Hence, As Wnt4 alone increases the number and size of branches within the organoids during the first phase, it is likely to increase also Csn2 expression as a secondary effect to branching.

l. 369 « Wnt4 was unable to rescue lactogenic differentiation in organoids and the authors point to lack of other essential factors ». RANKL seems also important for alveologenesis, and Fig. 3f shows that its expression is almost abolished in virgin glands. Is its expression affected also during pregnancy? Could RANKL rescue the lactogenic differentiation in the organoids model?

© 2021. Published by The Company of Biologists under the terms of the Creative Commons Attribution License (https://creativecommons.org/licenses/by/4.0/).

This is a very interesting and relevant question. Infact, we have long planned to perform this exact experiment to test whether RANKL could rescue lactogenic differentiation in the organoids. Unfortunately, we were (and still are, after waiting for over 12 months now) unable to obtain any recombinant Wnt4 from the commercial providers. After consultation with the company, we have learned about manufacturing difficulties and the product is actually now discontinued from several providers.

Minor comments

References to Figures in the text must be checked and corrected, I detected many mistakes:
l. 106: The reference to Fig. S1b is not correct, it should be Fig. S1c.

l. 155: The reference to Fig. S2f is not correct, it should be Fig. S2g-h.

l. 164: Fig. S2g is referenced, but it does not correspond with the results presented in this Fig.
l. 166: Fig. S2e is referenced, but it does not correspond with the results presented in this Fig.

l. 292: The reference to Fig. 5g-h is not correct, it should be Fig. 5f-g.

l. 301: The reference to Fig. 5i is not correct, it should be Fig. 5h.

The Fig. S6b is not referenced in the text. Which is the difference with Fig. 5j ?

We thank the reviewer for pointing out these obvious mistakes. They have all been corrected, and additionally, we have changed the Figure S2f panel to Fig S2h so that the panels will be presented in correct sequence as indicated in the text.

Fig. S1d: the scheme presenting the FACS strategy is misleading: in the last two windows, one could think that is the stroma population (and not the luminal population) that is taken for the Sca1/CD49b display

Fig S1d schematic has been changed to better illustrate that luminal population is shown in Sca1/CD49b plot.

Fig. S1g: the scheme should reflect the fact that, although at lower levels than luminal, basal cells also express Lama5. Please, correct.

Fig S1g schematic has been changed to better indicate also basal cells express Lama5.

l. 301: "upon Wnt4 treatment": in Fig S5g is not Wnt4, but Wnt3a that was used to treat the organoids (at least, is what is written in the Figure). Please, correct.

The reference has been corrected.

Legend to Fig. S5a: Only Rspo1 expression in luminal cells is shown (and not in basal and luminal as written). Please, correct.

This has been corrected in the figure legend.

l. 362 : "we conclude that the altered luminal-basal interactions, which are mediated by Wnt signaling explain the defective growth and remodeling of Lama5-deficient glands". This sentence should be tune down since other factors might be involved in vivo and the presented data do not show that defective Wnt signalling is at the origin of all the observed phenotypes.

We have modified the following sentence as follows: "Thus, we conclude that the altered luminal-basal interactions, which are mediated by Wnt signaling can in part explain the defective growth and remodeling of Lama5-deficient glands".

© 2021. Published by The Company of Biologists under the terms of the Creative Commons Attribution License (https://creativecommons.org/licenses/by/4.0/).

Reviewer 2 Advance Summary and Potential Significance to Field

Reviewer 2 Comments for the Author:

Substantial new data have been provided, which help to support some of the original conclusions. Two major issues remain. 1. The authors are unable to make any claims about a lactation phenotype in their mouse model, having not standardized for litter size and having huge variability in their cohorts (avg 7 for control vs 3 for fl/fl/cre+ mice). With three (and presumably sometimes less!) pups per litter for fl/fl;cre+ mothers, it is highly likely that many of the glands analyzed in this study were not actively suckled. The authors attempt to justify this flaw in experimental design by stating: "pups gained weight significantly slower, in spite of their smaller litter size". Competition for milk in mice (10 mammary glands) is not likely to be a major contributing factor in this case, and rather glands are likely to be under- utilized and poorly

developed and likely to be undergoing precocious involution. I would strongly suggest either 1) removing the in vivo lactation data completely and limiting conclusions to those supported by gestation and organoid data or 2) repeating all lactation endpoints on mice with standardized litters.

*We agree that the experimental design in our lactation experiment has not been ideal. However, in all the cases we analyzed #4 glands both in Lama5 deleted and control glands, and did observe in the PP2 timepoint morphology typical for the lactating gland in all of the cases. Consequently, we consider that these experiments, together with our data from the pregnant glands, support our conclusions on defective differentiation of mammary epithelium. However, as mentioned above, we acknowledge the shortcomings in our in vivo weight gain data/experiment, and as suggested by the referee, have now removed the data on the pup weight gain (**Fig 2l**).*

3) Validation of KO. Two reviewers asked for demonstration of knockdown in this model. I am confused why the authors didn't use the same qPCR strategy utilized in Fig 1e (or is this what we see in the middle two n.s. bars in S2e??). The assay used in 1a or 1c with similar quantification would also be acceptable.

*This is correct, the assay used in Fig 1e is the same as used in Fig S2e (common primers). As the used conditional Lama5 allele produces a truncated mRNA product after Cre mediated excision (Nguyen et al 2005), we designed a qPCR strategy to analyze the relative abundance of Lama5 wild-type and excised mRNA (using wild type and common primers; schematically depicted in **Figure S2d**). We show in the figure S2e that the levels of the "Common" transcript are not changed, while the level of the "WT" transcript is reduced and this reduction is quantified.*

Other comments: New Fig 1c is problematic. There is a great deal of background staining that is not present in the negative control. How confident are the authors in their ability to manually count positive and negative luminal and basal cells from these studies?

*We assume the reviewer means **Fig S1c** here as the Fig 1c does not contain a comparison to negative control. In any case, we agree this assay has considerable amount of background staining, but the background staining is easily discerned as it is mainly located outside the epithelium (also in Fig s1b) and does not exhibit the hallmark punctate pattern of the real signal with RNAscope technology. Therefore, we are not exactly sure what the reviewer means here, but we utilized widely used K8 and K14 antibodies to label the luminal and basal epithelial cells. In summary, we are quite confident in our analyses picking up the differences within the specific epithelial cell population.*

The authors should include P values in all legends.

These have been added

© 2021. Published by The Company of Biologists under the terms of the Creative Commons Attribution License (https://creativecommons.org/licenses/by/4.0/).

Why has a paired t-test been used in 3e?

We assume the reviewer means Fig 3d here, where mean intensity of Sca1 is measured. Here we have used paired t-test (and pairs indicated by lines in the figure), as each dot pair in the figure represents data from an individual experiment containing 3-4 mice. The experiment are performed on separate days, and as antibody labelings will vary between days due to myriad technical reasons, we have paired the analysis between control and Lama5 deficient cells for each day.

Why has a one-tailed student's t-test been used in 5j? Please consult with statistician here and throughout.

*One-tailed t-test was used here to test the possibility on Lama5 loss leading to **reduction** in Csn2 expression, which we hypothesized based on our previous results. We have consulted a statistician in regard of all of the statistics we have performed.*

Related to Fig 4, can the authors include data for number of viable embryos in each group at 17.5dpc if these data were collected during the harvests?

We do not presently have the data.

4a flfl/cre+ gland is overexposed. Please provide n=3 examples as an additional supp figure (ideally) or in peer review file to help convey potential variability in these data. Alternatively, quantify histology in b.

Due to the translucency of the samples the wholemount samples are difficult to image with equal lighting. However, we have performed new quantitation of the alveolar density in Fig 4b as suggested by Reviewer
*1. The data is found in **Fig S4b**.*

Page 9 line 217: "Moreover, Lama5-deficient alveoli often failed to form round structures, and were instead devoid of distinct lumen and exhibited aberrant organization of both luminal and basal cells (Fig. 4c)." This statement is at odds with the 17.5 dpc data in e, which shows no significant difference in alveolar diameter.

We find this statement not to be at odds with the data, as this might simply reflect the increase in luminal cells also seen in pubertal mice, and suggesting that surplus cells are filling the lumen as seen in the micrographs.

Page 12, line 316. "However, although Wnt4 further increased the Csn2 expression in controls, and it rescued the branching defect of Lama5-deficient organoids as before, it did not rescue the defective lactogenic induction in Lama5-deficient organoids." This is not supported by statistical analysis in 5j. If multiple comparisons are being made consider using a different test.

We have added statistical testing for the comparison of Untreated +/+;Cre vs Wnt4 treated +/+;Cre, which shows significant difference between these groups. Related to the point raised by the reviewer earlier, also statistical test with repeated measures anova shows significant difference between the groups.

Fig 5j appears to have a very large error bar for +/+;Cre+ wnt4 LM condition. Have some datapoints dropped off the graph with the split axes? Please clarify and alter the display.

The large error bar in the graph is due to the large variation in the Csn2 induction (Fig S6b shows the raw fold change values individually plotted for each experiment) in the Wnt4 treated +/+;Cre samples. We decided to split the axis indeed for better representation of the variation, yet it inflates the error bar at the lower end of the axis. All the data points are visible in the graph.

© 2021. Published by The Company of Biologists under the terms of the Creative Commons Attribution License (https://creativecommons.org/licenses/by/4.0/).

Reviewer 3 Advance Summary and Potential Significance to Field

In this manuscript, Englund et al. describe a role for laminin alpha 5 (Lama5) in mammary gland remodeling. Specifically, they show that loss of Lama5, normally expressed by luminal epithelial cells, results in a moderate reduction in the number of HR+ cells – and in a more substantial defect Wnt4-mediated crosstalk with basal cells. Laminins are known to be important components of the basement membrane, which offers structural support for epithelial cells. Integrity of the basement membrane is critical for tissue development and becomes disrupted when tumor cells invade the surrounding stroma. It is thus as critical for developmental processes as the cellular parts of the tissue. Multiple Laminins are expressed in the mammary gland. The study begins with an interesting observation, namely that Lama5 is almost exclusively expressed by luminal (and especially HR+) cells. This is counter intuitive, because typically basal cells are depicted as contacting the basement membrane. Indeed, the authors confirm that the luminal cells contact the basement membrane – something that has been observed before and by others (including myself) but as far as I know has never been stated so explicitly. Deletion of Lama5 in the pubertal mammary gland reduced outgrowth and branching morphogenesis and normal tissue architecture was lost, with atypical organization into additional layers. Moreover, HR+ cells are specifically reduced in number in the Lama5 deficient gland. Deletion of Lama5 in the adult gland seemingly inhibits or delays alveologenesis and milk formation.

Reviewer 3 Comments for the Author:

I think this is a beautiful and carefully executed study, whose sole caveat is that it remains unclear how the loss of Lama5 results in the gene expression differences observed. Specifically, a moderate loss of HR+ cells, but a far more dramatic loss in HR+ cell specific gene expression occurs upon the loss of Lama5: A ~10% reduction in HR+ cells results in ~50% reduction in PR levels and almost completely abolishes Rankl expression. I would not know how to answer this question either, and I think the study is sufficiently strong as it is. Figures and figure legends are clear and well annotated. Error bars etc. are well described and individual data points are shown.

We thank the reviewer for these positive comments and valuing our work. We find it indeed a very relevant and interesting question that how does loss of Lama5 lead to alterations in the HR+ gene expression. However, we feel also this is not easily addressed and it warrants further studies on the subject.

Remaining Comments: 1. The authors suspect disruption of cell polarity in Lama5 null glands (page 8), but state that they find no differences in MUC1 and ZO1 expression. This is a bit unclear, since the overall levels are reported to be reduced (line 184/185). For ZO1 this is hard to ascertain from the image provided, so perhaps this can still be textually clarified.

*We thank the reviewer for the comment. A similar notion was also made by reviewer #1 and as described in our response above, we observed frequent unorganization and decrease in the staining of ZO-1 and MUC1 polarity markers. However, both markers were mostly localized in a correct manner, which is one of the key elements of polarity that we here paid attention to and therefore, we concluded that loss of Lama5 does not lead to complete loss of polarity. However, we agree to the notion that diminished levels of the polarity markers themselves are suggestive of a polarity defect, and thus, we have altered the main text accordingly: From **line 184** onwards: "However, Lama5$^{fl/fl}$:K8-CreERT2 ducts showed great variability in intensity and localization of the polarity markers, suggesting that loss of Lama5 from the luminal epithelium affects luminal cell polarity but the polarity is not completely lost. Interestingly, both ZO-1 and MUC1 also exhibited a dramatic reduction in general staining intensity in the Lama5-deficient ducts. MUC1 is a differentiation marker of luminal mammary epithelial cells (Shehata et al., 2012), raising the possibility of defective luminal differentiation in Lama5 deleted glands. In conclusion, Lama5 in the luminal epithelial cells is necessary for normal pubertal mammary gland growth and properly polarized duct architecture."*

*Also, prompted by the reviewers, we made a notion that Lama5 loss effect on cell polarity appears more pronounced in the pubertal epithelium compared to the pregnant and lactating epithelium. Hence, we also modified the discussion accordingly: **Line 395**: "We noted that apico-basal polarity is affected also in the Lama5-deficient luminal epithelial cells,*

© 2021. Published by The Company of Biologists under the terms of the Creative Commons Attribution License (https://creativecommons.org/licenses/by/4.0/).

suggesting it may contribute to the surplus of luminal cells during puberty. Cell polarity was better conserved in the Lama5-deficient glands during pregnancy and lactation, suggesting the most aberrant glands may not be able to contribute to the pregnancy induced gland remodeling. However, addressing the exact order of events downstream of Lama5 deletion and loss of tissue architecture during puberty requires further experiments."

2. Line 266/Figure 5B: what evidence do the authors have for the fact that Ctnnb1 is a Wnt-responsive gene? Typically, the activity of Ctnnb1 is thought to be controlled at the protein stability, not the RNA expression level. A reference for Ctnnb1 and Sox9 would be great if it can be added. Ideally the authors would have looked at the expression of a common Wnt target gene (e.g. Axin2).

We originally performed qPCR on the well-known Wnt target axin2 as well. However, it was expressed at a surprisingly low levels in the mammary gland (our laboratory has extensive experience on Wnt-signaling in the intestine), and could not be reliably detected in our assays. Therefore, we used Ctnnb1 and Sox9 (in addition to Lgr5), which have been shown to be Wnt target genes (Guo et al., 2012, van Schie and van Amerongen, 2020, Plaks et al., 2013).

3. In figure 5D-G the rescuing effects of Wnt4 are relatively subtle and it is not clear whether Wnt4 has a clear effect by itself and/or an additional effect on Rspo1. In 5H-I the effect is much more clear, although it remains unstated what the real effect is: Lama5 deletion seems to prevent Fgf2 mediated branching morphogenesis. How this is rescued by Wnt4 remains unclear.

We fully concur with this comment. Although we observe increase in the branching morphogenesis with Wnt4 +/- Rspo1 in Lama5 deficient organoids, it is presently unclear what are the exact mechanisms downstream of Wnt signaling leading to proper branching morphogenesis. We hope to address this interesting question with future work, however, we feel it is outside the scope of the present work.

4. In lines 303/305 the authors refers to the fact that Wnt signaling promotes basal cell function, but in fact the Wnt4 treated Lama5 deficient organoids in 5I are actually 'naked' (i.e. don't have basal cells at the branching tips).

We have made the same observation and agree with the Reviewer's comment, but have been hesitant to make any claims on the matter due to variability of the phenotype. The difference might also reflect the difference in the targeting strategy (CRISPR vs K8-Cre), where either luminal only or luminal and basal cells are both Lama5 deficient. In summary, branching organoids in general are quite variable, and with changing targeting strategies we feel making claims based on the phenotype would be premature.

Second decision letter

MS ID#: DEVELOP/2020/199281

MS TITLE: Laminin alpha 5 Regulates Mammary Gland Remodeling Through Luminal Cell Differentiation and Wnt4 Mediated Epithelial Crosstalk

AUTHORS: Johanna I Englund, Alexandra Ritchie, Leander Blaas, Hanne Cojoc, Nalle Pentinmikko, Julia Dohla, Sharif Iqbal, Manuel Patarroyo, and Pekka Katajisto

I have now received all the referees' reports on the above manuscript, and have reached a decision. The referees' comments are appended below, or you can access them online: please go to BenchPress and click on the 'Manuscripts with Decisions' queue in the Author Area.

© 2021. Published by The Company of Biologists under the terms of the Creative Commons Attribution License (https://creativecommons.org/licenses/by/4.0/).

As you will see, the referees express interest in your work, but have some significant criticisms and recommend a further substantial revision of your manuscript before we can consider publication. In particular the lactation experiments seem problematic and overall the data are highly variable, maybe as a consequence of the efficiency of the in vivo tamoxifen based excision. The reviewers also have the same concern about the overall novelty of the manuscript, suggesting the study is somewhat incremental. If you are able to revise the manuscript along the lines suggested, which may involve further experiments and more extensive statistical analysis, I will be happy receive a revised version of the manuscript. Your revised paper will be re-reviewed by one or more of the original referees, and acceptance of your manuscript will depend on your addressing satisfactorily the reviewers' major concerns. Please also note that Development will normally permit only one round of major revision.

We are aware that you may be experiencing disruption to the normal running of your lab that make experimental revisions challenging. If it would be helpful, we encourage you to contact us to discuss your revision in greater detail. Please send us a point-by-point response indicating where you are able to address concerns raised (either experimentally or by changes to the text) and where you will not be able to do so within the normal timeframe of a revision. We will then provide further guidance. Please also note that we are happy to extend revision timeframes as necessary.

Please attend to all of the reviewers' comments and ensure that you clearly highlight all changes made in the revised manuscript. Please avoid using 'Tracked changes' in Word files as these are lost in PDF conversion. I should be grateful if you would also provide a point-by-point response detailing how you have dealt with the points raised by the reviewers in the 'Response to Reviewers' box. If you do not agree with any of their criticisms or suggestions please explain clearly why this is so.

Reviewer 1

Advance summary and potential significance to field

The manuscript by England et al. investigates the role of laminin alpha-5 in mammary gland development. After analysing the expression of different laminin alpha chains by in situ hybridization, the authors focus on Lama5, mostly (but not exclusively) expressed by luminal cells. The main body of the paper describes the mammary phenotype of the Krt8cre-ert2/Lama5flox/flox mice, in which the alpha-5 laminin chain is conditionally deleted from luminal cells of the mammary epithelium after tamoxifen injection. Ductal development is perturbed when deletion is induced in pre-pubertal females while alveologenesis and lactogenic differentiation appear impaired during pregnancy and lactation. Organoid culture analyses provide a potential mechanistic explanation to this mammary phenotype implicating paracrine luminal-basal interactions involving Wnt4 signalling.

Numerous works have reported the involvement of integrin receptors and their downstream signalling effectors in postnatal mammary gland development and differentiation. However, little is know about the in vivo role of laminins, expected to be major in vivo ligands of integrins in the mammary epithelium, given their abundance in the mammary basement membrane. The present study reveals interesting data concerning this item.

Comments for the author

In the revised manuscript, Englund and colleagues have addressed my main criticisms to the work. Although no new experiments have been presented, quantification and re-interpretation of some of the existing data improve the quality and understanding of the study. Most of the points I raised have been answered in the rebuttal letter and some are now discussed in the manuscript. Concerning my first point, the potential involvement of the main laminin receptors, the integrins, I understand that the authors want to analyse their specific role in a separated new project. However, I still think that integrin's role in lactogenic differentiation should be further discussed, and the references to published old and recent studies that I mentioned in my first review, cited.

Reviewer 2

© 2021. Published by The Company of Biologists under the terms of the Creative Commons Attribution License (https://creativecommons.org/licenses/by/4.0/).

Advance summary and potential significance to field
N/A

Comments for the author

As mentioned in my previous review the lactation experiments are not appropriately conducted to draw any conclusions about lactation (subheading currently reads: "laminin a5 is necessary for alveologenesis and lactation"). Experiments have not been conducted in a manner that permits these conclusions.
In response to my query about analysis of knockdown using this rather unconventional method, the authors state that the conditional Lama5 allele produces a truncated mRNA product after cre mediated excision, citing Nguyen et al. The paper that they cite does not mention stable truncated mRNA. This paper appears to quantify KD at the protein level. Relative mRNA levels have been determined using the ddCt method. So the authors have normalized to housekeeping gene and then analyzed Cre+ with respect to control (as is standard). The third normalizaition to the common primer in unconventional. Fig S2E shows that for the WT primer there is no significant difference between control and cre+ mice. Theoretically, there should be no significant difference between the common primer in control and cre+ mice (which there isn't), but yet there is a difference when they ratio the fold change.
I still have concerns with statistics and statistical transparency. As previously mentioned when making multiple comparisons an ANOVA is generally preferable to multiple t-tests. The new Fig S4B shows 12 data points, however the related figure discusses use of 3-4 animals. What is considered an independent replicate here for statistical analysis is not stated in the figure legend.
Other,
Per Reviewer #1 comments, if conclusions are now being drawn about intensity of MUC1, ZO1 etc, the laser power, gain, b&c adjustment etc must be constant and this needs to be stated.

Reviewer 3

Advance summary and potential significance to field

I did not have too many comments on the previous version of the manuscript, so I will not bring up any additional points here.

The authors and I agree that the Wnt4 mechanism remains incompletely understood and I also don't see how this would be easily solved.

Comments for the author

One point remains, and I may want to stick to my guns here:
My previous comment 2.
Line 266/Figure 5B: what evidence do the authors have for the fact that Ctnnb1 is a Wnt-responsive gene? Typically, the activity of Ctnnb1 is thought to be controlled at the protein stability, not the RNA expression level. A reference for Ctnnb1 and Sox9 would be great if it can be added. Ideally the authors would have looked at the expression of a common Wnt target gene (e.g. Axin2).
The authors' reply:
We originally performed qPCR on the well-known Wnt target axin2 as well. However, it was expressed at a surprisingly low levels in the mammary gland (our laboratory has extensive experience on Wnt-signaling in the intestine), and could not be reliably detected in our assays. Therefore, we used Ctnnb1 and Sox9 (in addition to Lgr5), which have been shown to be Wnt target genes (Guo et al., 2012, van Schie and van Amerongen, 2020, Plaks et al., 2013).
My reply to this:
The authors are correct that Axin2 expression levels in the mammary gland are quite low, but one would expect them to increase upon WNT/CTNNB1 activation, although it is possible that they remain below the detection limit of the qRT-PCR the authors performed.
I do not agree, however, that the papers cited by the authors show that Ctnnb1 and Sox9 are WNT/CTNNB1 target genes. If the authors do find this information in the two papers and/or the one

© 2021. Published by The Company of Biologists under the terms of the Creative Commons Attribution License (https://creativecommons.org/licenses/by/4.0/).

review cited, I kindly ask that they point me to the precise point where this information is provided in these references (particulary for the fact that Ctnnb1 is a WNT target gene), because in that case I will stand corrected (and I may have to reconsider or correct some of my own work).
This may come across as nitpicking and/or a semantic discussion, but I find it very important that the literature record is properly cited and reflected before new dogmas are created that will only confuse the field (there is enough that is still unknown about the role of WNT signalling in the mammary gland and this includes the target gene repertoire).

Third revision

Author response to reviewers' comments

We thank all of the reviewers for their continued input and constructive comments. Our point-to-point responses are below.

Reviewer 1 Advance Summary and Potential Significance to Field

The manuscript by England et al. investigates the role of laminin alpha-5 in mammary gland development. After analysing the expression of different laminin alpha chains by in situ hybridization, the authors focus on Lama5, mostly (but not exclusively) expressed by luminal cells. The main body of the paper describes the mammary phenotype of the Krt8cre-ert2/Lama5flox/flox mice, in which the alpha-5 laminin chain is conditionally deleted from luminal cells of the mammary epithelium after tamoxifen injection. Ductal development is perturbed when deletion is induced in pre-pubertal females while alveologenesis and lactogenic differentiation appear impaired during pregnancy and lactation. Organoid culture analyses provide a potential mechanistic explanation to this mammary phenotype implicating paracrine luminal-basal interactions involving Wnt4 signalling.

Numerous works have reported the involvement of integrin receptors and their downstream signalling effectors in postnatal mammary gland development and differentiation. However, little is know about the in vivo role of laminins, expected to be major in vivo ligands of integrins in the mammary epithelium, given their abundance in the mammary basement membrane. The present study reveals interesting data concerning this item.

Reviewer 1 Comments for the Author:
In the revised manuscript, Englund and colleagues have addressed my main criticisms to the work. Although no new experiments have been presented, quantification and re-interpretation of some of the existing data improve the quality and understanding of the study. Most of the points I raised have been answered in the rebuttal letter and some are now discussed in the manuscript. Concerning my first point, the potential involvement of the main laminin receptors, the integrins, I understand that the authors want to analyse their specific role in a separated new project. However, I still think that integrin's role in lactogenic differentiation should be further discussed, and the references to published old and recent studies that I mentioned in my first review, cited.

We again thank the reviewer for these insightful comments and recognizing the value of our work.

Concerning the point about the role of integrins in lactogenic differentiation, we agree that they play an important role in both mammary development and functional differentiation. Therefore, we have added the following paragraph into the discussion (Line 340):
"Our results are in concert with previous studies showing that downregulation of laminin-binding adhesion receptors, including b1- and b4-integrins, results in inhibition of mammary epithelial growth and alveologenesis (Li 2005, Naylor 2005, Walker 2020, Di-Cicco 2015). Together these data underline the importance of laminin and BM microenvironment in regulation of mammary epithelial growth and differentiation."
We believe this addition covers the points made by Reviewer 1, without clashing with the point by Reviewer 2 on lactation. We will not refer to lactation (only to alveologenesis and for

© 2021. Published by The Company of Biologists under the terms of the Creative Commons Attribution License (https://creativecommons.org/licenses/by/4.0/).

example production of milk proteins).
Reviewer 2 Advance Summary and Potential Significance to Field
-
Reviewer 2 Comments for the Author:
As mentioned in my previous review the lactation experiments are not appropriately conducted to draw any conclusions about lactation (subheading currently reads: "laminin a5 is necessary for alveologenesis and lactation"). Experiments have not been conducted in a manner that permits these conclusions.

We agree with the reviewer (as stated in our previous rebuttal letter), that the experimental design in our experiments where weight gain and lactation has not been ideal. Therefore, we already removed the weight gain data. However, in all the cases where we study the glands during lactation, we analyzed only the same "gland number 4" mammary gland both in Lama5 deleted and control animals, and did observe in the PP2 timepoint morphology typical for the lactating gland in all of the cases. Therefore, we feel that data referring to the morphology, cellular polarity and organization, p-STAT5 status, lipid droplet size and quantity, and milk protein production, are valid for this time point during early days of nursing.

We have in any case toned down our conclusions on the lactation experiments, in addition to what was done earlier, and will alter our wording to fully remove the word lactation from the manuscript where we refer to our own work (including subheading and the abstract).

The above-suggested edits fully cover what we understood was the point of the reviewer. However, it is somewhat unclear to us what reviewer precisely means by "lactation experiments". As mentioned above, the manuscript of course contains multiple types of data related to the functionalization of the gland (Figure 4E-K) that are downstream relevant for lactation – even though we will not make that point in the revised version. In case the reviewer actually refers to these data, we hope a specific suggestion relating to these data would be made.

In response to my query about analysis of knockdown using this rather unconventional method, the authors state that the conditional Lama5 allele produces a truncated mRNA product after cre mediated excision, citing Nguyen et al. The paper that they cite does not mention stable truncated mRNA. This paper appears to quantify KD at the protein level. Relative mRNA levels have been determined using the ddCt method. So the authors have normalized to housekeeping gene and then analyzed Cre+ with respect to control (as is standard). The third normalizaition to the common primer in unconventional. Fig S2E shows that for the WT primer there is no significant difference between control and cre+ mice. Theoretically, there should be no significant difference between the common primer in control and cre+ mice (which there isn't), but yet there is a difference when they ratio the fold change.

We thank the reviewer for spotting this obvious mistake in the Figure S2E, which made the result confusing. The labelling on Fig. S2E should state WT/common primer like as in Fig 2A (not common/WT as it was on the S2E). The mistake will be corrected into the Fig S2E.

As noted by the reviewer, the PCR probes detecting both full length Lama5 mRNA and the truncated mRNA formed post excision, named 'common' show no difference in their expression levels (ddCt, normalized to Gapdh and relative to +/+, Fig. S2E leftmost comparison). However, the probes detecting specifically mRNA at the excised part of the gene, named 'WT', show lowered expression post excision (ddCt normalized to Gapdh and relative to +/+, Fig. S2E center comparison).

Also, we have termed the mRNA found in the Ngyuen et al 2005 paper mistakenly as "truncated mRNA". It is true that the paper does not describe a truncated mRNA, but rather states that there is an out-of-frame RNA produced after the Cre-mediated excision has occurred. However, the paper does not show the presence or absence of the mRNA, and hence this notion is only anecdotal. We on the other hand data show that mRNA covering exon 67 is indeed produced in the cells after Cre-mediated excision.

We decided to use the described qPCR method since other methods were not feasible. We have tried several Lama5 antibodies for mouse tissues, and could not detect it reliably either in

© 2021. Published by The Company of Biologists under the terms of the Creative Commons Attribution License (https://creativecommons.org/licenses/by/4.0/).

paraffin embedded or frozen sections. Also, using RNA in situ hybridization and quantitation similar to Figure 1C is not an option due to the presence of the Lama5 mRNA in both +/+ and fl/fl animals (the Lama5 RNA in situ reagents we use consists a pool of probes, and can therefore also detect the floxed mRNA), resulting in staining also in the fl/fl;Cre animals.

I still have concerns with statistics and statistical transparency. As previously mentioned when making multiple comparisons an ANOVA is generally preferable to multiple t-tests. The new Fig S4B shows 12 data points, however the related figure discusses use of 3-4 animals. What is considered an independent replicate here for statistical analysis is not stated in the figure legend.

We apologize for the unclear explanation in the figure legend related to FigS4B. In the Fig S4B we have two groups (+/+ 6 vs fl/fl 4 individual animals), and multiple images per each mice used in the analysis are plotted as dots in the graph, to openly show the variability within the samples. The statistical testing has here been done using the t-test, which is standard when comparing two groups instead of ANOVA.

To address the reviewer point, a more clear explanation of this and the statistical testing has been added to the figure legend S4B:
"(B) Quantitation of percentage of ductal area in mammary glands of dpc 17.5 pregnant mice. Graph shows percentage of ductal density from 6 +/+;Cre and 5 fl/fl;Cre individual animals with each dot representing one image analysed. Two-tailed student's t-test was used to compare the groups as whole. Right panel show representative original HE image and the corresponding binary image used for quantitation of ductal density (white areas)."

Regarding the overall statistics transparency, as we have stated in our earlier rebuttal letter, all the used statistical tests and number of animals/replicates are listed, and moreover we plot all individual values of each experiment in the graphs throughout the manuscript – going beyond the requirements of Development on data transparency. This should also allow the reader to better evaluate our data, also regardless the chosen statistical testing.

Other,
Per Reviewer #1 comments, if conclusions are now being drawn about intensity of MUC1, ZO1 etc, the laser power, gain, b&c adjustment etc must be constant and this needs to be stated.

We have added the following statement into the materials and methods section, Line 543: "Same settings for laser power and detector gain were maintained within one experiment. "

Reviewer 3 Advance Summary and Potential Significance to Field

I did not have too many comments on the previous version of the manuscript, so I will not bring up any additional points here.

The authors and I agree that the Wnt4 mechanism remains incompletely understood and I also don't see how this would be easily solved.

Reviewer 3 Comments for the Author:
One point remains, and I may want to stick to my guns here:

My previous comment 2.
Line 266/Figure 5B: what evidence do the authors have for the fact that Ctnnb1 is a Wnt- responsive gene? Typically, the activity of Ctnnb1 is thought to be controlled at the protein stability, not the RNA expression level. A reference for Ctnnb1 and Sox9 would be great if it can be added. Ideally the authors would have looked at the expression of a common Wnt target gene (e.g. Axin2).

The authors' reply:
We originally performed qPCR on the well-known Wnt target axin2 as well. However,

© 2021. Published by The Company of Biologists under the terms of the Creative Commons Attribution License (https://creativecommons.org/licenses/by/4.0/).

it was expressed at a surprisingly low levels in the mammary gland (our laboratory has extensive experience on Wnt-signaling in the intestine), and could not be reliably detected in our assays. Therefore, we used Ctnnb1 and Sox9 (in addition to Lgr5), which have been shown to be Wnt target genes (Guo et al., 2012, van Schie and van Amerongen, 2020, Plaks et al., 2013).

My reply to this:
The authors are correct that Axin2 expression levels in the mammary gland are quite low, but one would expect them to increase upon WNT/CTNNB1 activation, although it is possible that they remain below the detection limit of the qRT-PCR the authors performed.

I do not agree, however, that the papers cited by the authors show that Ctnnb1 and Sox9 are WNT/CTNNB1 target genes. If the authors do find this information in the two papers and/or the one review cited, I kindly ask that they point me to the precise point where this information is provided in these references (particulary for the fact that Ctnnb1 is a WNT target gene), because in that case I will stand corrected (and I may have to reconsider or correct some of my own work).

This may come across as nitpicking and/or a semantic discussion, but I find it very important that the literature record is properly cited and reflected before new dogmas are created that will only confuse the field (there is enough that is still unknown about the role of WNT signalling in the mammary gland and this includes the target gene repertoire).

We agree with the reviewer that the current knowledge about Wnt target genes in the mammary gland is limited. As stated previously, Axin2 was indeed below the limit of detection in some of the FACS sorted samples (with cell numbers <5000), and we could therefore not make conclusions on its expression.
Therefore we used the Wnt target gene database curated by Roel Nusse laboratory (https://web.stanford.edu/group/nusselab/cgi-bin/wnt/target_genes) to aid in finding targets.

However, we acknowledge our mistake and apologize not citing the correct work regarding the other targets. The articles mentioned speculated on the Sox9 and CTTNB1 being Wnt target genes, but did not conclusively provide evidence. However, both genes have been shown to be regulated by Wnt in other tissues including the intestine (SOX9: Blache et al 2004, CTTNB1: Bikkavilli & Malbon, 2010). Specifically, the latter work shows that CTTNB1 can be regulated by Wnt, yet CTTNB1 levels are not regulated conventionally via transcriptional regulation, but through a mechanisms involving mRNA stability. Therefore, we have corrected the citations to include these papers, and re-phrased the text in line 266:
"Moreover, expression of Wnt-responsive genes Sox9 and Lgr5 (Blache et al 2004, Shuijers & Clevers 2012), regulated directly via transcriptional control or Ctnnb1 regulated via mRNA stability (Bikkavilli & Malbon, 2010) were significantly reduced in the basal cells (Fig 5b).

Third decision letter

MS ID#: DEVELOP/2020/199281

MS TITLE: Laminin alpha 5 Regulates Mammary Gland Remodeling Through Luminal Cell Differentiation and Wnt4 Mediated Epithelial Crosstalk

AUTHORS: Johanna I Englund, Alexandra Ritchie, Leander Blaas, Hanne Cojoc, Nalle Pentinmikko, Julia Dohla, Sharif Iqbal, Manuel Patarroyo, and Pekka Katajisto

© 2021. Published by The Company of Biologists under the terms of the Creative Commons Attribution License (https://creativecommons.org/licenses/by/4.0/).

I have now received all the referees reports on the above manuscript, and have reached a decision. The referees' comments are appended below, or you can access them online: please go to BenchPress and click on the 'Manuscripts with Decisions' queue in the Author Area.

The overall evaluation is positive and we would like to publish a revised manuscript in Development, provided that the referees' comments can be satisfactorily addressed. Please attend to all of the reviewers' comments in your revised manuscript and detail them in your point-by-point response. If you do not agree with any of their criticisms or suggestions explain clearly why this is so. Your manuscript will not require re-review, rather I will look it over myself prior to acceptance.

We are aware that you may currently be unable to access the lab to undertake experimental revisions. If it would be helpful, we encourage you to contact us to discuss your revision in greater detail. Please send us a point-by-point response indicating where you are able to address concerns raised (either experimentally or by changes to the text) and where you will not be able to do so within the normal timeframe of a revision. We will then provide further guidance. Please also note that we are happy to extend revision timeframes as necessary.

Reviewer 1

Advance summary and potential significance to field

The manuscript by England et al. investigates the role of laminin alpha-5 in mammary gland development. After analysing the expression of different laminin alpha chains by in situ hybridization, the authors focus on Lama5, mostly (but not exclusively) expressed by luminal cells. The main body of the paper describes the mammary phenotype of the Krt8cre-ert2/Lama5flox/flox mice, in which the alpha-5 laminin chain is conditionally deleted from luminal cells of the mammary epithelium after tamoxifen injection. Ductal development is perturbed when deletion is induced in pre-pubertal females while alveologenesis and lactogenic differentiation appear impaired during pregnancy and lactation. Organoid culture analyses provide a potential mechanistic explanation to this mammary phenotype implicating paracrine luminal-basal interactions involving Wnt4 signalling.

Numerous works have reported the involvement of integrin receptors and their downstream signalling effectors in postnatal mammary gland development and differentiation. However, little is know about the in vivo role of laminins, expected to be major in vivo ligands of integrins in the mammary epithelium, given their abundance in the mammary basement membrane. The present study reveals interesting data concerning this item, although some important questions should be addressed prior to publication.

Comments for the author

I appreciate the addition of some previous studies describing the role of integrins in mammary alveologenesis. However there is a mistake in one of the references: the work by di Cicco et al. does not study at all this subject. Instead, please add : Romagnoli et al., 2020 (doi: 10.1242/dev.181552)

Reviewer 2

Advance summary and potential significance to field
N/A

Comments for the author

Regarding the lactation comment. The reviewer thanks the authors for partially taking this advice on board and adjusting the claims in their manuscript.
They comment:
"...it is somewhat unclear to us what reviewer precisely means by “lactation experiments”. As mentioned above, the manuscript of course contains multiple types of data related to the functionalization of the gland (Figure 4E-K) that are downstream relevant for lactation – even

© 2021. Published by The Company of Biologists under the terms of the Creative Commons Attribution License (https://creativecommons.org/licenses/by/4.0/).

though we will not make that point in the revised version. In case the reviewer actually refers to these data, we hope a specific suggestion relating to these data would be made"
To clarify:
'Lactation experiments' in this review refer to data collected from the mammary gland of a female dam during lactation. The authors label this 'PP2' and quantify these glands.
To be extremely clear: All data collected after littering in experiments where litter size was not standardized should be removed from the manuscript or replaced with results from standardized litters.
Regarding the comment on statistics.
The reviewer thanks the authors for clarifying in the legend that they analyzed more than one image from some mice. The question is: do the authors consider two images from a single animal to be independent replicates for statistical analyses?

Reviewer 3

Advance summary and potential significance to field

This paper describes a role for Lama5 in mammary gland development, with an interesting finding that it is mainly produced by luminal cells.

Comments for the author

I only had a single remaining comment after reading the previous version and the authors have sufficiently nuanced their statement, so I don't have anything further to add (I still think they should be careful about extrapolating findings/target genes from one tissue to the next).

Fourth revision

Author response to reviewers' comments
We thank all of the reviewers for their constructive comments. Please find our point-to-point responses to reviewer comments below (in italics).

Reviewer 1 Advance Summary and Potential Significance to Field

The manuscript by England et al. investigates the role of laminin alpha-5 in mammary gland development. After analysing the expression of different laminin alpha chains by in situ hybridization, the authors focus on Lama5, mostly (but not exclusively) expressed by luminal cells.

The main body of the paper describes the mammary phenotype of the Krt8cre-ert2/Lama5flox/flox mice, in which the alpha-5 laminin chain is conditionally deleted from luminal cells of the mammary epithelium after tamoxifen injection. Ductal development is perturbed when deletion is induced in pre-pubertal females while alveologenesis and lactogenic differentiation appear impaired during pregnancy and lactation. Organoid culture analyses provide a potential mechanistic explanation to this mammary phenotype implicating paracrine luminal-basal interactions involving Wnt4 signalling.

Numerous works have reported the involvement of integrin receptors and their downstream signalling effectors in postnatal mammary gland development and differentiation. However, little is know about the in vivo role of laminins, expected to be major in vivo ligands of integrins in the mammary epithelium, given their abundance in the mammary basement membrane. The present study reveals interesting data concerning this item, although some important questions should be addressed prior to publication.

© 2021. Published by The Company of Biologists under the terms of the Creative Commons Attribution License (https://creativecommons.org/licenses/by/4.0/).

Reviewer 1 Comments for the Author:
I appreciate the addition of some previous studies describing the role of integrins in mammary alveologenesis. However there is a mistake in one of the references: the work by di Cicco et al. does not study at all this subject. Instead, please add : Romagnoli et al., 2020 (doi: 10.1242/dev.181552)

We thank the reviewer for the constructive comments on our manuscript and pointing out this mistake in the reference list. We have changed the aforementioned reference as suggested.

Reviewer 2 Advance Summary and Potential Significance to Field

Reviewer 2 Comments for the Author:
Regarding the lactation comment. The reviewer thanks the authors for partially taking this advice on board and adjusting the claims in their manuscript.

They comment:
"...it is somewhat unclear to us what reviewer precisely means by “lactation experiments”. As mentioned above, the manuscript of course contains multiple types of data related to the functionalization of the gland (Figure 4E-K) that are downstream relevant for lactation – even though we will not make that point in the revised version. In case the reviewer actually refers to these data, we hope a specific suggestion relating to these data would be made"

To clarify:
‘Lactation experiments’ in this review refer to data collected from the mammary gland of a female dam during lactation. The authors label this 'PP2' and quantify these glands.

To be extremely clear: All data collected after littering in experiments where litter size was not standardized should be removed from the manuscript or replaced with results from standardized litters.

We thank the reviewer for clarifying this specific point. In the light of the reviewer comments, we have now decided to remove the data from Figure 4H-K quantifying the functional differentiation phenotype in Lama5 lacking mammary glands. We have however retained the data related to tissue architecture and polarity of the epithelium, yet and have added a statement in the text that litter size can affect our conclusions from the PP2 glands: 252 “However, the Lama5-deficient females often had less pups, which at this stage can also affect the gland morphology.”

Regarding the comment on statistics.
The reviewer thanks the authors for clarifying in the legend that they analyzed more than one image from some mice. The question is: do the authors consider two images from a single animal to be independent replicates for statistical analyses?

Here all individual images from different parts of the mammary glands are considered as independent replicates (coming from 5 +/+ and 6 fl/fl animals).

Reviewer 3 Advance Summary and Potential Significance to Field

This paper describes a role for Lama5 in mammary gland development, with an interesting finding that it is mainly produced by luminal cells.

Reviewer 3 Comments for the Author:
I only had a single remaining comment after reading the previous version and the authors have sufficiently nuanced their statement, so I don't have anything further to add (I still think they should be careful about extrapolating findings/target genes from one tissue to the next).

© 2021. Published by The Company of Biologists under the terms of the Creative Commons Attribution License (https://creativecommons.org/licenses/by/4.0/).

We thank the reviewer for the constructive remarks. The tissue dependent nature of Wnt-signaling is indeed an important point, and we do acknowledge it while describing the findings. In the light of multiple types of data indicating the importance of Wnt-signaling in the luminal-basal crosstalk, we feel our choice of genes for the validation experiments is justified.

Fourth decision letter

MS ID#: DEVELOP/2020/199281

MS TITLE: Laminin alpha 5 Regulates Mammary Gland Remodeling Through Luminal Cell Differentiation and Wnt4 Mediated Epithelial Crosstalk

AUTHORS: Johanna I Englund, Alexandra Ritchie, Leander Blaas, Hanne Cojoc, Nalle Pentinmikko, Julia Dohla, Sharif Iqbal, Manuel Patarroyo, and Pekka Katajisto

ARTICLE TYPE: Research Article

I am happy to tell you that your manuscript has been accepted for publication in Development, pending our standard ethics checks.

© 2021. Published by The Company of Biologists under the terms of the Creative Commons Attribution License (https://creativecommons.org/licenses/by/4.0/).
